# *Retro-*Curcuminoids as Mimics of Dehydrozingerone and Curcumin: Synthesis, NMR, X-ray, and Cytotoxic Activity

**DOI:** 10.3390/molecules22010033

**Published:** 2016-12-29

**Authors:** Marco A. Obregón-Mendoza, María Mirian Estévez-Carmona, Simón Hernández-Ortega, Manuel Soriano-García, María Teresa Ramírez-Apan, Laura Orea, Hugo Pilotzi, Dino Gnecco, Julia Cassani, Raúl G. Enríquez

**Affiliations:** 1 Instituto de Química, Universidad Nacional Autónoma de México, Circuito Exterior, Ciudad Universitaria, México, D.F. C.P. 04510, Mexico; obregonmendoza@yahoo.com.mx (M.A.O.-M.); mirianestevezc@gmail.com (M.M.E.-C.); shernandezortega@gmail.com (S.H.-O.); soriano@unam.mx (M.S.-G.); mtrapan@yahoo.com.mx (M.T.R.-A.); 2 Centro de Química, Benemérita Universidad Autónoma de Puebla, Puebla C.P. 72570, Mexico; maria.orea@correo.buap.mx (L.O.); iken18@hotmail.com (H.P.); gneccod@yahoo.com.mx (D.G.); 3 Departamento de Sistemas Biológicos, Universidad Autónoma Metropolitana, Unidad Xochimilco, México, D.F. C.P. 04960, Mexico

**Keywords:** *retro*-curcuminoids, curcuminoid, curcumin, α,β-unsaturated-ketone, dehydrozingerone

## Abstract

Curcumin and its derivatives have been extensively studied for their remarkable medicinal properties, and their chemical synthesis has been an important step in the optimization of well-controlled laboratory production. A family of new compounds that mimic the structure of curcumin and curcuminoids, here named *retro*-curcuminoids (**7**–**14**), was synthesized and characterized using 1D ^1^H- and ^13^C-NMR, IR, and mass spectrometry; the X-ray structure of **7**, **8**, **9**, **10**, **12**, **13**, and **14** are reported here for the first time. The main structural feature of these compounds is the reverse linkage of the two aromatic moieties, where the acid chloride moiety is linked to the phenolic group while preserving α, β-unsaturated ketone functionality. The cytotoxic screening of **7**, **8**, **9**, and **10** at 50 and 10 µg/mL was carried out with human cancer cell lines K562, MCF-7, and SKLU-1. Lipid peroxidation on rat brain was also tested for compounds **7** and **10**. Compounds **7**, **8**, and **10** showed relevant cytotoxic activity against these cancer cell lines, and **10** showed a protective effect against lipid peroxidation. The molecular resemblance to curcuminoids and analogs with *ortho* substituents suggests a potential source of useful bioactive compounds.

## 1. Introduction

Curcumin (diferuloylmethane), a natural product used in traditional medicine, has been extensively studied for its multiple pharmacological effects on biological systems, to understand its chemical properties, and to improve its poor bioavailability [1]. A large number of studies have described the multiple beneficial medicinal properties from different points of view [2]. Curcumin has been used as a naturally-occurring resource for the treatment of numerous inflammatory diseases, and studies have confirmed that it possesses antiproliferative activity against several tumor cells in vitro [3]. Previous reports have described curcumin as a potent antioxidant with free radical scavenging activity [4] and a well-described therapeutic agent against Alzheimer disease [5,6] or recently as a molecular probe in the diagnostic imaging technique near-infrared fluorescence (NIRF), which is useful in the monitoring of amyloid beta species on Alzheimer patients [7]. Moreover, several chemical analogs, derivatives, and precursors of curcumin, both natural and synthetic, have shown such activity and also action against inflammatory diseases [8,9].

**Scheme 1:**
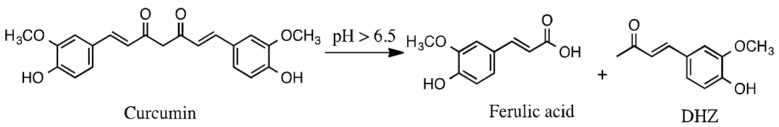
Degradation products of curcumin.

In 1973, Roughley and Whiting [10] reported the instability of curcumin in alkaline medium and explained its alkaline degradation. Later, Sardjiman and co-workers [11] reported that at pH higher than 6.5, curcumin decomposes into several molecular fragments, mainly ferulic acid and dehydrozingerone (DHZ), as shown in Scheme 1.

Ferulic acid, a precursor of the biosynthesis of curcumin, has been described as a compound with antitumoral, chemo-preventive, and photo-protective biological properties [12]. DHZ is a phenolic compound structurally related to biosynthesis or degradation of curcumin and is present as a minor component in the rhizomes of *Zingiber officinale* Roscoe (ginger). In numerous studies, DHZ has been shown to have radioprotective, antimicrobial, pro-healing, antioxidant, anti-Parkinson, anti-Alzheimer, antimutagenic, antitumor, anti-cancer, anti-depressant, anti-platelet, and antidiarrheal pharmacological activities [13,14,15,16].

Ferulic acid and DHZ are versatile chemical intermediates in organic synthesis with several bonding possibilities. We, therefore, decided to explore a modified linkage of such moieties involving the bond of the phenolic group of DHZ and related compounds, for example, acetylated ferulic acid [(*E*)-4-(3-chloro-3-oxoprop-1-en-1-yl)-2-methoxyphenyl acetate)], via the acid chloride derivative of the carboxylic functionalities and the corresponding phenolic group of the second moiety. The synthesis of what we call *retro-*curcuminoids was achieved by reaction of the corresponding acid chlorides with the appropriate phenolic moieties. We also explored the inhibition of lipoperoxidation and cytotoxic activity of the new compounds.

## 2. Results and Discussion

### 2.1. Structural Features

**Scheme 2:**
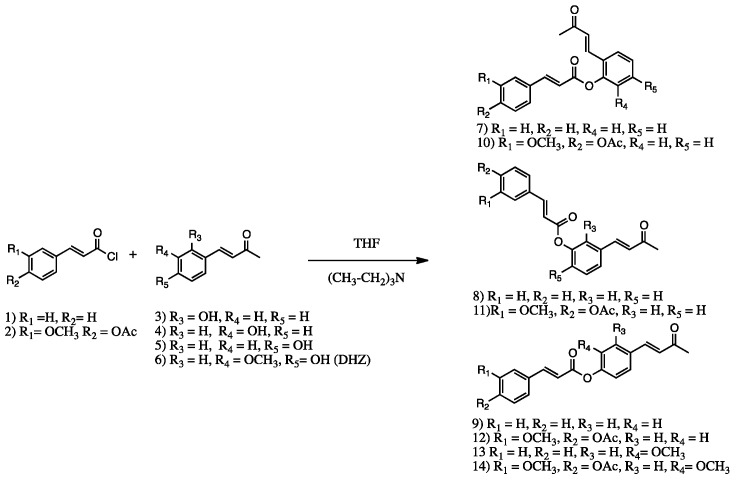
General synthesis.

*Retro-*curcuminoids **7**–**14** were prepared by esterification of cinnamoyl chloride (**1**) or (*E*)-4-(3-chloro-3-oxoprop-1-en-1-yl)-2-methoxyphenyl acetate (**2**) with DHZ [(*E*)-4-(4-hydroxy-3-methoxyphenyl)but-3-en-2-one] (**6**), and DHZ analogs (*E*)-4-(2-hydroxyphenyl)but-3-en-2-one, (*E*)-4-(3-hydroxyphenyl)but-3-en-2-one, and (*E*)-4-(4-hydroxyphenyl)but-3-en-2-one (**3**, **4**, and **5**), with *ortho*, *meta* and *para* hydroxyl substituents, respectively, as shown in Scheme 2.

### 2.2. Crystallography

**Figure 1:**
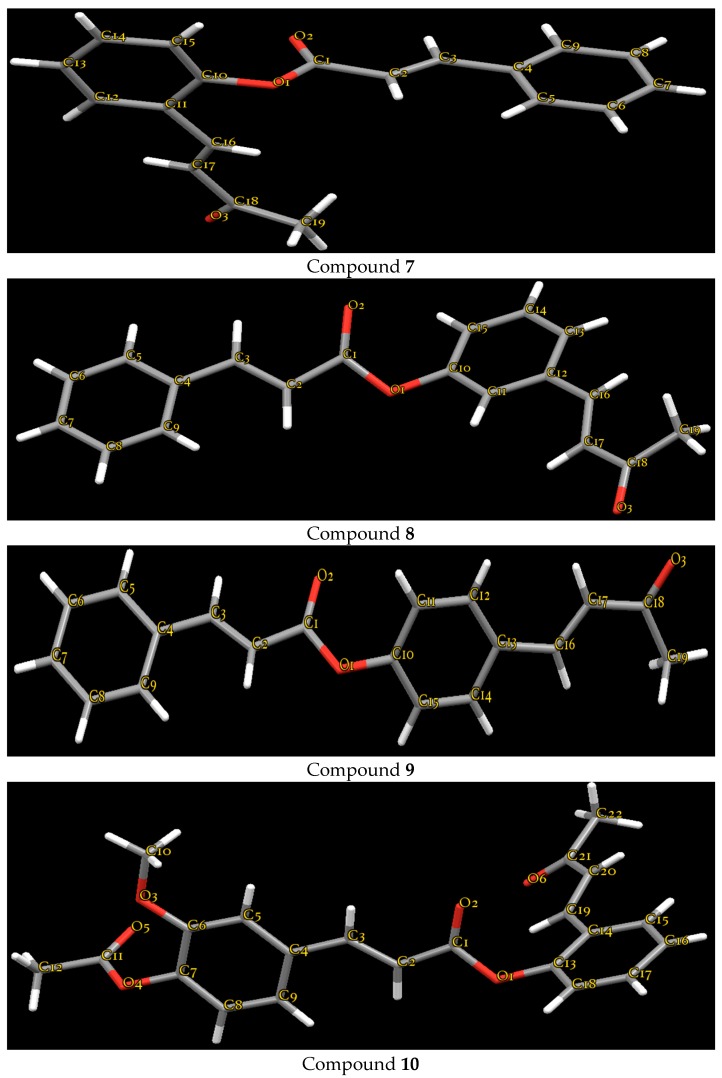

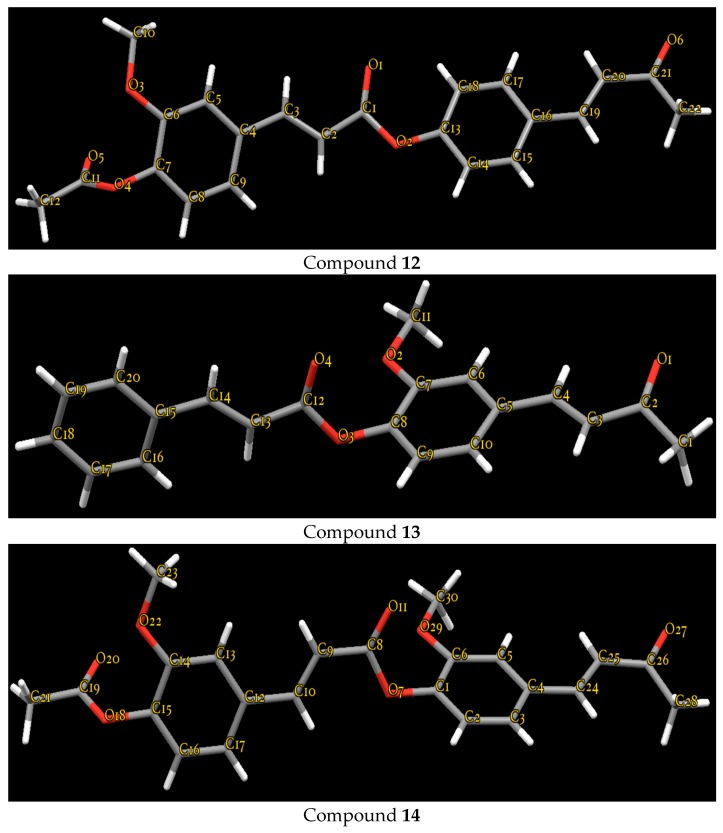
View of the molecular structure of compounds **7**, **8**, **9**, **10**, **12**, **13**, and **14**.

The molecular structures of all seven *retro*-curcuminoids are formed by two aromatic rings (phenyl-C-; phenyl-O-) connected by a moiety C(sp2)-C(sp2)-C(sp2)-C(sp2)-O(sp3)-C(sp2) chain of atoms (Figure 1).

The distance between the centers of gravity of phenyl Cg rings in structures **7**, **8**, **9**, **10**, **12**, **13**, and **14** remain constant independent of the type and location of ring substituents. These values are 8.795 Å, 8.807 Å, 8.795 Å, 8.794 Å, 8.830 Å, 8.747 Å, and 8.400 Å. Table 1 shows the crystal data, collection parameters and refinements for compounds **7**, **8**, **9**, **10**, **12**, **13** and **14**.

The crystal packing of the molecules in the crystal structures is stabilized by van der Waals interactions and linked by intermolecular C-H•••O hydrogen interactions. The adjacent molecules are interconnected by intermolecular weak C-H•••π interactions (from different Cg centers of gravity), which give additional support to molecular packing stability. Table 2 shows the intermolecular C-H•••O interactions in all seven crystal structures.

In the molecular structures of **7**, **8**, **9**, **10**, **12**, **13**, and **14**, the least-squares plane calculation shows that the dihedral angles between the two benzene rings are 80.7(1)°, 11.9(7)°, 61.6(5)°, 79.7(6)°, 38.7(1)°, 87.2(1)°, and 75.2(2)°, respectively. The dihedral angles between the C-phenyl-ring and –O-phenyl ring and the central moiety for structures **7**, **8**, **9**, **10**, **12**, **13**, and **14** are 3.2(1)° and 77.6(1)°, 37.8(1)° and 48.4(1)°, 2.2(1)° and 63.3(1)°, 2.9(1)° and 81.0(1)°, 13.2(1)° and 25.8(2)°, 3.2(1)° and 88.2(1)°, and 6.4(1)°, and 77.5(1)°, respectively. This clearly shows that in structures **7**, **9**, **10**, **13**, and **14**, the –C-phenyl is coplanar with the central moiety.

This result agrees with the dihedral angle of 8.2° between the two benzene rings in the structure of 1,7-bis(4-ethyloxy-3-methoxy-phenyl)-1,6-heptadiene-3,5-dione [17]. In contrast, the dihedral angle between the –O-phenyl ring and the central moiety varies from 63.3° to 88.2°. These features are shown in previous reports with α,β-unsaturated aromatic ester compounds [18,19,20,21,22], where aromatic, double bond, and ester groups are in a planar configuration and the substituents bonded to ester groups show some deviation. For structures **8** and **12** the dihedral angles show no significant difference.

### 2.3. Cytotoxicity and Antioxidant Activity

Cytotoxic activity of all synthesized compounds was first measured in healthy and cancerous cell lines. None of the compounds injured healthy cells; however, only compounds **7**, **8**, **9**, and **10** showed potential cytotoxic activity. Compounds **7**, **8**, and **10** (*ortho*, *meta*, and *ortho* substituents, respectively) had IC_50_ values similar to curcumin and displayed better anticancer activity against K562, MCF-7, and SKLU-1 cells than compound **9** (Table 3). These results agree with a previous study of the cytotoxicity of dehydrozingerone derivatives, which reported that compounds with hydroxyl at the *ortho* position, and ketone and phenyl at the C-3 and C-4 positions, respectively, are necessary for optimal cytotoxic activity [8]. The presence of α,β unsaturated systems, which has previously been described as an enhancer of cytotoxic activity, may also account for the biological effect of *retro*-curcuminoids. The ketone form in particular enhances toxicity [23], and the molecules we synthesized have double α,β unsaturated systems. The cytotoxic specificity of these new derivatives of curcumin and dehydrozingerone is related to precise differences in chemical structure, which could form the basis of future studies of the anticancer properties of *retro*-curcuminoids.

The effect on lipid peroxidation by compounds **7**, **8**, **9**, and **10** was tested on rat brain, but only *retro*-curcuminoid **7** showed a high degree of inhibition. The unique *retro*-curcuminoid with antioxidant activity in vitro was compound **10**, as shown in Table 4. In contrast with the reported high antioxidant activity of curcumin and derivatives [1], the *retro*-curcuminoids described here had no important prophylactic effect on the oxidative process, probably because of the structural occupation of phenyl groups and the loss of free hydroxyls, chemical groups related to activity against free radicals. Preliminary analysis of antioxidant activity in vitro by DPPH assay did not show antioxidant properties of any of the *retro*-curcuminoids. These data suggest that changes in the chemical structure of these compounds decrease antioxidant activity, but enhance their anticancer properties.

## 3. Experimental Section

### 3.1. General Information

All chemicals were reagent grade and were used as received. Solvents were purified by standard methods [24].

### 3.2. Physical Measurements

Melting points were determined on an Electrothermal IA9100 digital melting point apparatus (Bibby Scientific Limited, Staffordshire, UK) and are uncorrected. IR absorption spectra were recorded in the 4000–400 cm^−1^ range as KBr pellets on a Perkin Elmer 283-B spectrophotometer (PerkinElmer, Waltham, MA, USA). ^1^H- and ^13^C-NMR spectra were recorded in CDCl_3_ on a Bruker 500 MHz (Billerica, MA, USA) or a 600 MHz NMR Agilent technologies spectrometer equipped with a DD2 Oneprobe (Agilent Technology, Santa Clara, CA, USA), using tetramethylasilane (TMS) as internal reference. NMR spectra were processed with MESTRENOVA version 10.0.2 and can be found in the Supplementary Materials.

### 3.3. Synthetic Procedures

Compounds **1** and **2** were prepared in high yield from acetylated ferulic or cinnamic acid with thionyl chloride in CHCl_3_ containing a catalytic amount of dimethylformamide (DMF), and refluxed for 4 h according to the synthetic route reported [25,26]. The precursors **3**, **4**, **5**, and **6** were obtained using a Claisen-Schmidt reaction with acetone and corresponding aldehyde in equimolar amounts, and sodium hydroxide as a powdered base, in accordance with a published synthetic method [27].

#### *Retro-*Curcuminoids **7**–**14**

A solution of the corresponding acid chloride (**1** or **2**) (1.0 eq in anhydrous THF) was added to a solution of DHZ analog (**3**–**5**) or DHZ (**6**) (1.0 eq in anhydrous THF) at 0 °C containing triethylamine (1.2 eq). The reaction mixture was stirred at 30 °C until the disappearance of the starting material was observed by TLC. The reaction was quenched by the addition of aqueous NH_4_Cl, and the mixture was extracted with EtOAc (3 × 15 mL). The combined extracts were dried over anhydrous Na_2_SO_4_ and filtered, and the solvent was removed in vacuo. The resulting precipitate was treated with adequate solvents to give the corresponding crystalline product.

*2-((E)-3-oxobut-1-en-1-yl)phenyl cinnamate* (**7**). Obtained as light green needles From ethyl ether/*n*-hexane (90%), m.p. 138.9 °C. ^1^H-NMR (500 MHz, Chloroform-*d*) δ (ppm) 7.93 (dd, *J* = 16.1, 0.5 Hz, 1H), 7.72–7.58 (m, 4H), 7.49–7.41 (m, 4H), 7.30 (dddd, *J* = 7.9, 7.4, 1.2, 0.6 Hz, 1H), 7.22 (ddd, *J* = 8.2, 1.3, 0.4 Hz, 1H), 6.72 (dd, *J* = 18.8, 16.2 Hz, 2H), 2.34 (s, 3H). ^13^C-NMR (125 MHz, CDCl_3_) δ (ppm) 198.03, 165.02, 151.61, 149.65, 147.59, 136.58, 133.92, 131.31, 130.99, 129.04, 128.87, 128.45, 127.53, 127.34, 126.35, 123.19, 116.38, 27.62. IR; 3060.72 cm^−1^, 3030.22 cm^−1^, 1728.16 cm^−1^, 1634.69 cm^−1^, 1600.59 cm^−1^, 1133.75 cm^−1^, 984.56 cm^−1^. *m*/*z* [293^+^].

*3-((E)-3-oxobut-1-en-1-yl)phenyl cinnamate* (**8**). Light yellow needless from CH_2_Cl_2_/*n*-hexane (90%), m.p. 100 °C. ^1^H-NMR (500 MHz, Chloroform-*d*) δ (ppm) 7.89 (d, *J* = 16.0 Hz, 1H), 7.60 (dd, *J* = 16.0, 3.0 Hz, 2H), 7.53–7.35 (m, 7H), 7.22 (dt, *J* = 6.9, 2.3 Hz, 1H), 6.72 (d, *J* = 16.0 Hz, 1H), 6.64 (d, *J* = 16.0 Hz, 1H), 2.38 (s, 3H). ^13^C-NMR (125 MHz, CDCl_3_) δ (ppm) 198.10, 165.16, 151.25, 147.04, 142.25, 136.05, 134.02, 130.84, 130.59, 129.95, 129.06, 129.02, 128.34, 128.26, 127.92, 125.75, 123.66, 121.06, 116.90, 27.67. IR; 3052.19 cm^−1^, 2923.64 cm^−1^, 1717.09 cm^−1^, 1663.80 cm^−1^, 1631.63 cm^−1^, 1310.01 cm^−1^, 1257.60 cm^−1^, 1150.97 cm^1^, 977.46 cm^−1^. *m*/*z* [293^+^].

*4-((E)-3-oxobut-1-en-1-yl)phenyl cinnamate* (**9**). Colorless needles from EtOAc/*n*-hexane (90%), m.p. 139.15 °C. ^1^H-NMR (500 MHz, Chloroform-*d*) δ (ppm) 7.87 (dd, *J* = 16.0, 15.9 Hz, 2H), 7.63–7.47 (m, 4H), 7.48–7.37 (m, 3H), 7.29–7.20 (m, 2H), 6.66 (d, *J* = 16.0 Hz, 1H), 6.53 (d, *J* = 15.9 Hz, 1H), 2.38 (s, 3H). ^13^C-NMR (CDCl_3_). ^13^C-NMR (125 MHz, CDCl_3_) δ (ppm) 198.17, 164.96, 162.43, 152.44, 148.60, 147.08, 142.29, 133.98, 133.69, 132.03, 131.23, 130.84, 129.37, 129.03, 128.99, 128.54, 128.31, 127.13, 122.25, 116.84, 116.73, 27.56. IR; 3062.07 cm^−1^, 3027.85 cm^−1^, 2926.10 cm^−1^, 1726.81 cm^−1^, 1662.5 cm^−1^, 1629.55 cm^−1^, 1214.85 cm^−1^, 1140.63 cm^−1^, 978.60 cm^−1^. *m*/*z* [293^+^].

*2-((E)-3-oxobut-1-en-1-yl)phenyl(E)-3-(4-acetoxy-3-methoxyphenyl) acrylate* (**10**). Light green needles from ethyl ether/*n*-hexane (90%), m.p. 160 °C. ^1^H-NMR (500 MHz, Chloroform-*d*) δ (ppm) 7.88 (d, *J* = 16.0 Hz, 1H), 7.66 (dd, 2H), 7.49–7.41 (m, 1H), 7.33–7.17 (m, 4H), 7.10 (d, *J* = 8.0 Hz, 1H), 6.74 (d, *J* = 16.3 Hz, 1H), 6.64 (d, *J* = 16 Hz, 1H), 3.90 (s, 3H), 2.34 (s, 6H). ^13^C-NMR (125 MHz, Chloroform-*d*) δ (ppm) 198.02, 168.65, 164.89, 151.54, 149.58, 146.81, 142.04, 136.49, 132.80, 131.32, 128.82, 127.52, 127.30, 126.39, 123.41, 123.15, 121.69, 116.55, 111.53, 55.97, 27.69, 20.61. IR; 3072.19 cm^−1^, 3011.55 cm^−1^, 2956.45 cm^−1^, 1759.78 cm^−1^, 1733.54 cm^−1^, 1604.83 cm^−1^, 1258.74 cm^−1^, 1201.97 cm^−1^, 1120.02 cm^−1^, 984.99 cm^−1^. *m*/*z* [381^+^].

*3-((E)-3-oxobut-1-en-1-yl)phenyl(E)-3-(4-acetoxy-3-methoxyphenyl) acrylate* (**11**). Colorless needles from CH_2_Cl_2_/ethyl ether/*n*-hexane (90%), m.p. 101.6 °C. ^1^H-NMR (500 MHz, Chloroform-*d*) δ 7.84 (d, *J* = 16.0 Hz, 1H), 7.50 (d, *J* = 16.3 Hz, 1H), 7.47–7.36 (m, 3H), 7.25–7.15 (m, 3H), 7.10 (d, *J* = 8.1 Hz, 1H), 6.72 (d, *J* = 16.3 Hz, 1H), 6.58 (d, *J* = 15.9 Hz, 1H), 3.89 (s, 3H), 2.38 (s, 3H), 2.34 (s, 3H). ^13^C-NMR (126 MHz, CDCl_3_) δ (ppm) 198.07, 168.67, 164.98, 151.50, 151.17, 146.24, 142.18, 141.89, 136.06, 132.93, 129.94, 127.91, 125.77, 123.60, 123.38, 121.50, 121.00, 117.07, 111.48, 55.92, 27.64, 20.60. IR; 3070.16 cm^−1^, 2945.64 cm^−1^, 1762.35 cm^−1^, 1720.60 cm^−1^, 1611.18 cm^−1^, 1237.04 cm^−1^, 1191.03 cm^−1^, 1150.22 cm^−1^, 975.91 cm^−1^. *m*/*z* [381^+^].

*4-((E)-3-oxobut-1-en-1-yl)phenyl(E)-3-(4-acetoxy-3-methoxyphenyl) acrylate* (**12**). Colorless needles from EtOAc/*n*-hexane (88%), m.p. 172 °C. ^1^H-NMR (500 MHz, Chloroform-*d*) δ (ppm) 7.84 (d, *J* = 15.9 Hz, 1H), 7.60 (d, *J* = 8.3 Hz, 2H), 7.52 (d, *J* = 16.3 Hz, 1H), 7.26–7.15 (m, 4H), 7.10 (d, *J* = 8.1 Hz, 1H), 6.70 (d, *J* = 16.2 Hz, 1H), 6.58 (d, *J* = 15.9 Hz, 1H), 3.89 (s, 3H), 2.39 (s, 3H), 2.34 (s, 3H). ^13^C-NMR (125 MHz, CDCl_3_) δ (ppm) 198.11, 168.64, 164.78, 152.34, 151.47, 146.29, 142.21, 141.89, 132.89, 132.08, 129.37, 127.16, 123.38, 122.21, 121.50, 117.02, 111.46, 55.93, 27.58, 20.61. IR; 3066.07 cm^−1^, 2982.16 cm^−1^, 1757.33 cm^−1^, 1725.80 cm^−1^, 1632.44 cm^−1^, 1585.39 cm^−1^, 1256.04 cm^−1^, 1203.46 cm^−1^, 1166.33 cm^−1^, 1119.10 cm^−1^, 1012.64 cm^−1^, 974.26 cm^−1^. *m*/*z* [381^+^].

*2-Methoxy-4-((E)-3-oxobut-1-en-1-yl)phenyl cinnamate* (**13**). Obtained as yellow needles from EtOAc/*n*-hexane (80%), m.p. 132 °C. ^1^H-NMR (600 MHz, Chloroform-*d*) δ (ppm) 7.88 (d, *J* = 16.0 Hz, 1H), 7.59 (dd, *J* = 6.6, 2.9 Hz, 2H), 7.49 (d, *J* = 16.2 Hz, 1H), 7.42 (dd, *J* = 4.9, 1.9 Hz, 3H), 7.16 (d, *J* = 3.7 Hz, 3H), 6.67 (dd, *J* = 16.1, 9.1 Hz, 2H), 3.87 (s, 3H), 2.39 (s, 3H). ^13^C-NMR (125 MHz, CDCl_3_) δ (ppm) 198.10, 164.59, 151.59, 146.95, 145.68, 143.71, 142.67, 141.69, 134.06, 133.29, 130.71, 128.92, 128.29, 127.23, 123.40, 121.49, 116.57, 111.30, 55.92, 27.46. IR; 3069.35 cm^−1^, 3012.15 cm^−1^, 2933.29 cm^−1^, 1733.69 cm^−1^, 1254.52 cm^−1^, 1122.37 cm^−1^, 1030.06 cm^−1^, 971.39 cm^−1^. Calculated mass 322 (not observed).

*2-Methoxy-4-((E)-3-oxobut-1-en-1-yl)phenyl(E)-3-(4-acetoxy-3-methoxyphenyl) acrylate* (**14**). Colorless needles from ethyl ether/*n*-hexane (80%), m.p. 181 °C. ^1^H-NMR (600 MHz, Chloroform-*d*) δ (ppm) 7.84 (d, *J* = 15.9 Hz, 1H), 7.50 (d, *J* = 16.3 Hz, 1H), 7.17 (d, *J* = 7.3 Hz, 5H), 7.09 (d, *J* = 8.0 Hz, 1H), 6.68 (d, *J* = 16.3 Hz, 1H), 6.61 (d, *J* = 15.9 Hz, 1H), 3.89 (s, 6H), 2.40 (s, 3H), 2.34 (s, 3H); ^13^C-NMR (CDCl_3_) δ = 20.63, 27.30, 27.60, 55.90, 111.35, 116.70, 121.50, 123.40, 133.4, 141.70, 142.76, 146.29, 151.56, 164.60, 168.6, 198.3. IR; 3079.23 cm^−1^, 3010.78 cm^−1^, 1758.89 cm^−1^, 1722.39 cm^−1^, 1196.83 cm^−1^, 1152.87 cm^−1^, 1120.88 cm^−1^, 1027.43 cm^−1^ 978.07 cm^−1^. *m*/*z* [411^+^].

### 3.4. Crystallography

The X-ray data for **14** was collected by using an Agilent-Gemini-Atlas area-detector diffractometer with CuKα (1.54184 Å) radiation at room temperature (293 K). The data reduction was carried out using CrysAlisPro (v38.43), and the absorption corrections were applied using the CrysAlisPro analytical method [28]. Crystallographic data, together with refinement details for compound **14**, are deposited at the Cambridge Crystallographic Data Center (CCDC) (No. 1056387). The X-ray data for compounds **7**, **8**, **9**, **10**, **12**, and **13** were collected using a Bruker Smart APEX AXS CCD area detector with a graphite monochromator and Mo *Kα* radiation (λ = 0.71073 Å) at room temperature in scan mode. Collected data were reduced using SAINT [29] and empirical absorption corrections were performed using SADABS software [29,30]. Crystallographic data together with refinement details are summarized in Table 1.

All reflections were defined based on *F*^2^. The weighted *R*-factor *wR* and goodness of fit *S* are based on *F*^2^, and conventional *R*-factors *R* are based on *F*, with *F* set to zero for negative *F*^2^. The threshold expression of *F*^2^ > σ (*F*^2^) is used only for calculating *R*-factors (gt), etc., and is not relevant to the choice of reflections for refinement. *R*-factors based on *F*^2^ are statistically about twice as large as those based on *F*, and *R*-factors based on all data will be even larger. The crystallographic formulas for *R*_int_, *R*_1_, *w*R_2_ and *S* were used for refinement of structures.

$$R_{\mathrm{int}}=\frac{\sum \left|F_{o}^{2}-\left(F_{o}^{2}\right)\right|}{\sum F_{o}^{2}},\;R_{1}=\frac{\sum \|F_{o}|-|F_{c}\|}{\sum \left|F_{o}\right|},\;wR_{2}=\sqrt{\frac{\sum w{\left(F_{o}^{2}-F_{c}^{2}\right)}^{2}}{\sum w{\left(F_{o}^{2}\right)}^{2}}},\;S=\sqrt{\frac{\sum w{\left(F_{o}^{2}-F_{c}^{2}\right)}^{2}}{m-n}}$$

The hydrogen atoms attached to the carbon atoms were placed in their calculated positions and included in the isotropic refinement using the riding model with C–H = 0.93 Å (−CH) or 0.96 Å (−CH_3_) Å with U_iso_ (H) = 1.2 U_eq_ (C-H) and 1.5 U_eq_ (C-H_3_).

Figure 1 shows the molecular structures and atom-labeling scheme for compounds **7**, **8**, **9**, **10**, **12**, **13**, and **14**. All structures were drawn with 50% displacements using Mercury 3.7 software for Windows [31]. The geometry of the molecules was calculated using PARST software [32].

Atomic coordinates and displacement parameters, and bond distances and bond angles for all seven *retro*-curcuminoids are given as Supplementary Materials.

### 3.5. Cell Lines, Culture Medium, and Cytotoxicity Assay

All synthetized compounds and precursors DHZ and ferulic acid, were initially screened in vitro at doses of 10 μg/mL and 50 μg/mL against human polymorphonuclear and cancer cell lines: K562 (human chronic myelogenous leukemia), MCF-7 (human mammary adenocarcinoma), and SKLU-1 (human lung adenocarcinoma). No cytotoxic activity was detected for DHZ and ferulic acid, although some activity has been reported for these compounds with other cell lines. [12,13,14,15,16]. After selection by preliminary screening, the IC_50_ values of compounds **7**, **8**, **9**, and **10** were calculated in triplicate following standard protocols. Clearly, the ester groups in the *retro*-curcuminoids play an important role in the exertion of cytotoxic activity in vitro. Cell lines were supplied by U.S. National Cancer Institute (NCI). The human tumor cytotoxicity was determined using the protein-binding dye sulforhodamine B (SRB) in microculture assay to measure cell growth, as described in the protocols established by the NCI [33]. The cell lines were cultured in RPMI-1640 medium supplemented with 10% fetal bovine serum, 2 mM l-glutamine, 10,000 units/mL penicillin G sodium, 10,000 μg/mL streptomycin sulfate, 25 μg/mL amphotericin B (Invitrogen/Gibco™, Thermo Fisher Scientific, Waltham, MA, USA), and 1% non-essential amino acids (Gibco). They were maintained at 37 °C in a humidified atmosphere with 5% CO_2_. The viability of the cells used in the experiments exceeded 95% as determined with trypan blue.

Cytotoxicity after treatment with the test compounds of the normal and tumor cells was determined using the protein-binding dye sulforhodamine B (SRB) in microculture assay to measure cell growth, as described in a previous study [34]. The cells were removed from the tissue culture flasks by treatment with trypsin and diluted with fresh media. From these cell suspensions, 100 µL, containing 5000–10,000 cells per well, was pipetted into 96-well microtiter plates (Costar, Cambridge, MA, USA) and the material was incubated at 37 °C for 24 h in a 5% CO_2_ atmosphere. Subsequently, 100 μL of a solution of the compound obtained by diluting the stocks was added to each well. The cultures were exposed for 48 h to the compound at concentrations of 50 µg/mL and 10 µg/mL. After the incubation period, cells were fixed to the plastic substratum by the addition of 50 μL of cold 50% aqueous trichloroacetic acid. The plates were incubated at 4 °C for 1 h, washed with tap H_2_O, and air-dried. The trichloroacetic-acid-fixed cells were stained by the addition of 0.4% SRB. Free SRB solution was removed by washing with 1% aqueous acetic acid. The plates were air-dried, and the bound dye was solubilized by the addition of 10 mM unbuffered Tris base (100 μL). The plates were placed on a shaker for 10 min, and the absorption was determined at 515 nm using an enzyme-linked immunosorbent assay (ELISA) plate reader (Bio-Tek Instruments, Winooski, VT, USA).

### 3.6. Inhibition of Lipid Peroxidation on Rat Brain

*Animals*. Adult male Wistar rats (200–250 g) were provided by the Instituto de Fisiología Celular, Universidad Nacional Autónoma de México (UNAM). Procedures and care of animals were conducted in conformity with the Mexican Official Norm for Animal Care and Handling NOM-062-ZOO-1999. They were maintained at 23 ± 2 °C on a 12/12 h light-dark cycle with free access to food and water.

*Rat Brain Homogenate Preparation*. Animal sacrifice was carried out avoiding unnecessary pain. Rats were sacrificed with CO_2_. The cerebral tissue (whole brain), was rapidly dissected and homogenized in phosphate-buffered saline (PBS) solution (0.2 g of KCl, 0.2 g of KH_2_PO_4_, 8 g of NaCl, and 2.16 g of NaHPO_4_·7H_2_O/L, pH adjusted to 7.4) as described elsewhere [35,36] to produce a 1/10 (*w*/*v*) homogenate. The homogenate was then centrifuged for 10 min at 800 rcf (relative centrifugal field) to yield a pellet that was discarded. The supernatant protein content was measured using Folin and Ciocalteu’s phenol reagent [37] and adjusted with PBS at 2.666 mg of protein/mL.

Induction of lipid peroxidation and thiobarbituric acid reactive substances (TBARS) quantification. As an index of lipid peroxidation, TBARS levels were measured using rat brain homogenates according to the method described by Ng and co-workers [38], with some modifications. Supernatant (375 µL) was added with 50 µL of 20 µM EDTA and 50 µL of each sample concentration dissolved in DMSO (50 µL of DMSO for control group) and incubated at 37 °C for 30 min. Lipid peroxidation was started adding 50 µL of freshly prepared 100 µM FeSO_4_ solution (final concentration 10 µM), and incubated at 37 °C for 1 h. The TBARS content was determined as described by Ohkawa and co-workers [39], with some modifications. 500 µL of TBA reagent (1% 2-thiobarbituric acid in 0.05 N NaOH and 30% trichloroacetic acid, in 1:1 proportion) was added to each tube and the final suspension was cooled on ice for 10 min, centrifuged at 13,400 rcf for 5 min and heated at 80 °C in a water bath for 30 min. After cooling at room temperature, the absorbance of 200 µL of supernatant was measured at λ = 540 nm in a UltraMicroplate (Synergy/HT BIOTEK Instrument, Inc., Winooski, VT, USA). The concentration of TBARS was calculated by interpolation on a standard curve of tetra-methoxypropane (TMP) as a precursor of MDA [40]. Results were expressed as nmoles of TBARS per mg of protein. The inhibition ratio (IR [%]) was calculated using the formula IR = (C – E) × 100/C, where C is the control absorbance and E is the sample absorbance. Butylated hydroxytoluene (BHT) and α-tocopherol were used as positive standards. All data were represented as mean ± standard error (SEM). Data were analyzed by one-way analysis of variance (ANOVA) followed by Dunnett’s test for comparison against control. Values of *p* ≤ 0.05 (*) and *p* ≤ 0.01 (**) were considered statistically significant.

## 4. Conclusions

Seven new *retro*-curcuminoids were synthesized with a chemical modification strategy in good yield, and NMR data confirmed the formation of the ester in *ortho*, *meta*, and *para* phenol positions. The crystalline descriptions indicate that the compounds are formed by two aromatic rings (phenyl-C–; phenyl-O–) connected by a moiety C(sp2)-C(sp2)-C(sp2)-C(sp2)-O(sp3)-C(sp2) chain of atoms, and –C-phenyl is coplanar with the central moiety. *Retro*-curcuminoids with *ortho* substituents had the highest cytotoxic activity. Only 2-((*E*)-3-oxobut-1-en-1-yl)phenyl(*E*)-3-(4-acetoxy-3-methoxyphenyl) acrylate showed relevant antioxidant activity. Comparison of the cytotoxicity of *retro*-curcuminoids with their synthetic precursors allows us to postulate that the ester function plays an important role in biological activity in vitro. The results of this study suggest DHZ-based derivatives in the *ortho* position as a new template for future anticancer and antioxidant studies of this family of compounds.

## Figures and Tables

**Table 1:** Crystal data, collection parameters, and refinements for compounds **7**–**10** and **12**–**14**.

Compound	7	8	9	10	12	13	14
CCDC deposit No.	1452007	1451557	1451621	1451555	1451617	1451556	1056387
Crystal size (mm)	0.37 × 0.05 × 0.03	0.30 × 0.20 × 0.09	0.39 × 0.19 × 0.13	0.37 × 0.11 × 0.11	0.31 × 0.12 × 0.05	0.35 × 0.21 × 0.15	Not determined
Color/shape	Light green/needles	Light yellow/needles	Colorless/prism	Light green/needles	Colorless/prism	Light yellow/needles	Colorless/prism
Empirical formula	C_19_H_16_O_3_	C_19_H_16_O_3_	C_19_H_16_O_3_	C_22_H_20_O_6_	C_22_H_20_O_6_	C_20_H_18_O_4_	C_23_H_22_O_7_
Formula weight	292.32	292.32	292.32	380.38	380.38	322.34	410.40
*Crystal system*	Orthorhombic	Monoclinic	Monoclinic	Monoclinic	Monoclinic	Triclinic	Monoclinic
Space group	*P* 2_1_2_1_2_1_	*P* 2_1_/c	*P* 2_1_/c	*P* 2_1_/c	*P* 2_1_/c	*P*-1	*P* 2_1_/c
*Unit cell dimensions*							
*a*, Å	5.7012(2)	33.3788(14)	19.2677(6)	6.07840(10)	22.6222(13)	7.9343(10)	5.9945(4)
*b*, Å	15.1134	5.7488(2)	4.10240(10)	17.3377(4)	7.6843(4)	10.0440(14)	36.2260(19)
*c*, Å	18.1035	8.0128(4)	21.2526(6)	18.3150(5)	10.9077(7)	11.9990(16)	9.6239(8)
α, deg	90.00	90.00	90.00	90.00	90.00	70.652(4)	90.00
β, deg	90.00	95.736(2)	115.0910(10)	90.5980(10)	95.314(2)	71.023(4)	91.083(6)
γ, deg	90.00	90.00	90.00	90.00	90.00	89.865(5)	90.00
Volume, Å^3^	1559.88(10)	1529.86(11)	1521.36	1930.03(8)	1888.00(19)	847.2(2)	2089.5(2)
*Z*	4	4	4	4	4	2	4
Density (calculated), Mg/m^3^	1.245	1.269	1.276	1.309	1.338	1.264	1.305
Absorption coefficient, mm^−1^	0.084	0.085	0.086	0.095	0.098	0.088	0.806
*F*(000)	616	616	616	800	800	340	864
Θ range for data collection (°)	2.25 to 25.39	2.45 to 25.31	1.94 to 25.40	2.22 to 25.50	2.71 to 25.32	2.16 to25.33	Not determined
Index ranges	−6 ≤ η ≤ 6, −17 ≤ κ ≤ 17, −21 ≤ λ ≤ 21	−39 ≤ η ≤ 39, −5 ≤ κ ≤ 5, −9 ≤ λ ≤ 9	−23 ≤ η ≤ 23, −4 ≤ κ ≤ 4, −25 ≤ λ ≤ 25	−7 ≤ *h* ≤ 7, −20 ≤ *k* ≤ 20, −22 ≤ *l* ≤ 22	−27 ≤ *h* ≤ 27, −9 ≤ *k* ≤ 9, −13 ≤ *l* ≤ 13	−9 ≤ *h* ≤ 9, −12 ≤ *k* ≤ 12, −14 ≤ *l* ≤ 14	−7 ≤ *h* ≤ 6, −44 ≤ *k* ≤ 44, −11 ≤ *l* ≤ 11
Reflections collected	13,670	20,778	16,473	26,381	22,547	12,126	20,379
Independent reflections	2857 (*R*_int_ = 0.1503)	2764 (*R*_int_ = 0.1323)	2781 (*R*_int_ = 0.0559)	3585 (*R*_int_ = 0.0925)	3433 (*R*_int_ = 0.1724)	3065 (*R*_int_ = 0.0631)	4128
Data/restraints/parameters	2857/154/246	2764/0/200	2781/162/246	3585/0/257	3433/0/256	3065/0/219	4128/0/275
*Goodness-of-fit on* *F*^2^	0.749	1.028	1.052	0.963	1.010	1.046	1.020
Final *R* indices [(*I*) > 2σ(*I*)]	*R*1 = 0.0472, *wR*2 = 0.0486	*R*1 = 0.0494, *wR*2 = 0.1162	*R*1 = 0.0489, *wR*2 = 0.1271	*R*1 = 0.0461, *wR*2 = 0.1072	*R*1 = 0.0606, *wR*2 = 0.1255	*R*1 = 0.0632, *wR*2 = 0.1629	*R*1 = 0.0620, *wR*2 = 0.1162
*R* indices (all data)	*R*1 = 0.2148, *wR*2 = 0.0749	*R*1 = 0.0816, *wR*2 = 0.1361	*R*1 = 0.0676, *wR*2 = 0.1422	*R*1 = 0.0851, *wR*2 = 0.1267	*R*1 = 0.1493, *wR*2 = 0.11618	*R*1 = 0.0888, *wR*2 = 0.1831	*R*1 = 0.1574, *wR*2 = 0.1945
Largest diff. peak and hole (e/A^−3^)	0.168 and −0.141	0.140 and −0.171	0.156 and −0.227	0.279 and −0.258	0.195 and −0.185	0.435 and −0.190	0.305 and −0.160

**Table 2:** Geometry of the hydrogen bonding interactions (Å, °) for compounds **7**, **8**, **9**, **10**, **12**, **13**, and **14**.

Compound	D-H···*A*	D-H	H···*A*	D···*A*	D-H···*A*	Symmetry Code		
**7**	C3-H3•••O3	0.930(5)	2.790(2)	3.486(2)	132.5(4)	−*x* + 1	+*y* + 1/2,	−*z* + 1/2
	C9-H9•••O3	0.930(5)	2.749(1)	3.463(2)	134.3(4)	−*x* + 1	+*y* + 1/2	−*z* + 1/2
	C14-H14•••O2	0.930(5)	2.902(3)	3.571(6)	130.0(3)	*x* − 1/2	−*y* + 1/2 + 1	−*z*
	C17-H17•••O1	0.931(11)	2.740(3)	3.356(12)	124.5(7)	*x* + 1	+*y*	+*z*
**8**	C2-H2•••O2	0.930(2)	2.735(2)	3.665(2)	179.9(1)	*x*	+*y* − 1	+*z*
	C9-H9•••O2	0.929(2)	2.957(2)	3.590(3)	126.7(2)	*x*	−*y* + 1/2	+*z* + 1/2
	C15-H15•••O2	0.931(2)	2.761(2)	3.543(2)	142.1(1)	*x*	−*y* + 1/2 + 1	+*z* − 1/2
**9**	C5-H5•••O3	0.930(2)	2.672(5)	3.539(5)	155.3(2)	−*x*	+*y* − 1/2	−*z* + 1/2
	C3-H3•••O3	0.930(2)	2.719(4)	3.588(4)	155.8(1)	−*x*	+*y* − 1/2	−*z* + 1/2
	C12-H12•••O2	0.930(2)	2.634(2)	3.554(3)	169.9(1)	−*x*	+*y* − 1/2	−*z* + 1/2
	C17-H17•••O2	0.930(3)	2.605(1)	3.534(3)	177.2(2)	−*x*	+*y* − 1/2	−*z* + 1/2
**10**	C10-H10A•••O2	0.981(2)	2.597(1)	3.124(2)	113.8(1)	−*x* + 2	−*y* + 2	−*z*
	C12-H12A•••O6	0.980(2)	2.661(2)	3.414(3)	133.9(2)	*x −* 1	−*y* + 1/2+1	*z* − 1/2
	C17-H17•••O6	0.951(2)	2.410(2)	3.183(3)	138.2(1)	−*x* + 2	+*y* + 1/2	−*z* + 1/2
	C20-H20•••O5	0.951(2)	2.484(2)	3.246(3)	137.1(2)	−*x* + 2	+*y* + 1/2	−*z* + 1/2
**12**	C18-H18•••O1	0.930(3)	2.358(2)	2.846(4)	112.5(2)	*x*	*y*	*z*
	C5-H5•••O6	0.931(3)	2.376(2)	3.267(4)	160.2(2)	−*x* + 1	−*y*	−*z* + 1
	C20-H20•••O1	0.929(3)	2.714(3)	3.431(4)	134.7(2)	−*x* + 1	−*y*	−*z* + 1
	C12-H12A•••O3	0.961(3)	2.680(2)	3.531(4)	147.9(2)	−*x* + 2	+*y* + 1/2	*z* + 1/2 + 2
	C12-H12B•••O5	0.959(3)	2.429(2)	3.372(4)	167.6(2)	−*x* + 2	+*y* + 1/2	*z* + 1/2 + 2
**13**	C6-H6•••O1	0.930(3)	2.417(2)	3.328(4)	166.0(2)	−*x*	−*y* + 1	−*z*
	C11-H11B•••O4	0.960(3)	2.825(2)	3.475(4)	125.8(2)	*x* − 1	+*y*	+*z*
	C18-H18•••O2	0.929(2)	2.718(2)	3.332(3)	124.3(1)	−*x*	−*y*	−*z* + 1
	C19-H19•••O2	0.930(4)	2.846(2)	3.397(4)	119.1(2)	−*x*	−*y*	−*z* + 1
**14**	C13-H13•••O11	0.929(3)	2.496(3)	3.413(4)	169.2(2) 1	−*x* + 1	−*y* + 1	−*z*
	C5-H5•••O20	0.930(3)	2.581(3)	3.504(4)	172.0(2) 2	−*x* + 1	−*y* + 1	−*z* + 1
	C25-H25•••O20	0.931(3)	2.600(2)	3.499(4)	162.6(2) 2	−*x* + 1	−*y* + 1	−*z* + 1
	C2-H2•••O22	0.930(3)	2.672(2)	3.485(4)	146.3(2) 4	−*x*	−*y* + 1	−*z*
	C23-H23C•••O18	0.960(4)	2.550(2)	3.475(4)	161.9(2) 5	−*x* + 1	+*y*	+*z*

**Table 3:** Cytotoxicity screening of *retro*-curcuminoids **7**, **8**, **9**, and **10**.

IC_50_ (µM) on Cell Line			
*Retro*-Curcuminoid	K562	MCF-7	SKLU-1
Curcumin	9.75 ± 0.6	10.43 ± 1.8	7.78 ± 0.64
**7**	10.74 ± 0.5	13.19 ± 1.4	11.59 ± 1.2
**8**	12.8 ± 1.9	18.68 ± 2.5	12.85 ± 0.9
**9**	28.15 ± 2.5 *	63.46 ± 10.8 *	63.81 ± 2.7 *
**10**	8.60 ± 0.44	12.01 ± 0.62	8.07 ± 0.55

Data are represented as mean ± standard error of mean (SEM). * *p* < 0.01 compared to curcumin. K562: leukemia adenocarcinoma cells, HCT-15: human colon cancer cells, MCF-7: human mammary adenocarcinoma cells, and SKLU-1: human lung adenocarcinoma cells.

**Table 4:** Inhibition of lipid peroxidation of *retro*-curcuminoids **7**, **8**, **9**, and **10** in rat brain.

*Retro*-Curcuminoid	Concentration (µg/mL)	% Inhibition
**7**	50	4.33 ± 1.06 *
	5	1.97 ± 0.77 *
**8**	50	1.77 ± 0.22 *
	5	0.27 ± 0.06 *
**9**	50	3.30 ± 0.78 *
	5	3.19 ± 0.53 *
**10**	50	96.18 ± 6.53
	5	14.81 ± 2.33

Data are represented as mean ± standard error of mean (SEM). * *p* < 0.01 compared to α-tocopherol.

## Supplementary Materials

Supplementary materials can be accessed at: http://www.mdpi.com/1420-3049/22/1/33/s1.

## Abbreviations

The following abbreviations are used in this manuscript:

DHZ	Dehydrozingerone
TPA	12-*O*-tetradecanoylphorbol-13-acetate
IC_50_	Inhibitory Concentration 50

## Appendix A

CCDC 1451617, 1451555, 1451556, 1451621, 1451557, and 1452007 contain the supplementary crystallographic data for C_19_H_16_O_3_ (**7**), C_19_H_16_O_3_ (**8**), C_19_H_16_O_3_ (**9**), C_22_H_20_O_6_ (**10**), C_22_H_20_O_6_ (**12**), and C_20_H_18_O_4_ (**13**). These data can be obtained free of charge via http://www.ccdc.cam.ac.uk/data_request/cif or from the Cambridge Crystallographic Data Centre, 12 Union Road, Cambridge CB2 1EZ, UK; Fax: +44-1223-336-033; or E-Mail: deposit@ccdc.cam.ac.uk.
