# Supplementary material for: Retro-Curcuminoids as Mimics of Dehydrozingerone and Curcumin: Synthesis, NMR, X-ray, and Cytotoxic Activity
Source: Molecules. 2016 Dec 29;22(1):33. doi: 10.3390/molecules22010033 (PMC6155822; doi:10.3390/molecules22010033)
Supplement: Supplementary file 1 [file molecules-22-00033-s001.pdf]

Supplementary Materials: *Retro-Curcuminoid*s as Mimics of Dehydrozingerone and Curcumin: Synthesis, NMR, X-ray, and Cytotoxic Activity

Marco A. Obregón-Mendoza, María Mirian Estévez-Carmona, Simón Hernández-Ortega, Manuel Soriano-García, María Teresa Ramírez-Apan, Laura Orea, Hugo Pilotzi, Dino Gnecco, Julia Cassani and Raúl G. Enríquez

IR *Retro*-Curcuminoid **7**

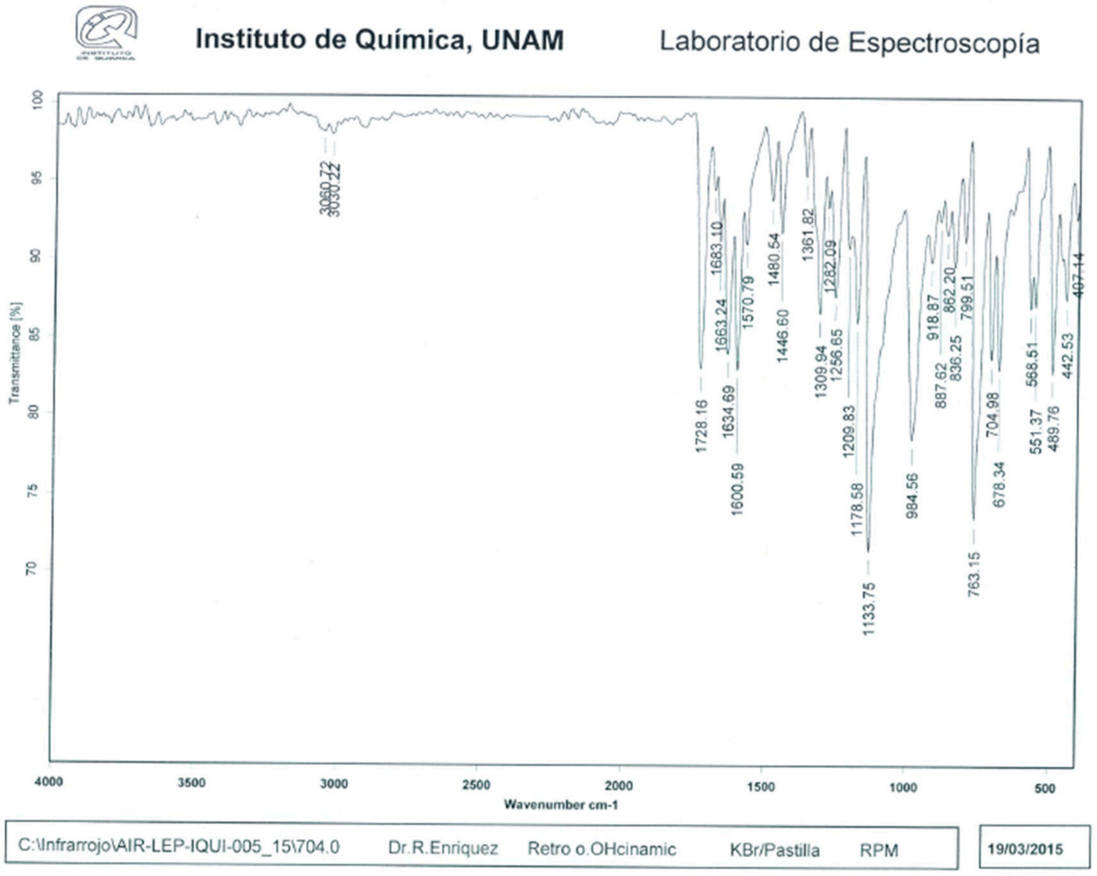

IR *Retro*-Curcuminoid **8**

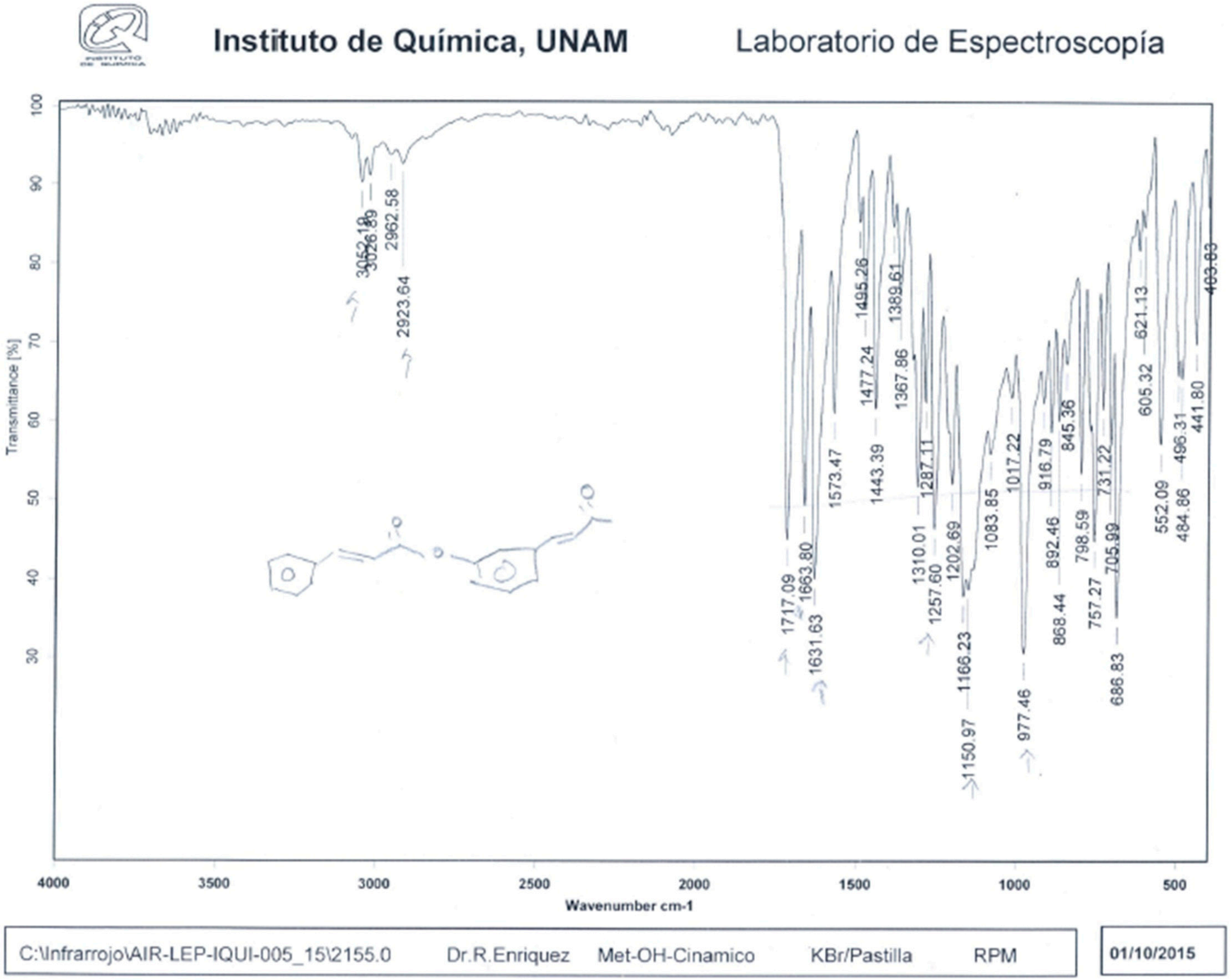

IR *Retro*-Curcuminoid **9**

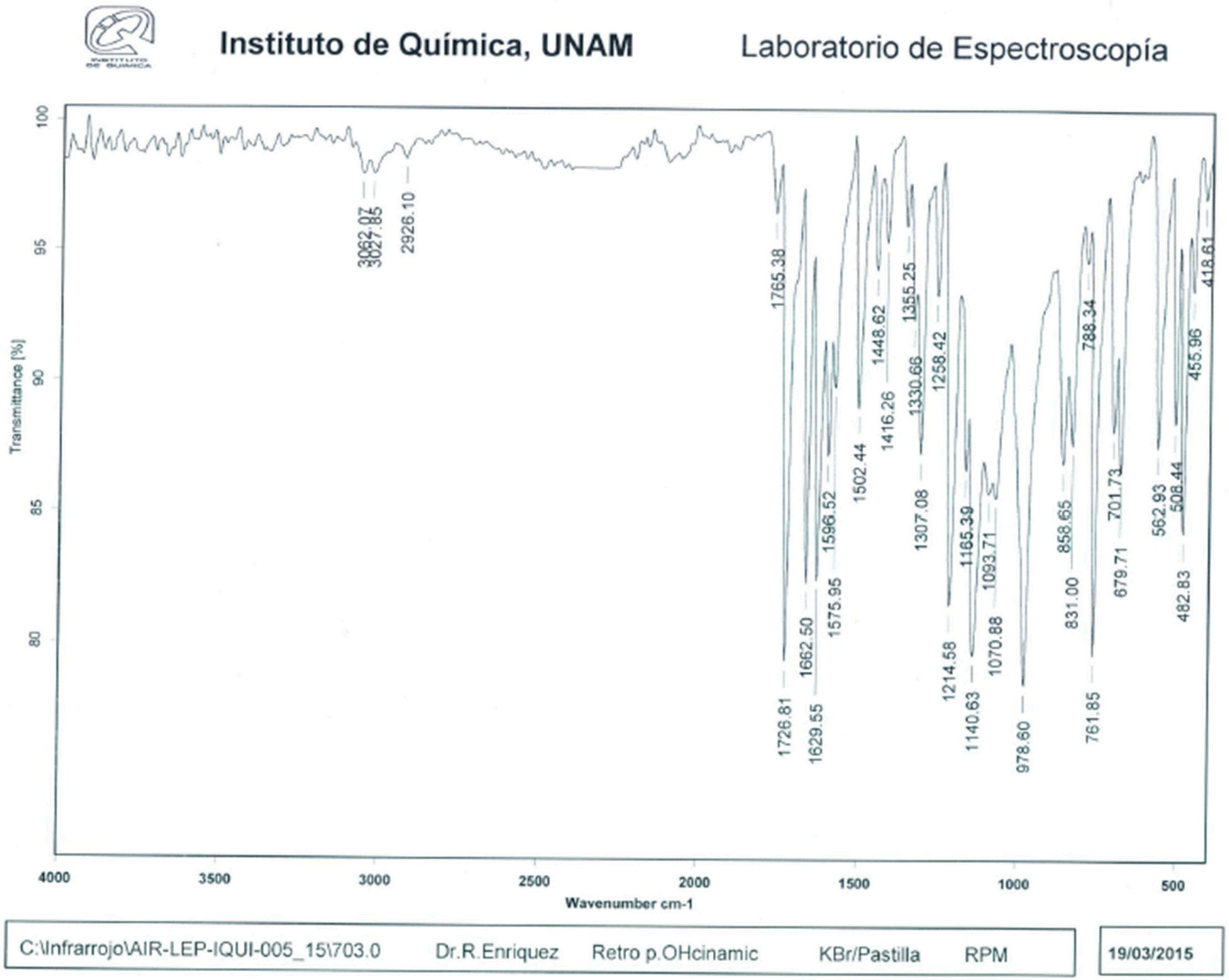

IR *Retro*-Curcuminoid **10**

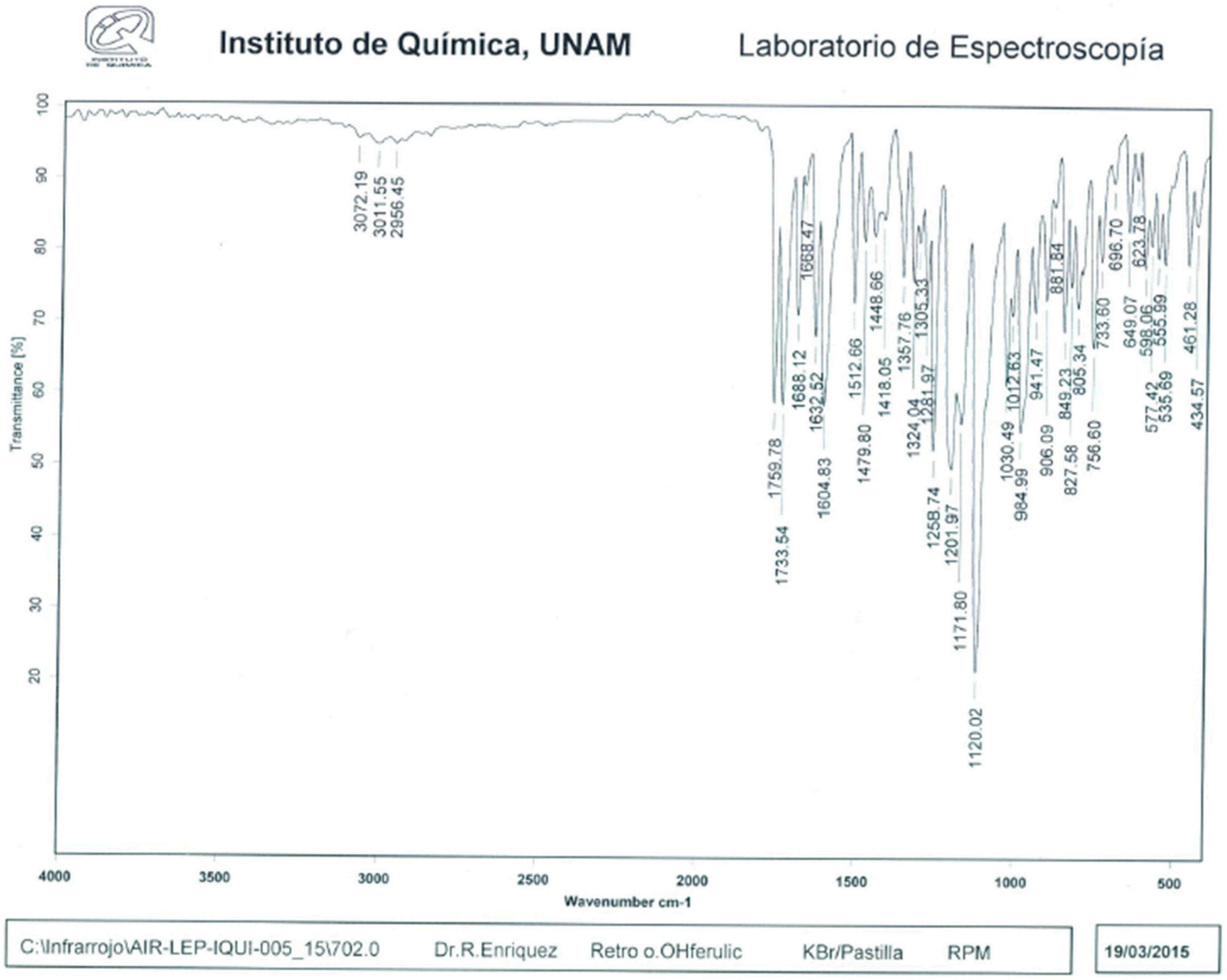

IR *Retro*-Curcuminoid **11**

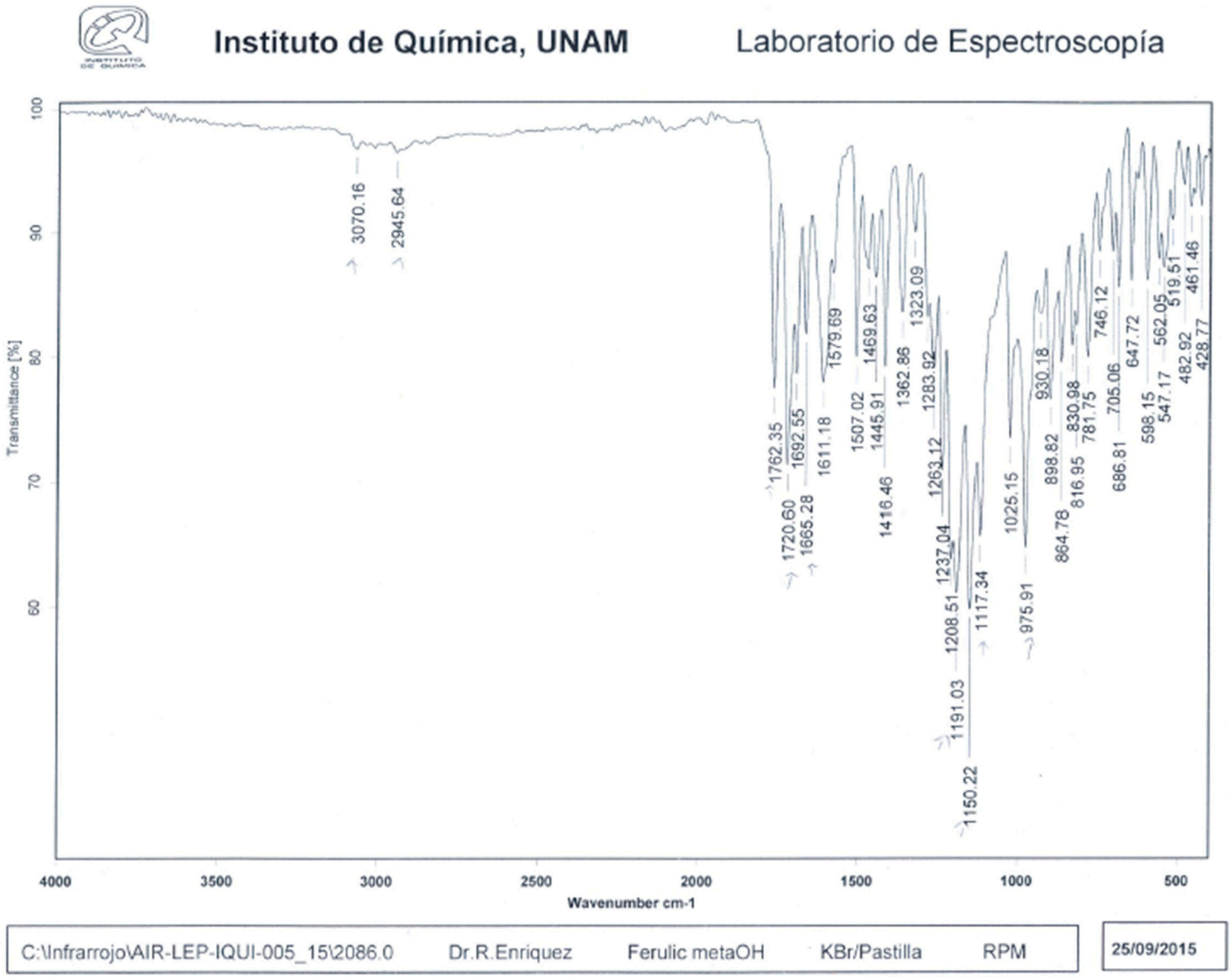

IR *Retro*-Curcuminoid **12**

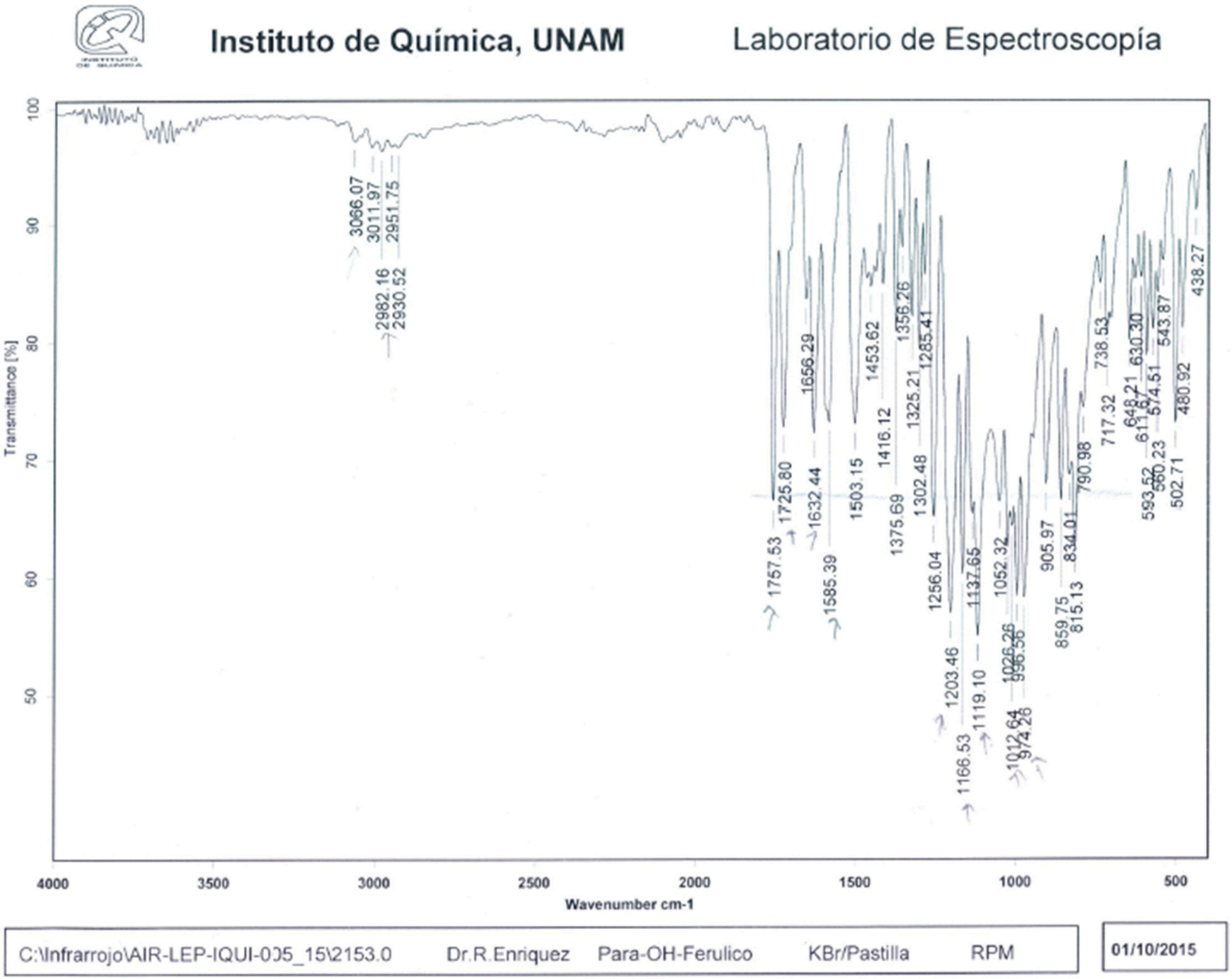

IR *Retro*-Curcuminoid **13**

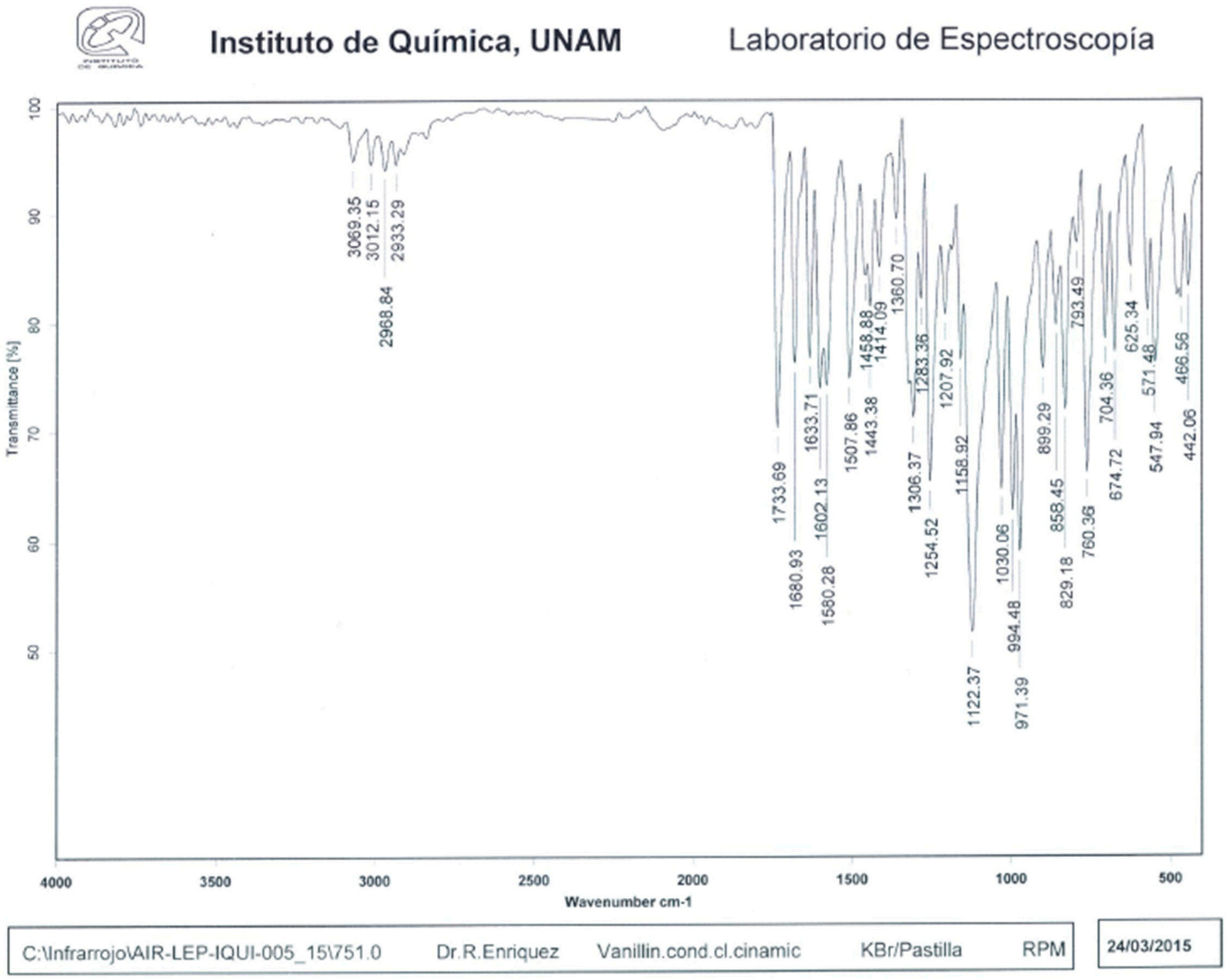

IR *Retro*-Curcuminoid **14**

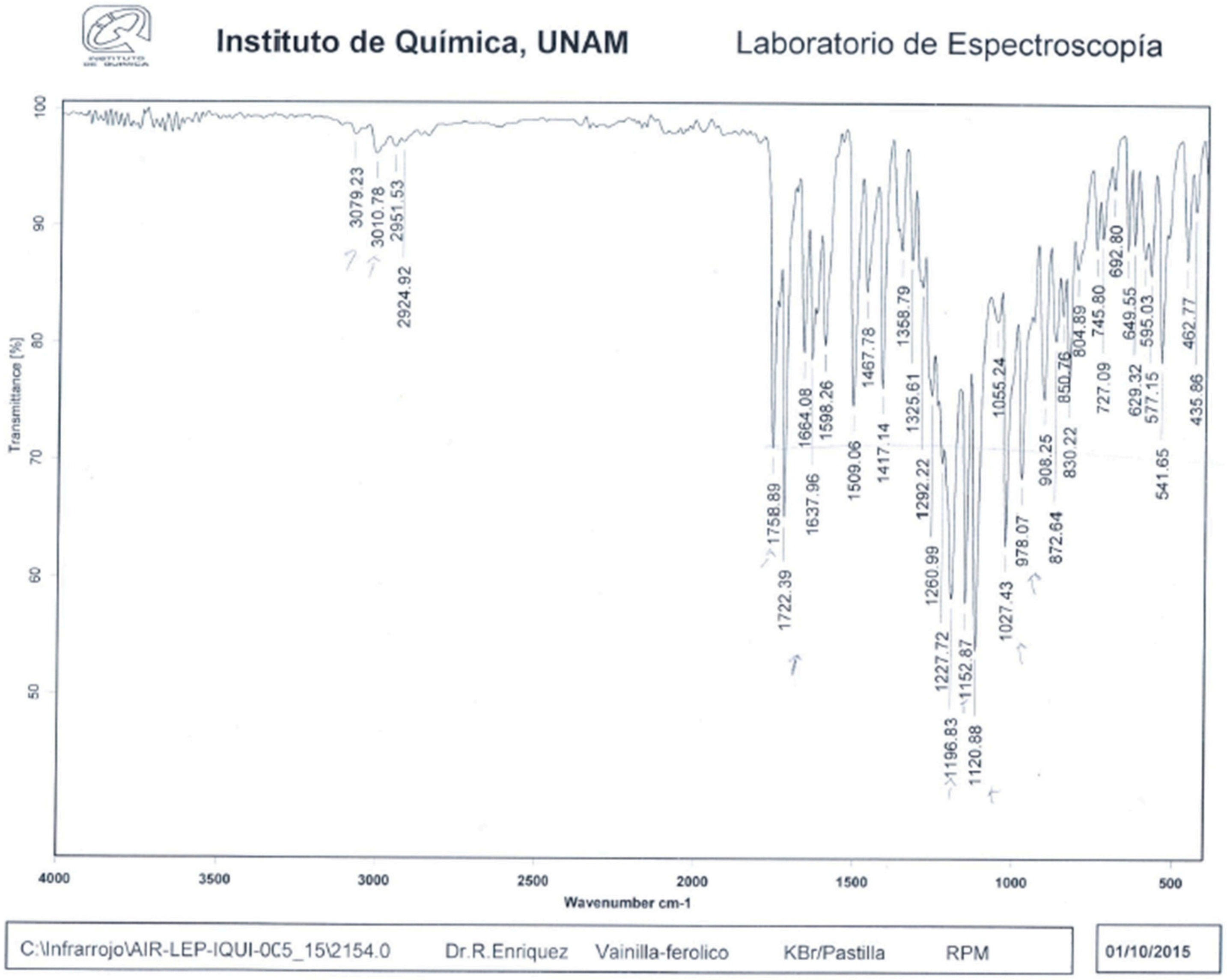

MS *Retro*-Curcuminoid **7**

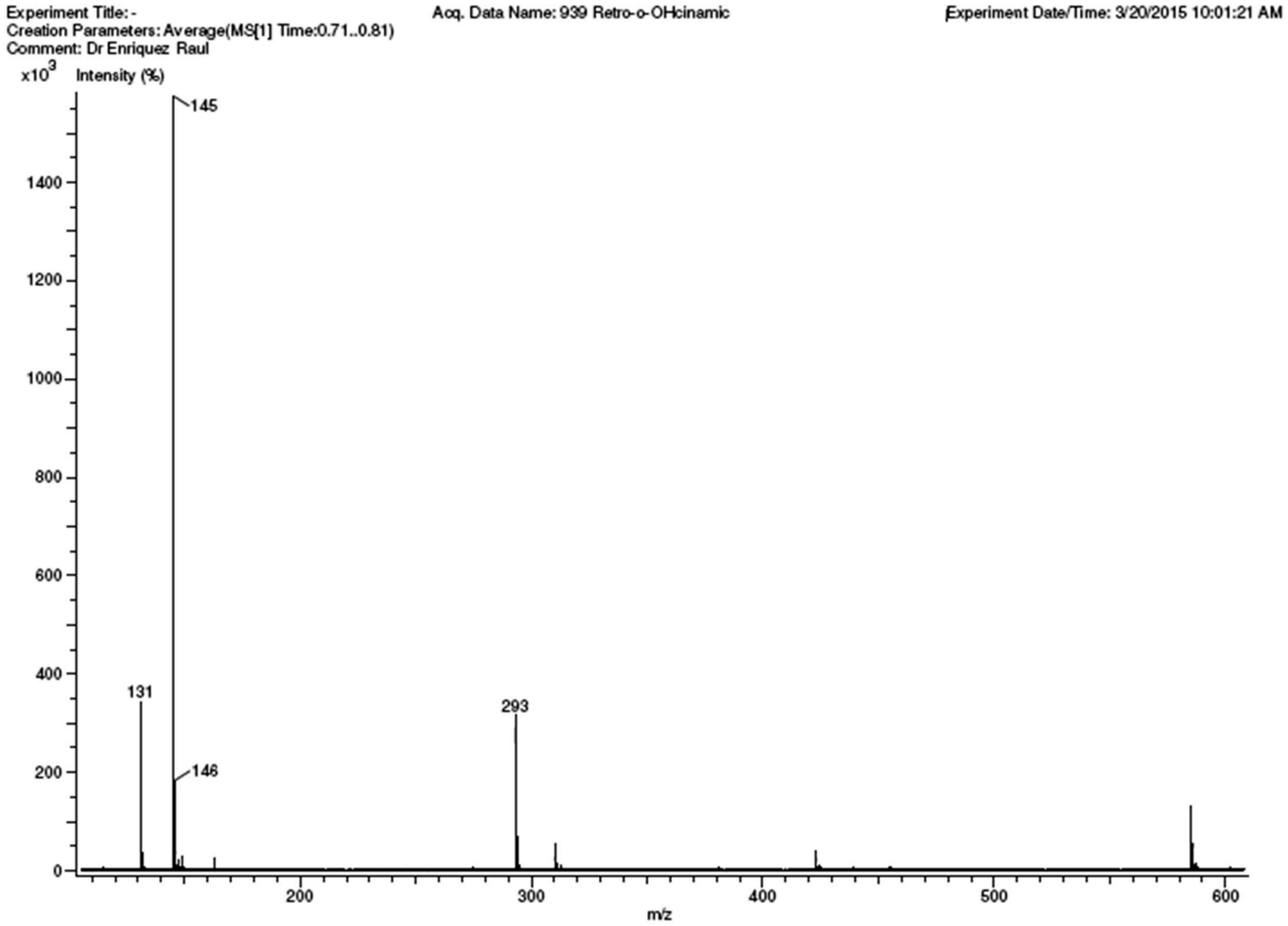

MS *Retro*-Curcuminoid **8**

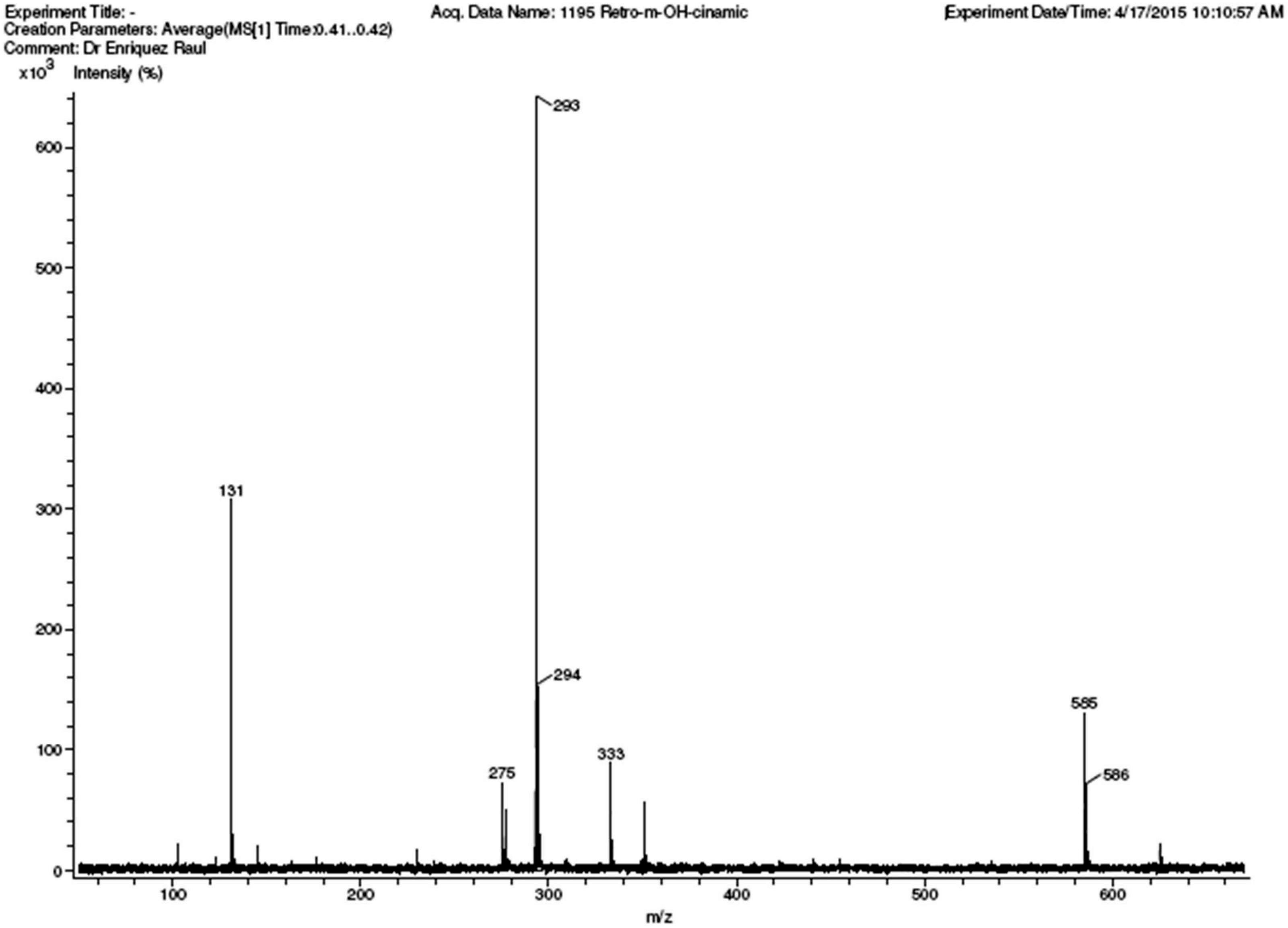

MS *Retro*-Curcuminoid **9**

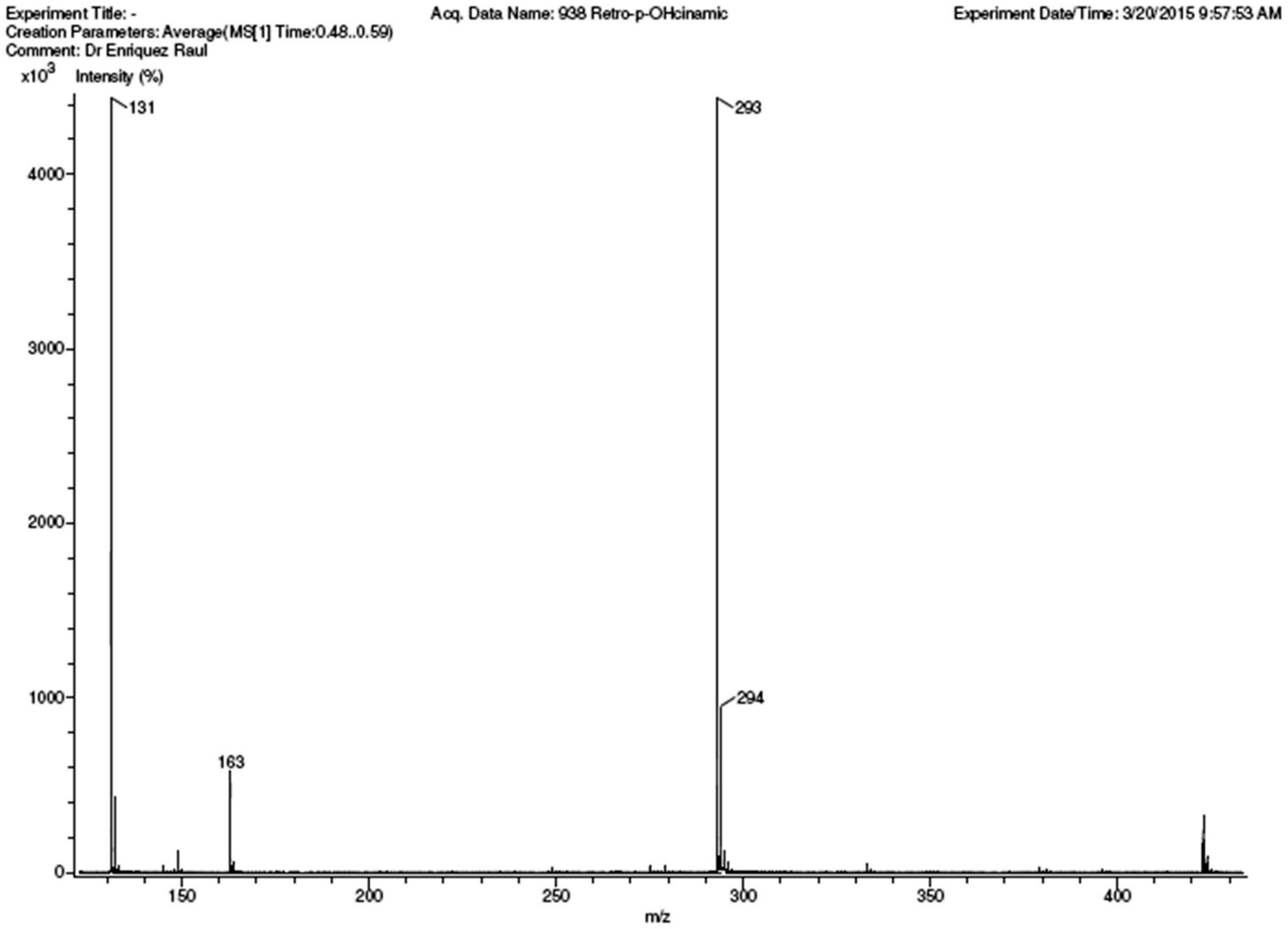

MS *Retro*-Curcuminoid **10**

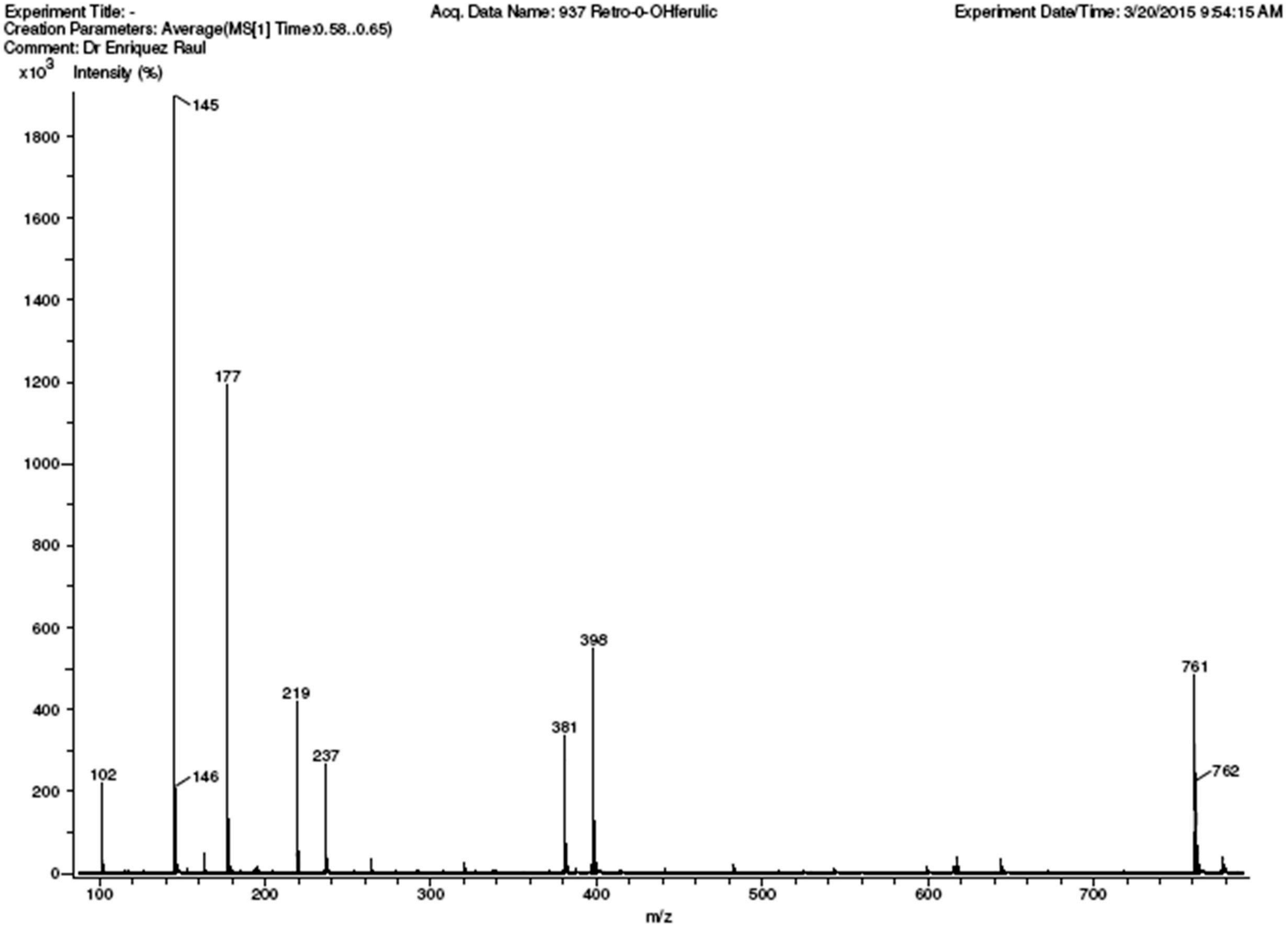

MS *Retro*-Curcuminoid **11**

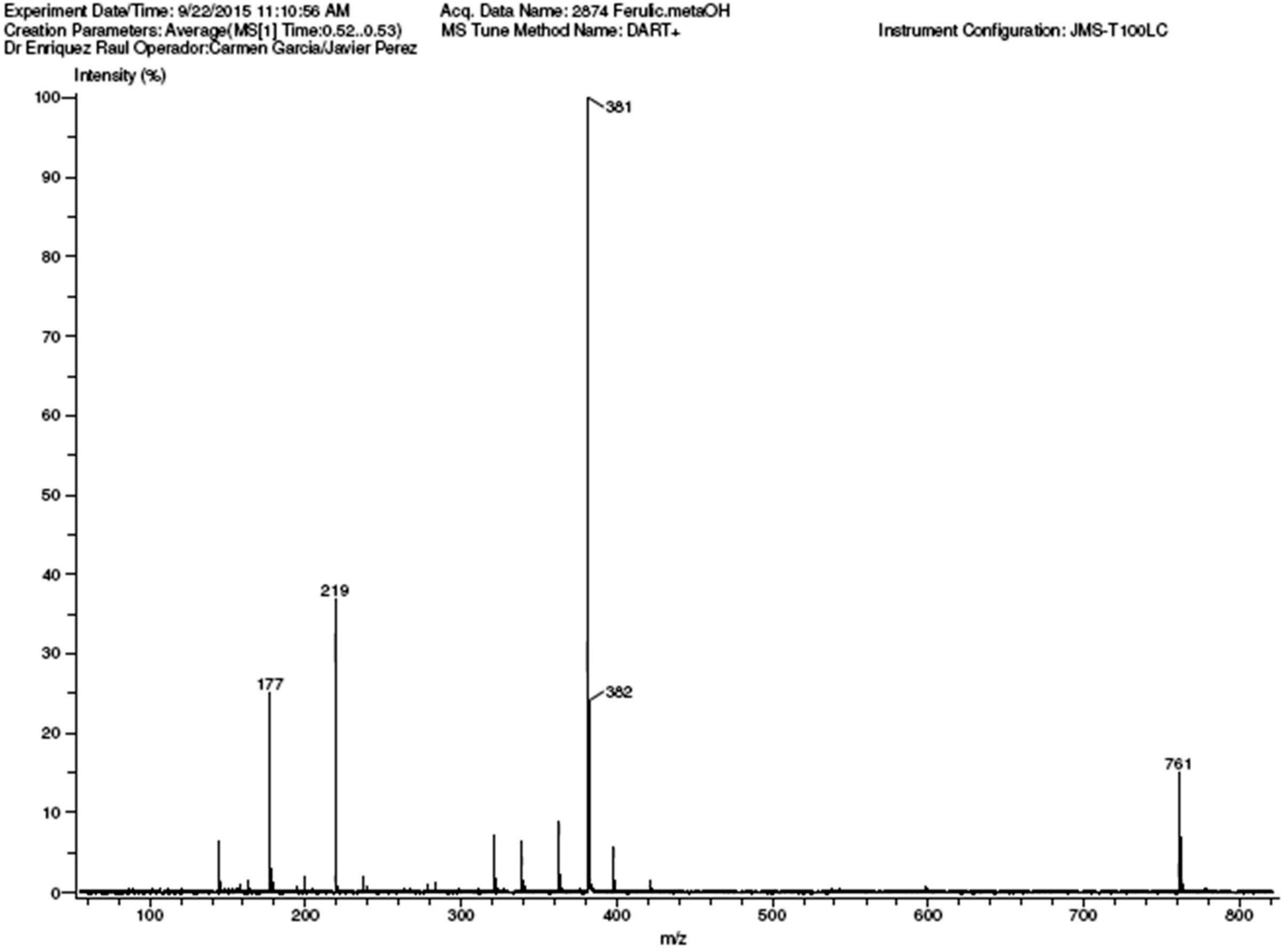

MS *Retro*-Curcuminoid **12**

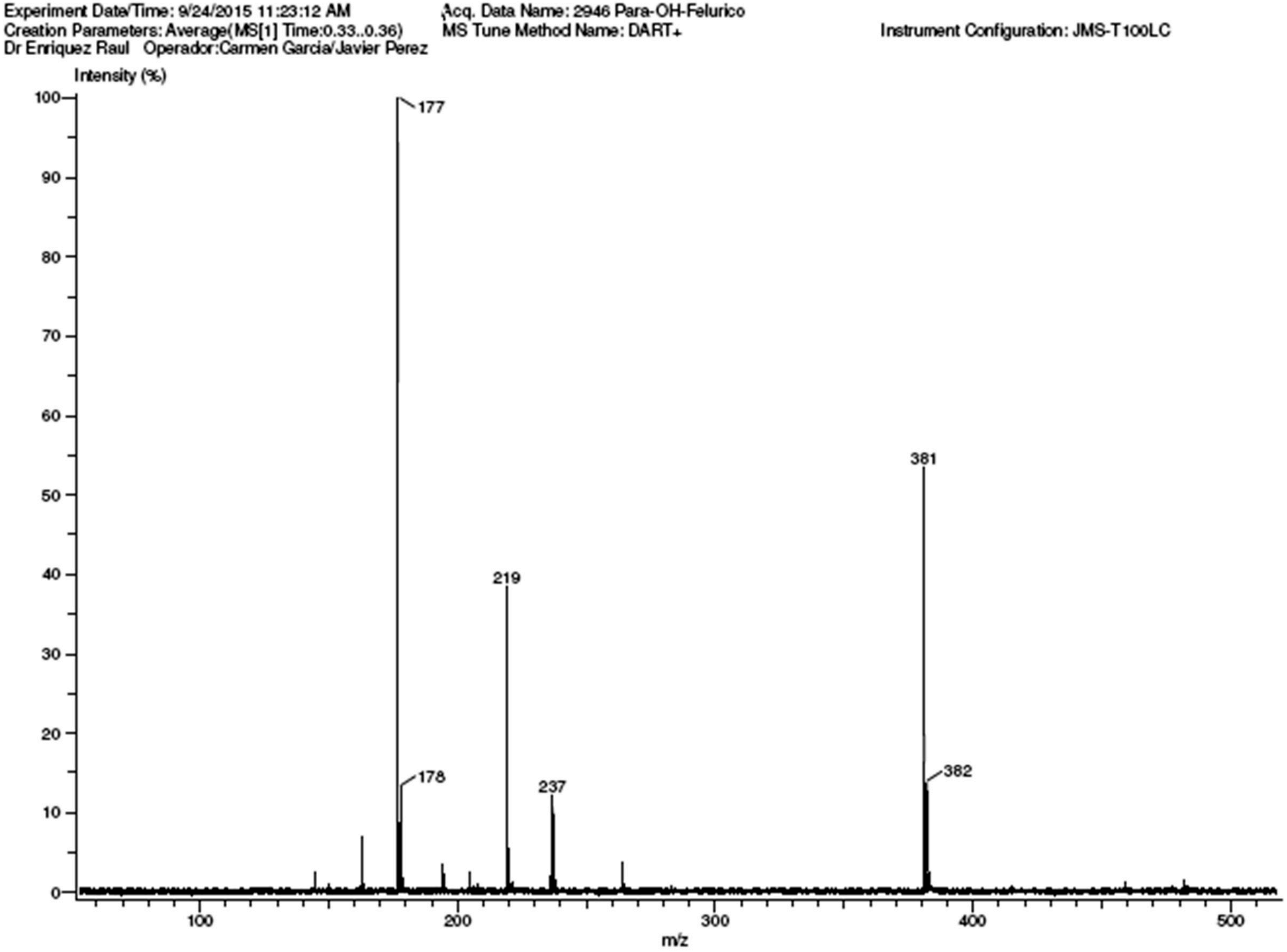

MS *Retro*-Curcuminoid **13**

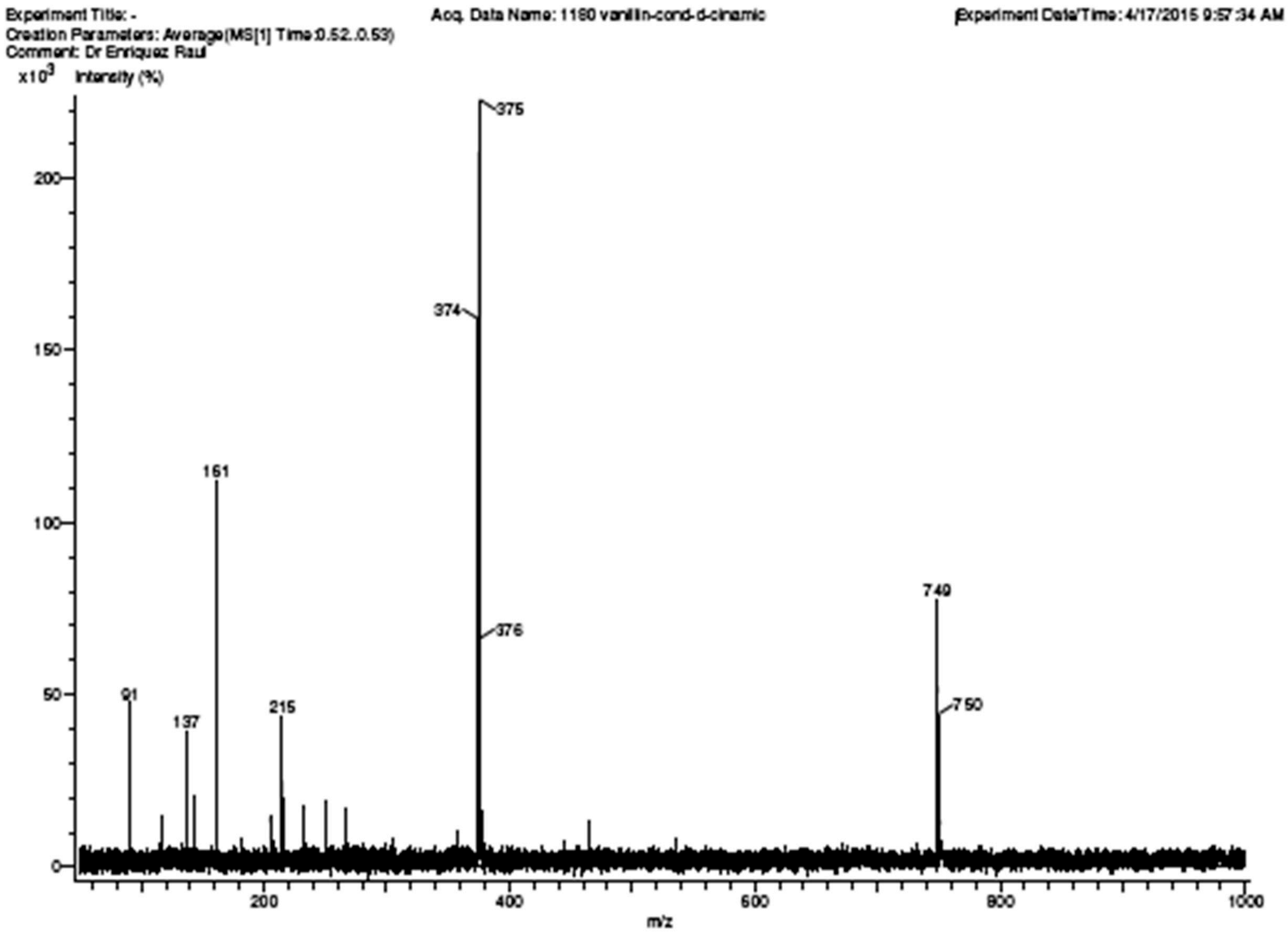

MS *Retro*-Curcuminoid **14**

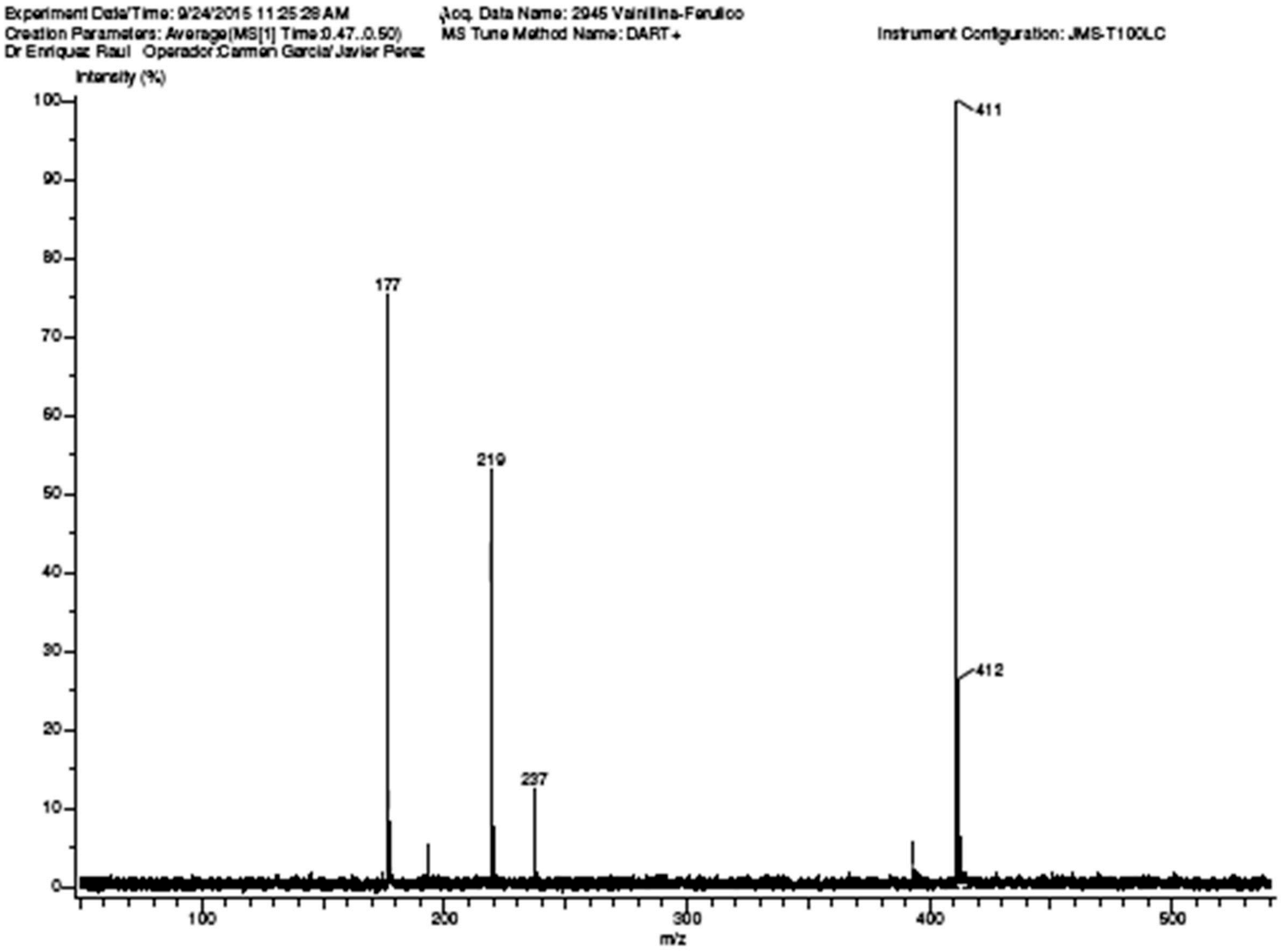

NMR *Retro*-Curcuminoid **7**

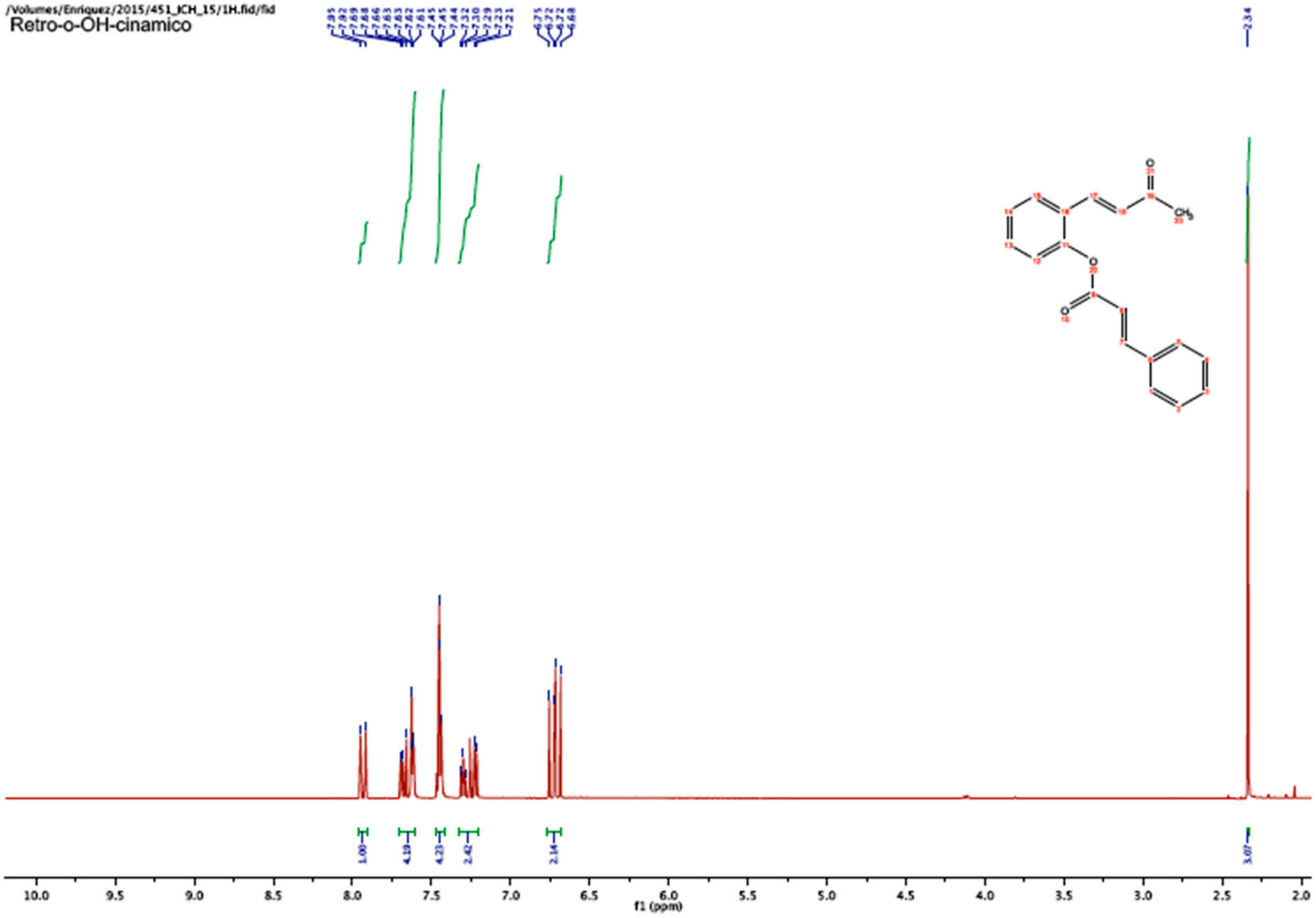

NMR *Retro*-Curcuminoid **7**

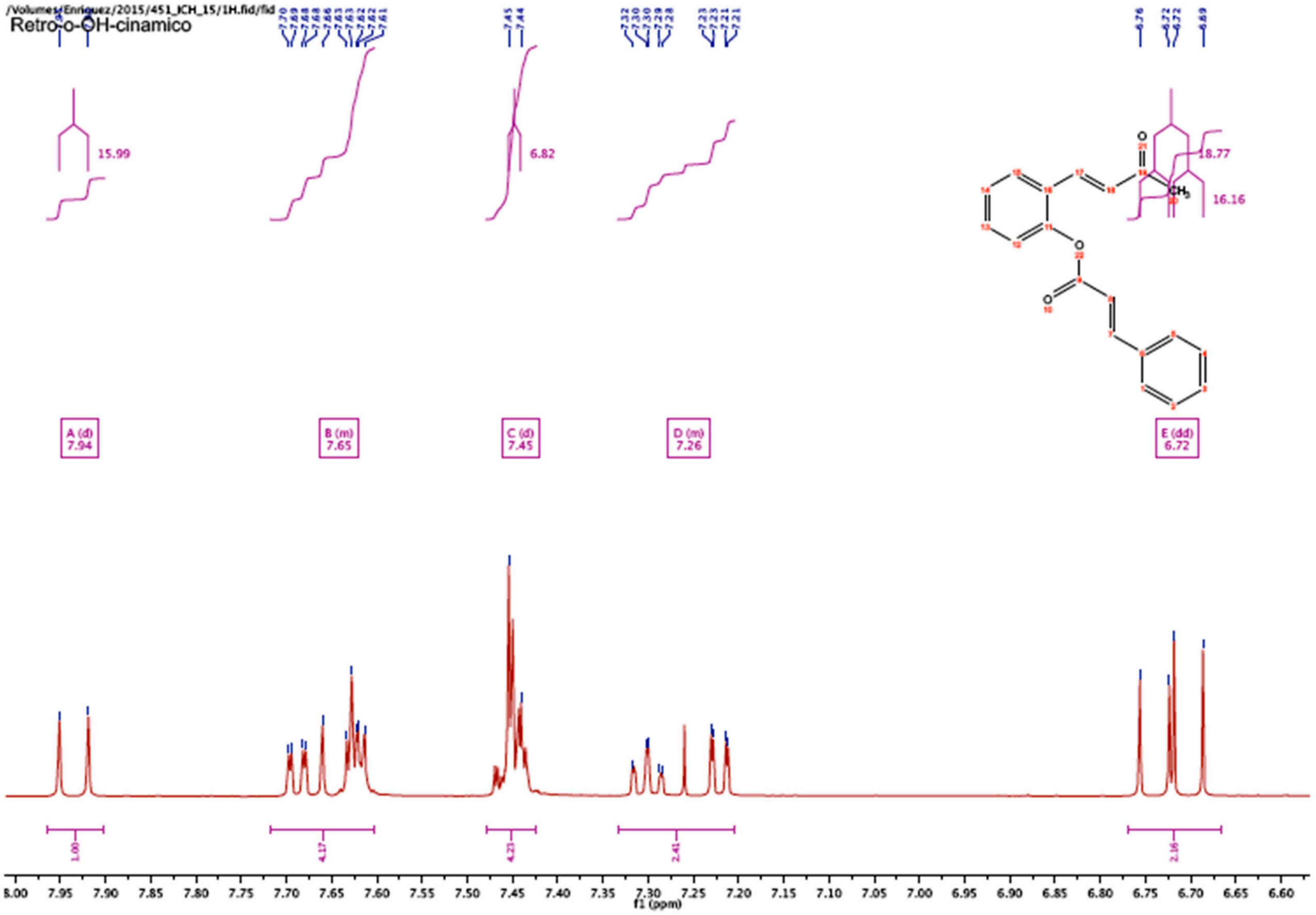

NMR *Retro*-Curcuminoid **7**

Retro-o-OH-cinamico

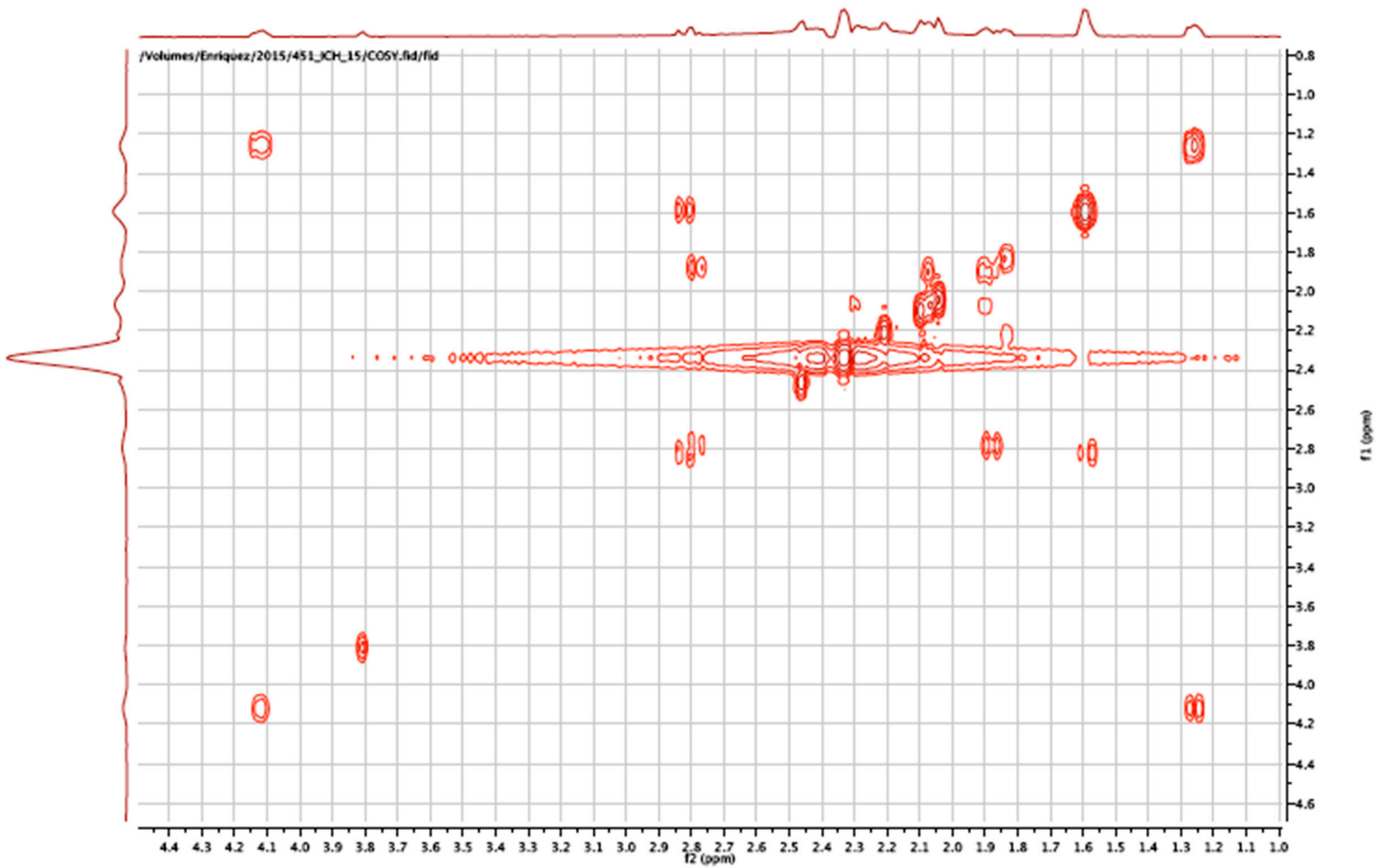

NMR *Retro*-Curcuminoid **7**

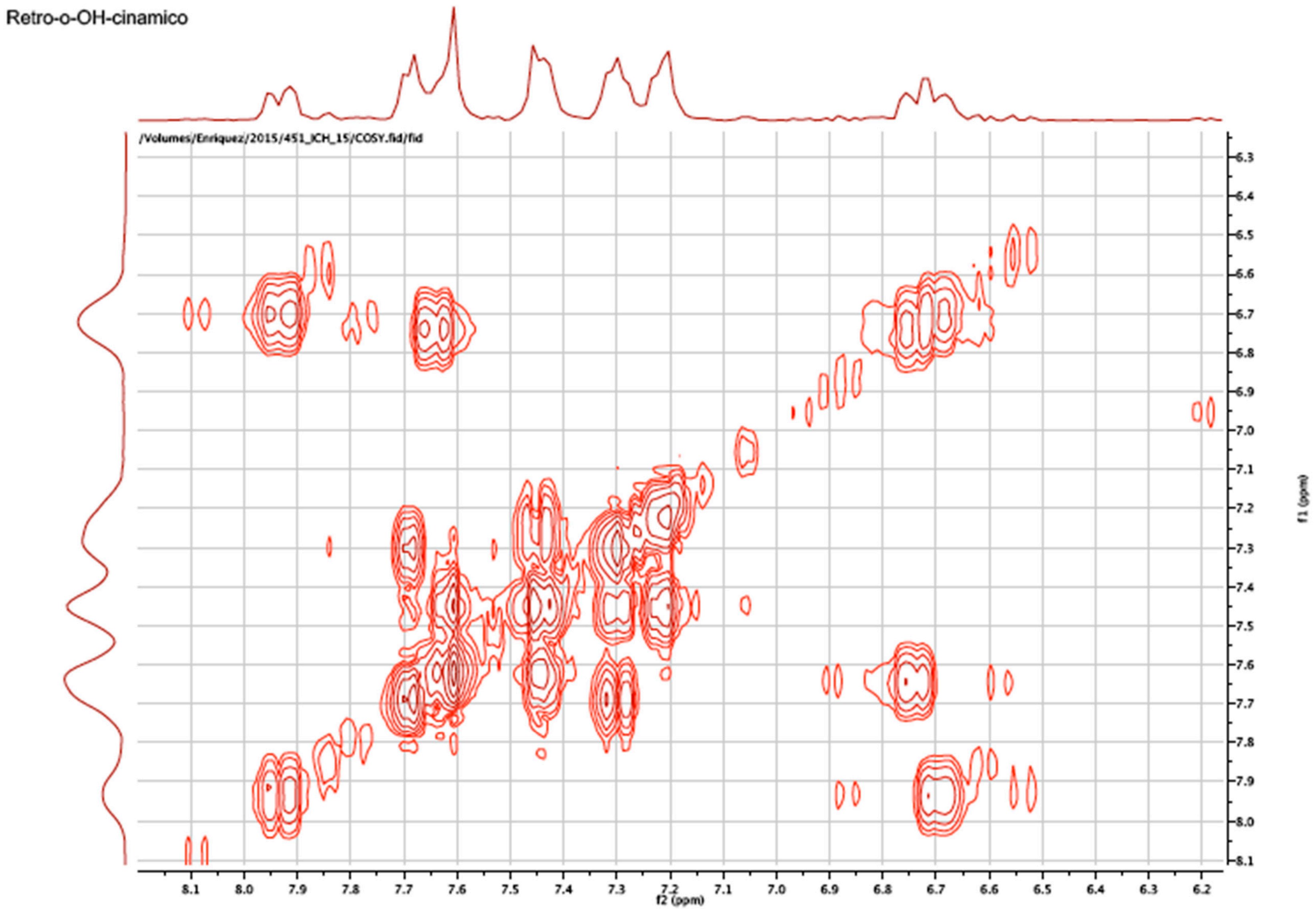

NMR *Retro*-Curcuminoid **7**

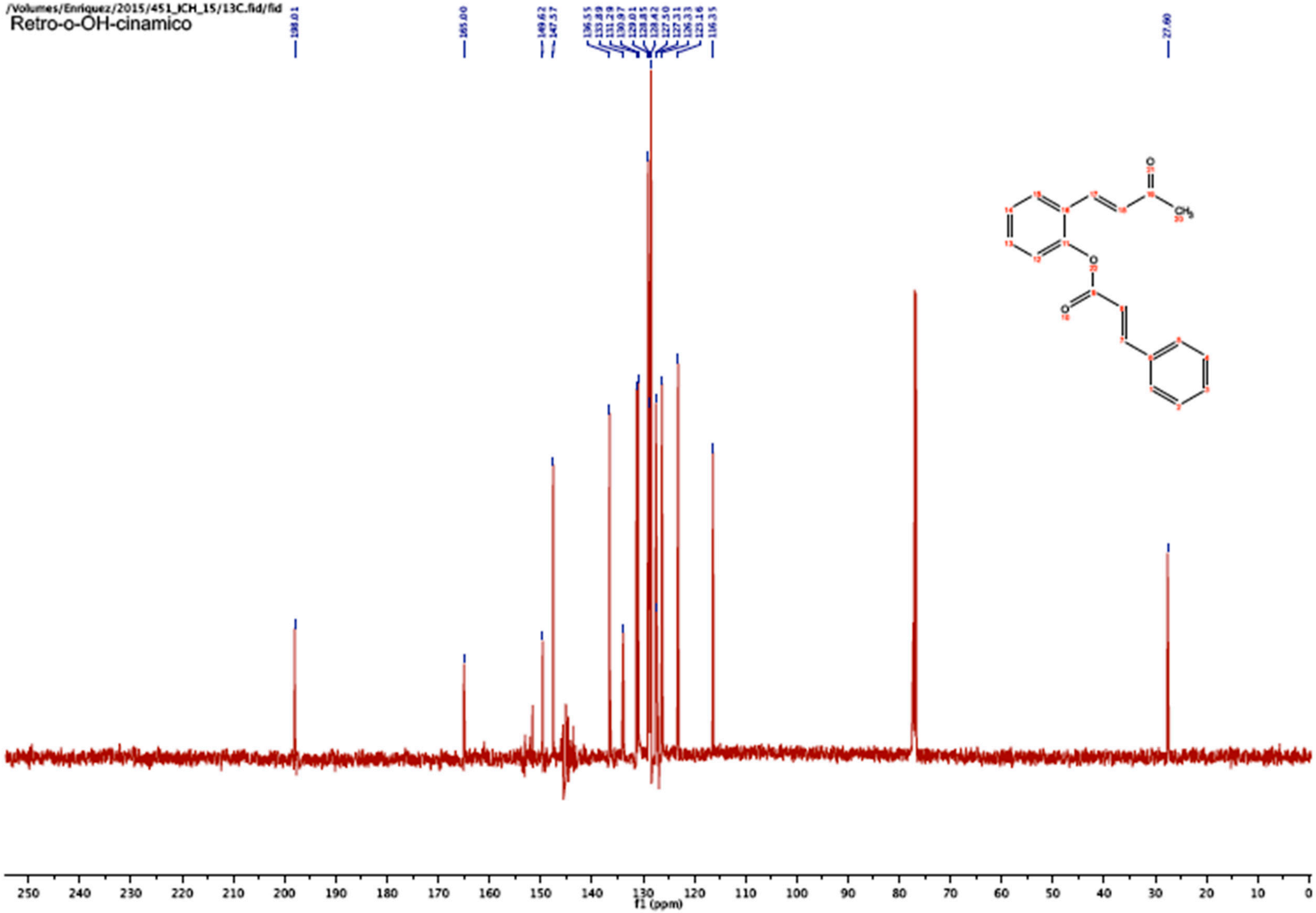

NMR *Retro*-Curcuminoid **7**

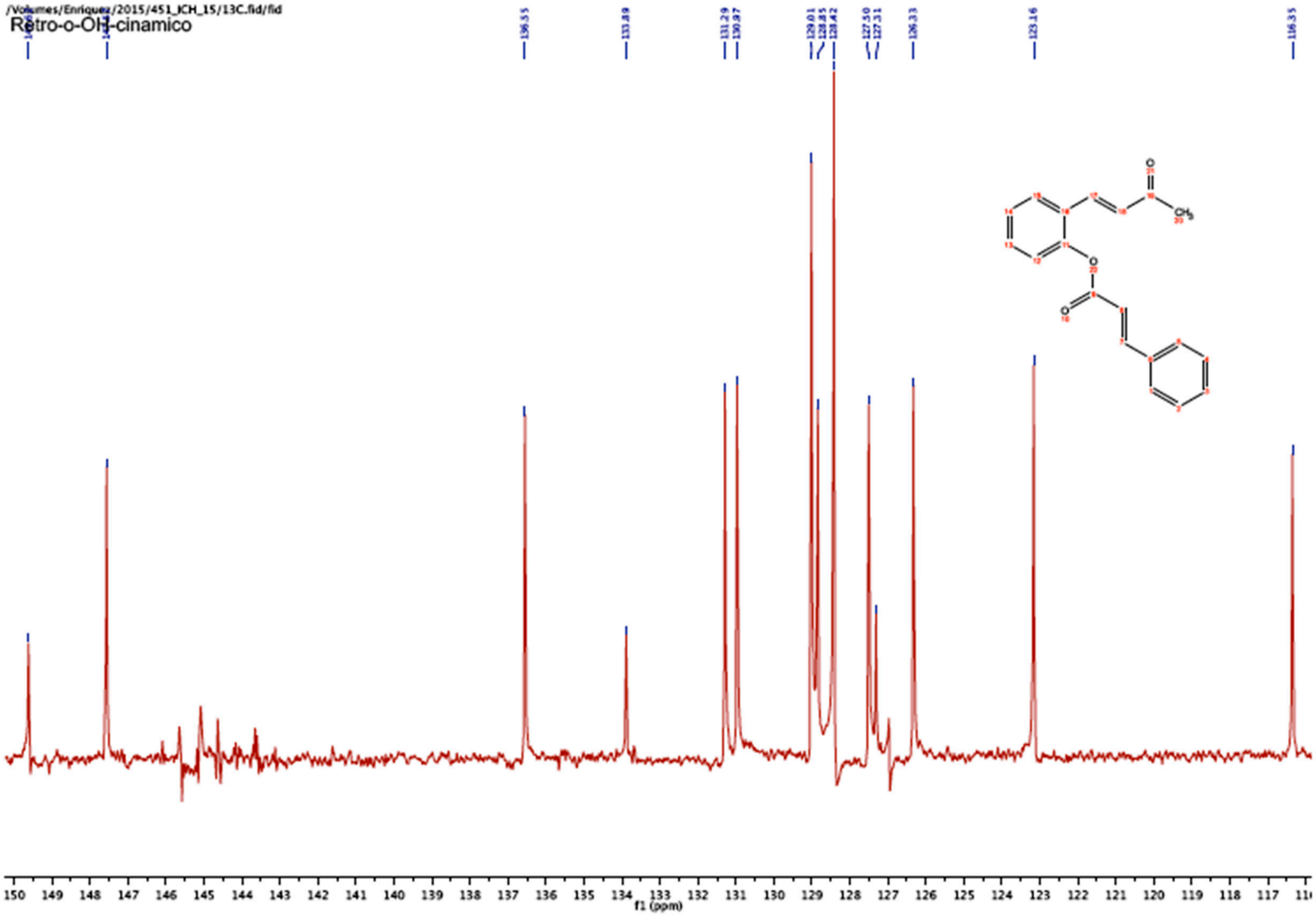

NMR *Retro*-Curcuminoid **7**

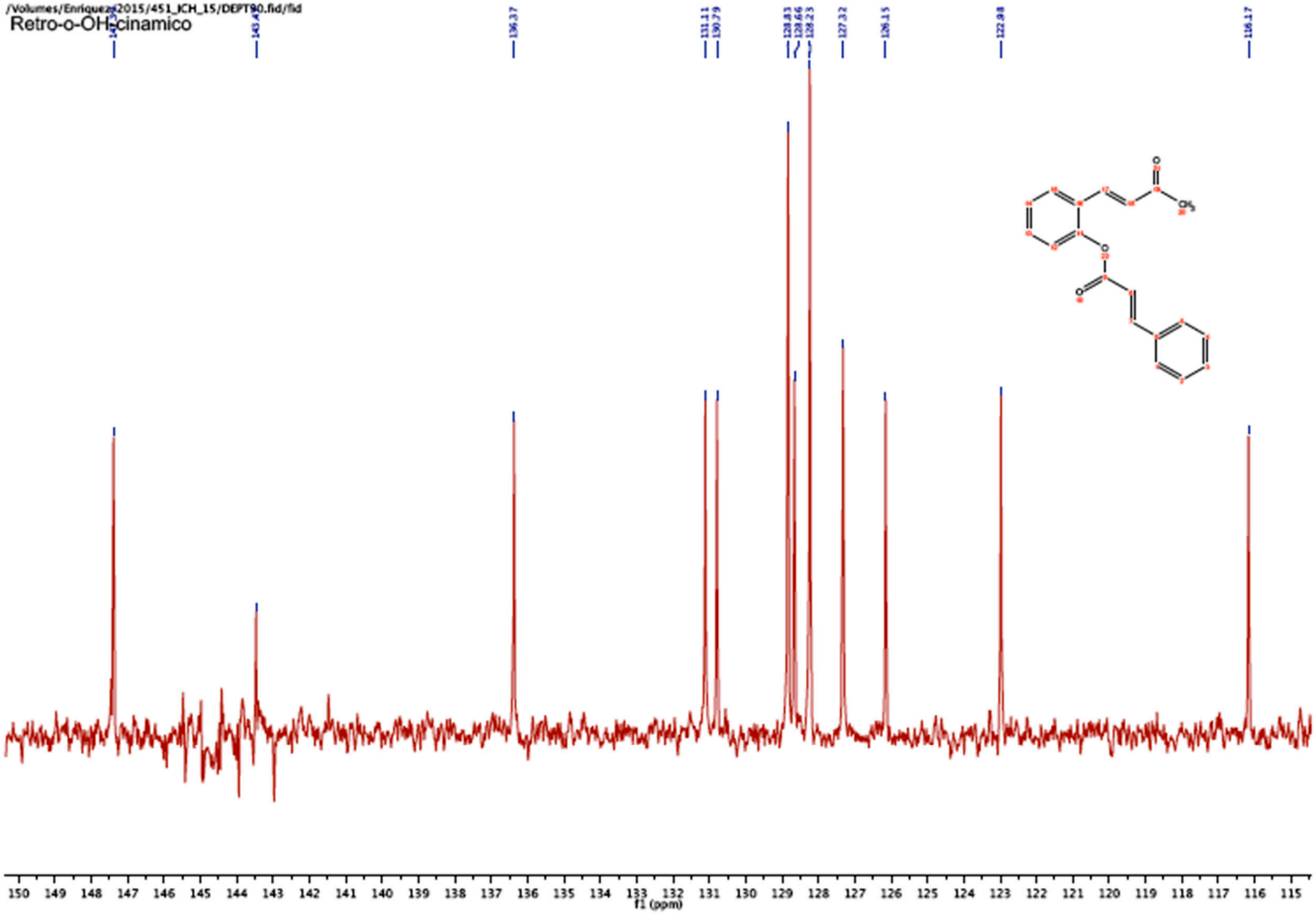

NMR *Retro*-Curcuminoid **7**

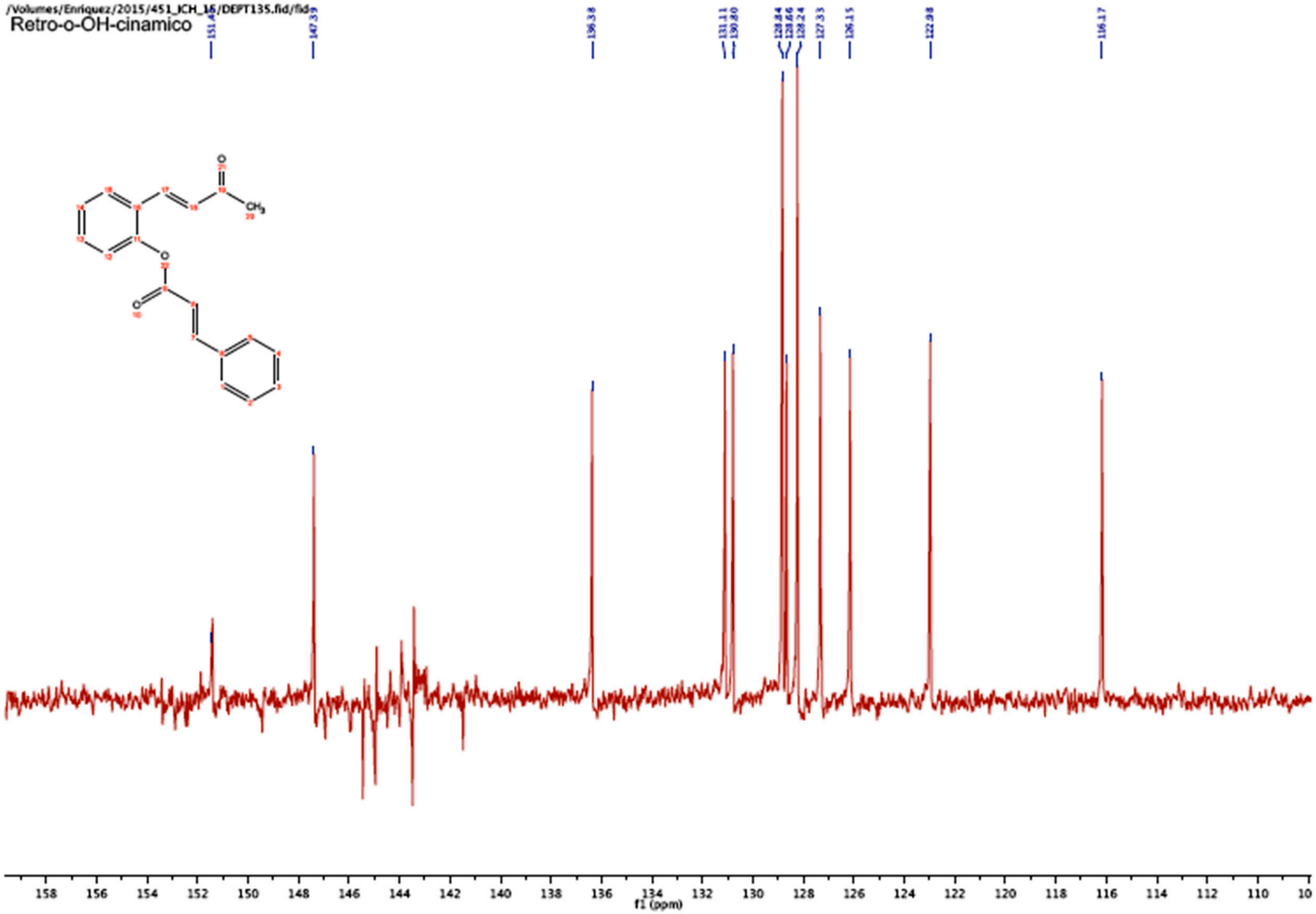

NMR *Retro*-Curcuminoid **7**

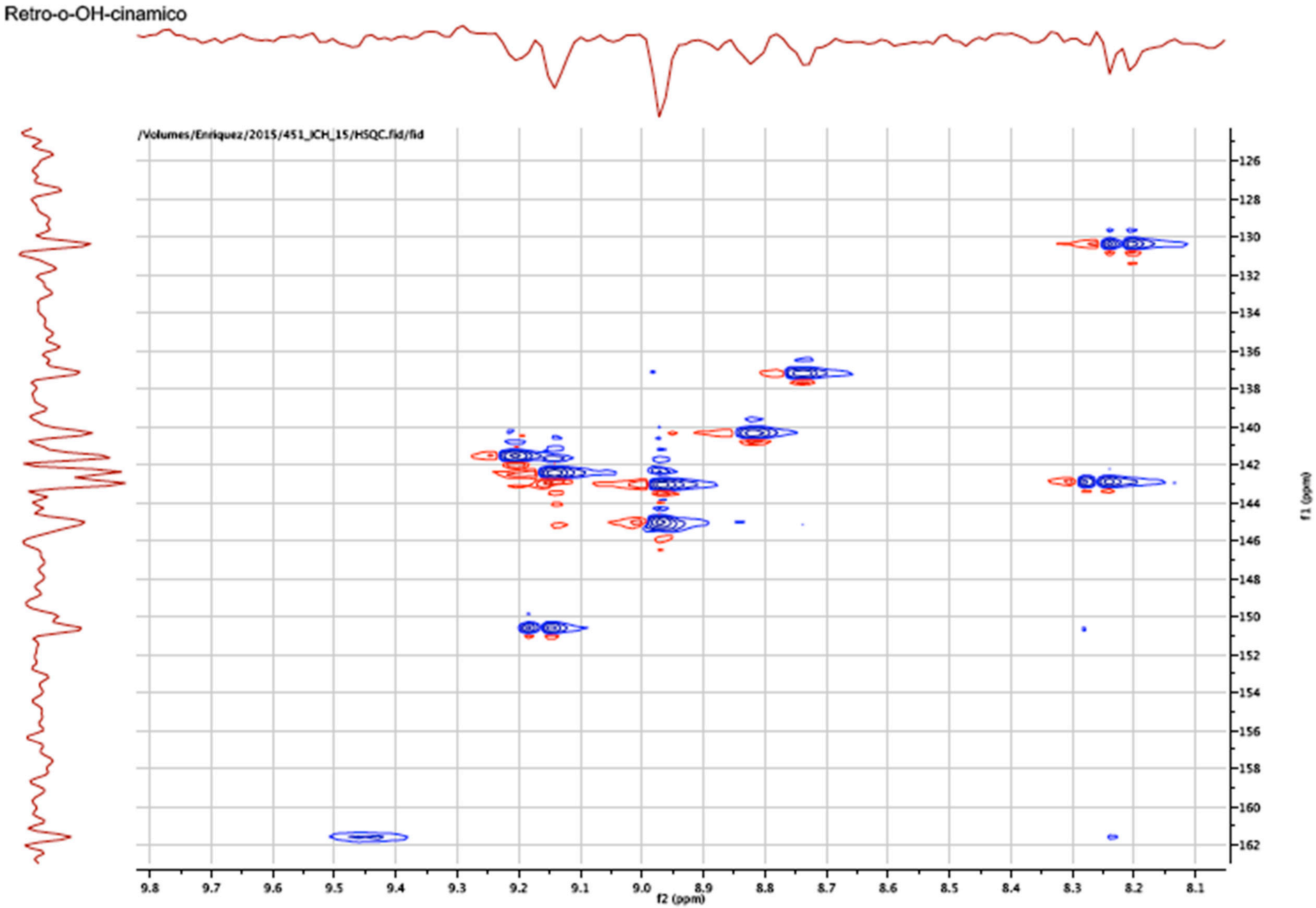

NMR *Retro*-Curcuminoid **7**

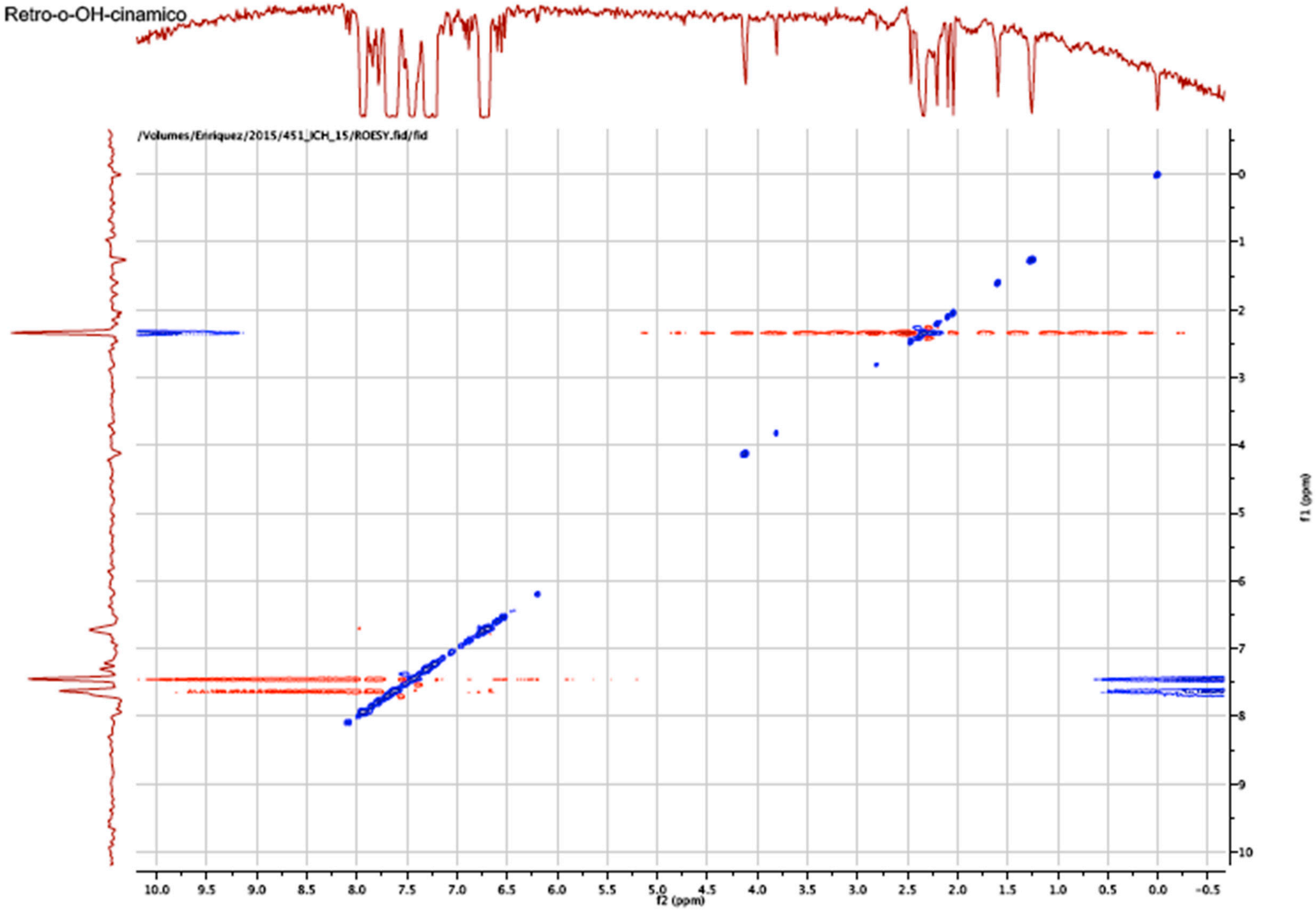

NMR *Retro*-Curcuminoid **8**

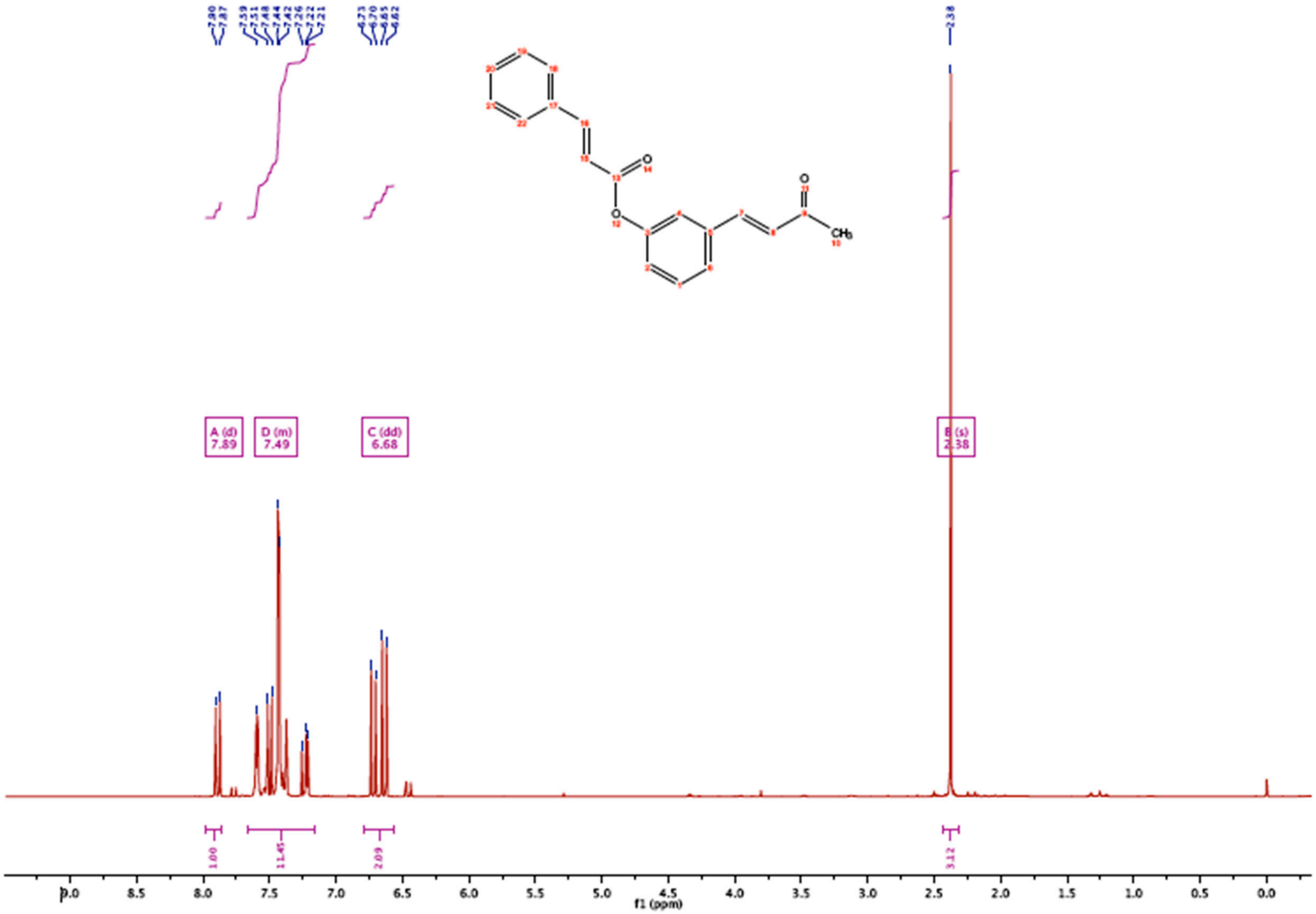

NMR *Retro*-Curcuminoid **8**

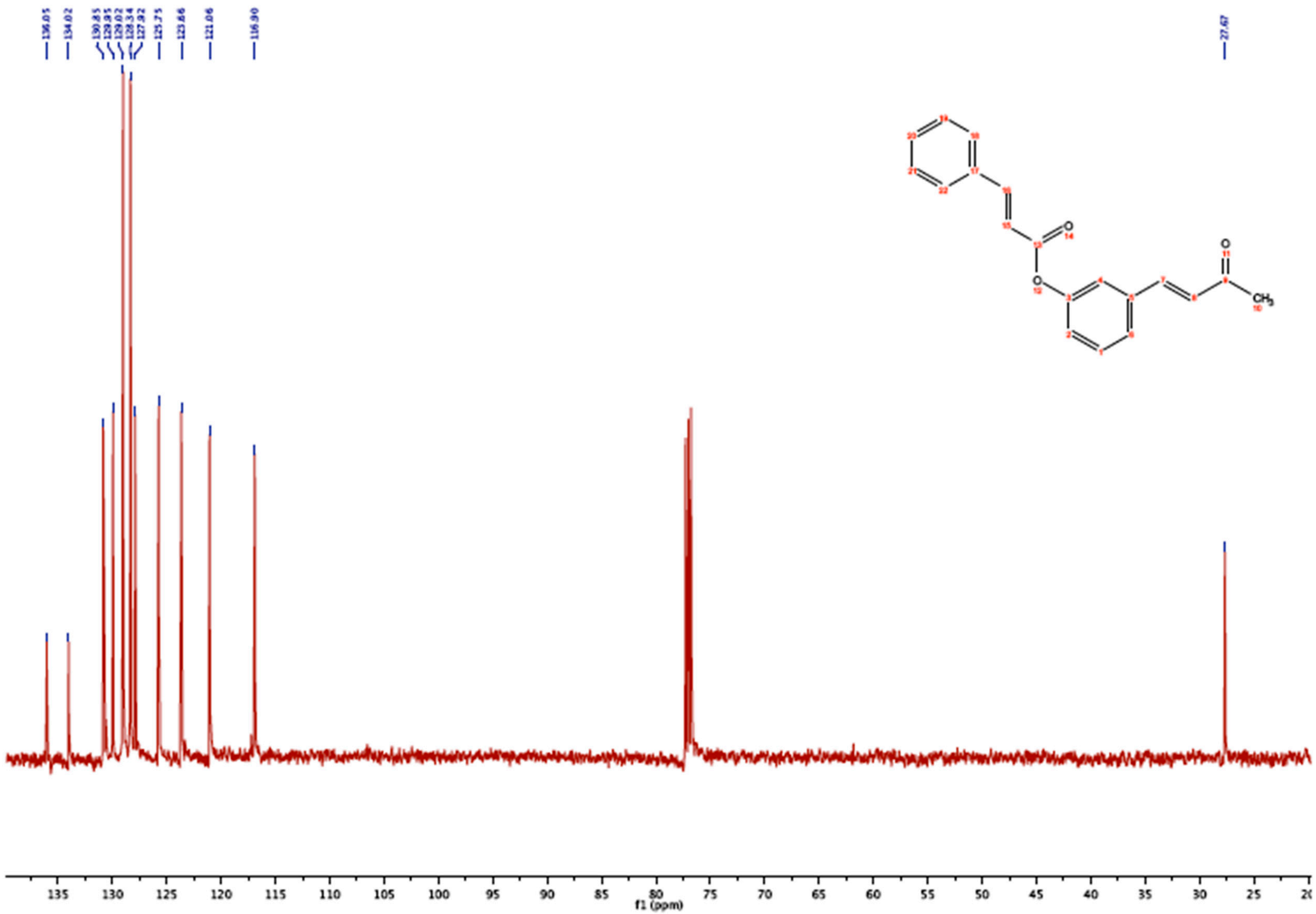

NMR *Retro*-Curcuminoid **8**

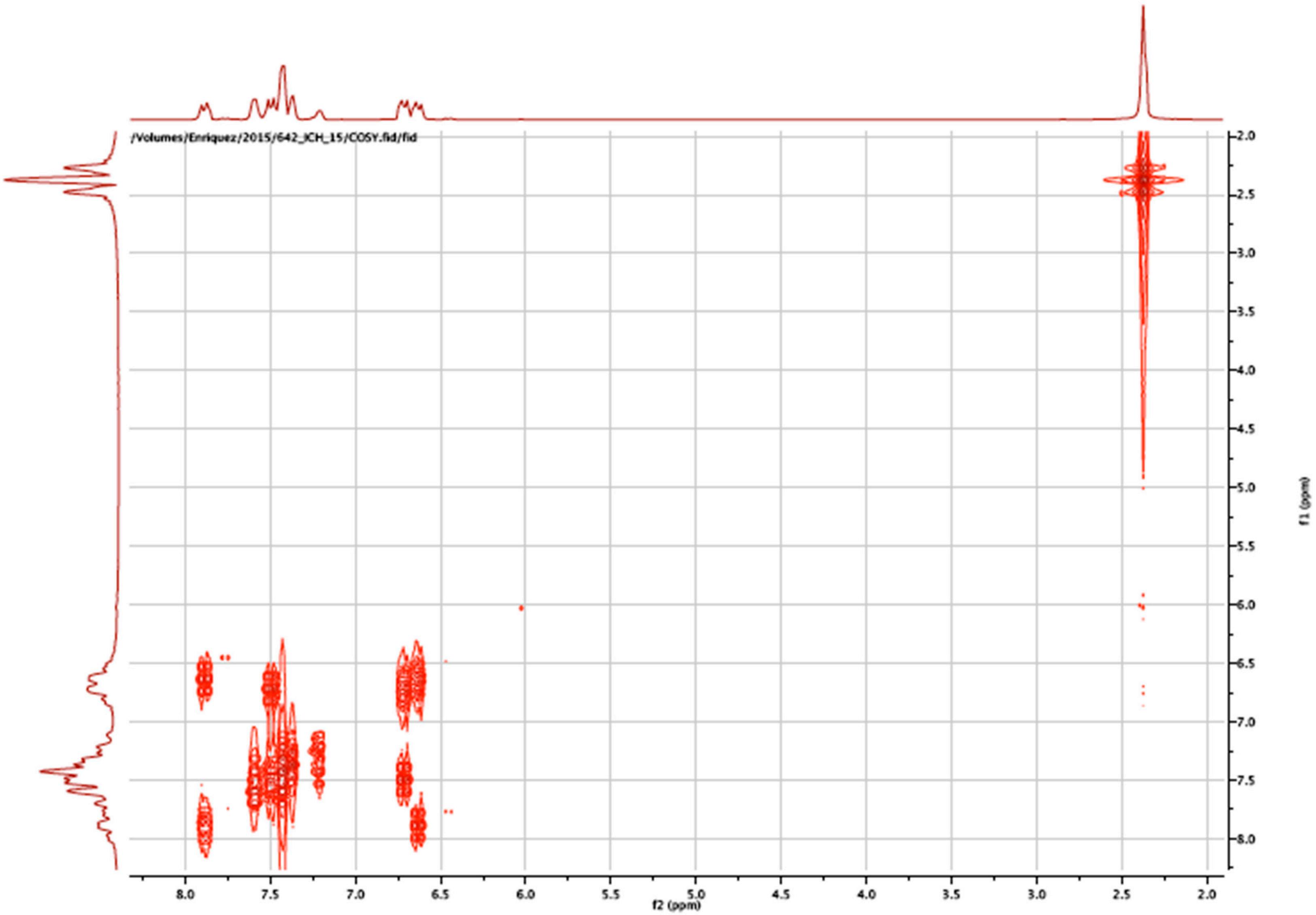

NMR *Retro*-Curcuminoid **8**

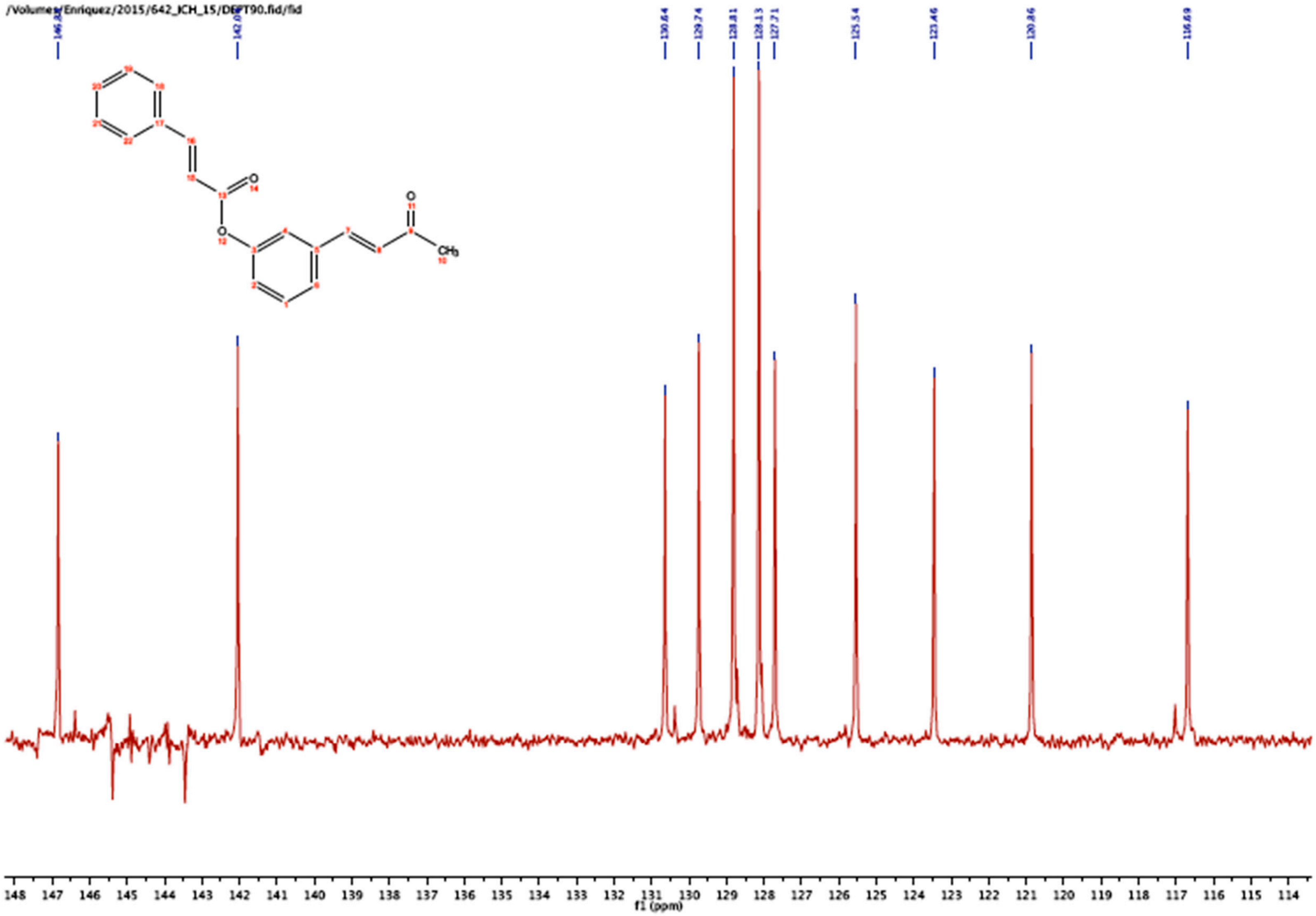

NMR *Retro*-Curcuminoid **8**

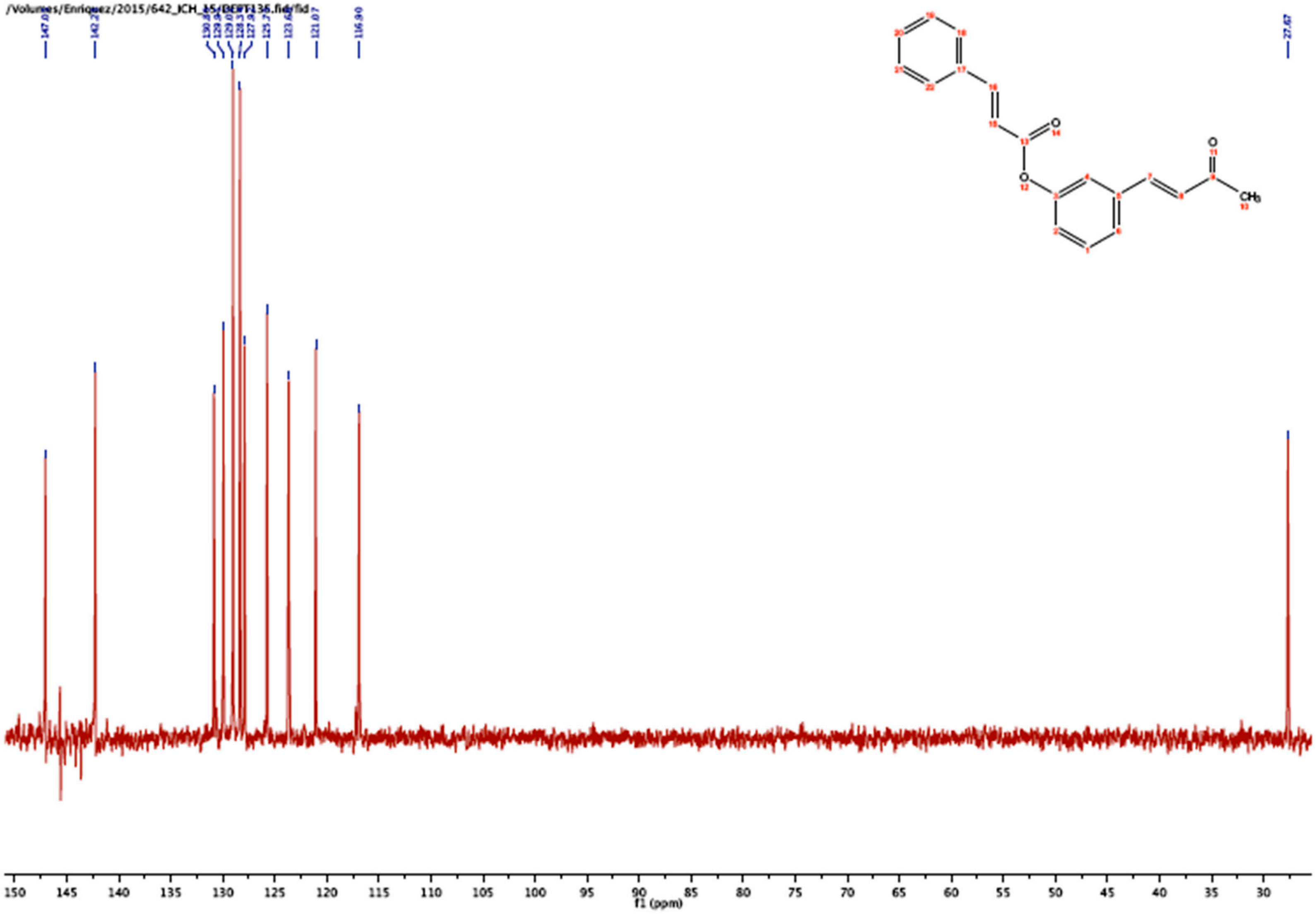

NMR *Retro*-Curcuminoid **8**

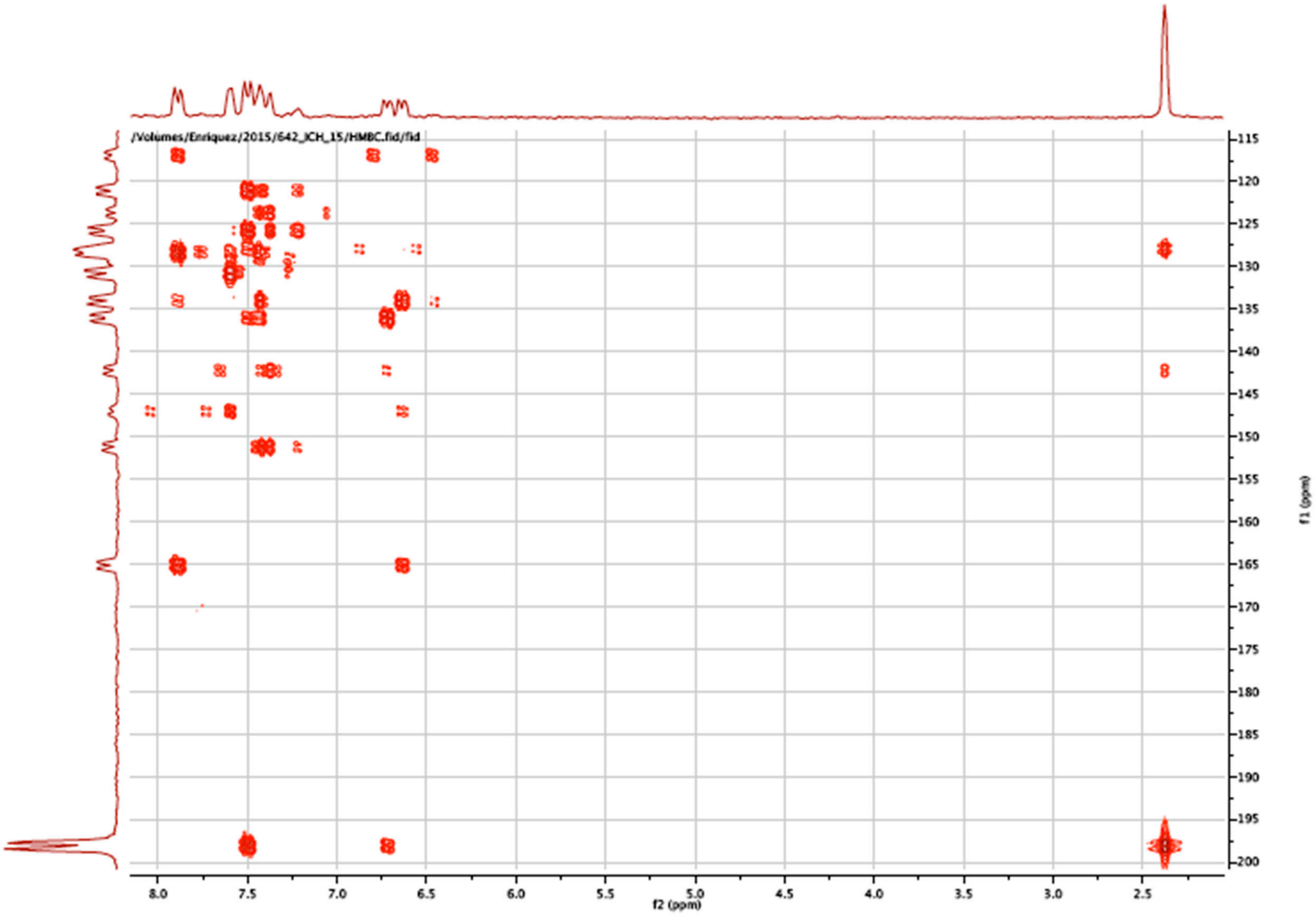

NMR *Retro*-Curcuminoid **8**

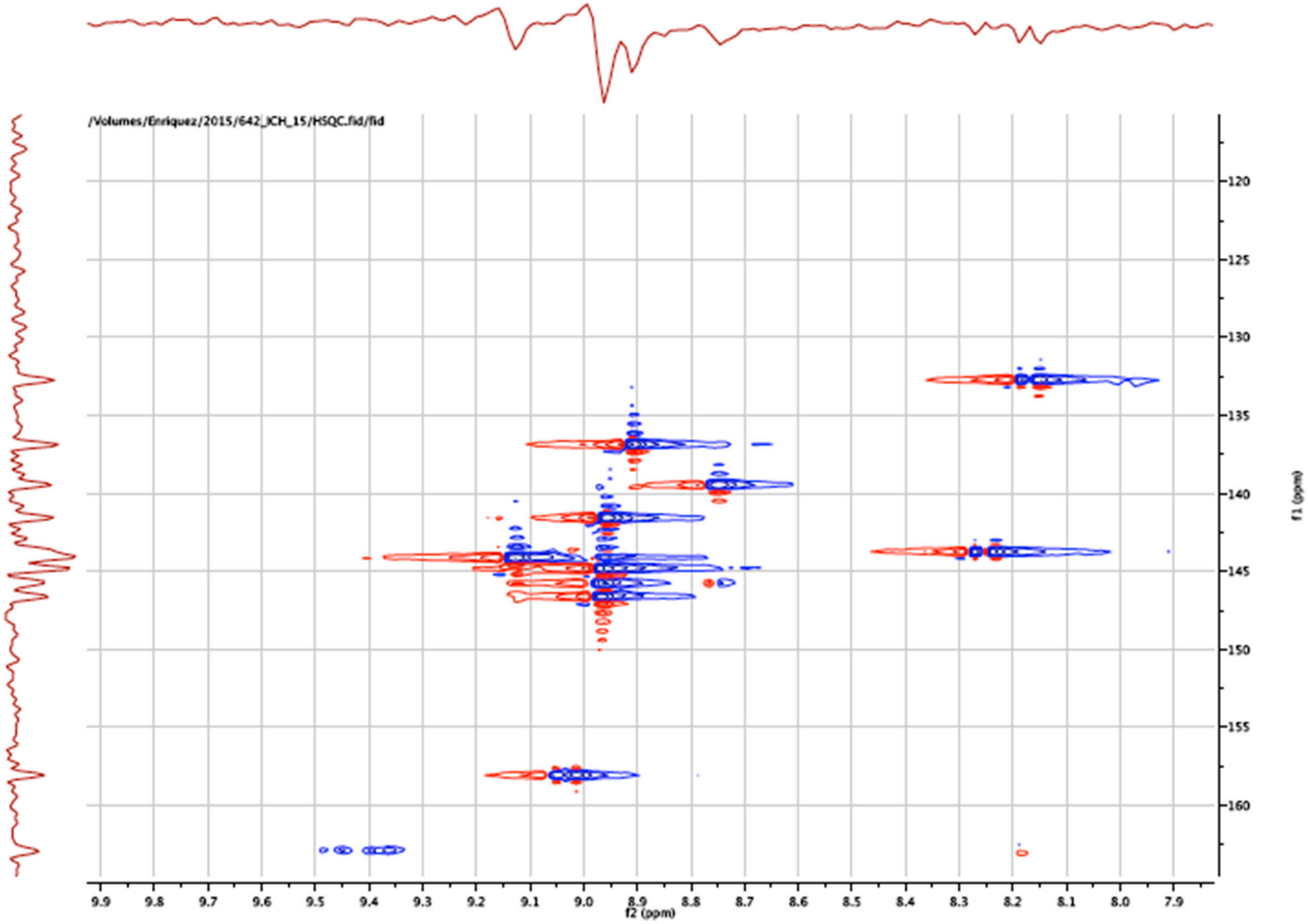

NMR *Retro*-Curcuminoid **8**

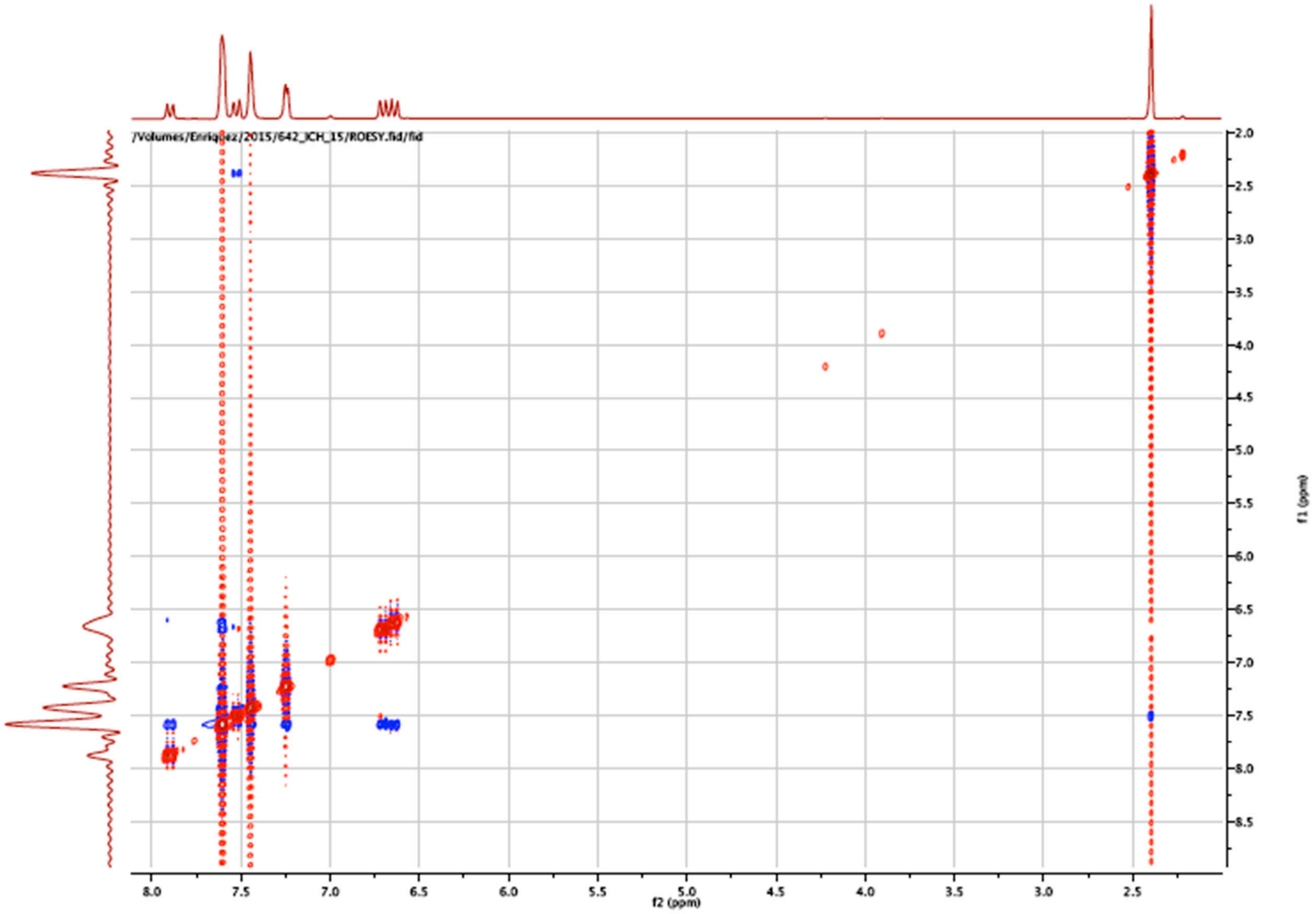

NMR *Retro*-Curcuminoid **9**

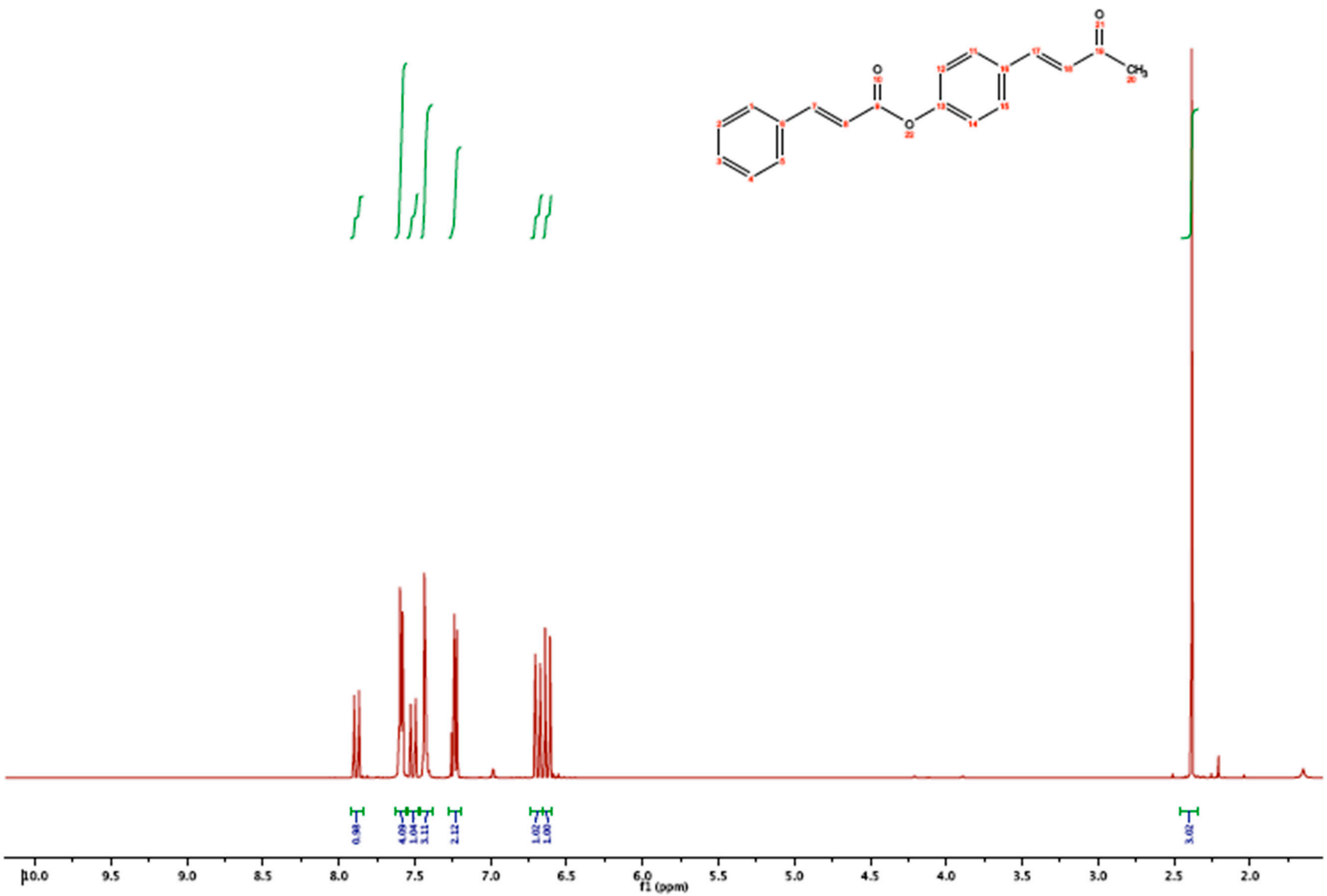

NMR *Retro*-Curcuminoid **9**

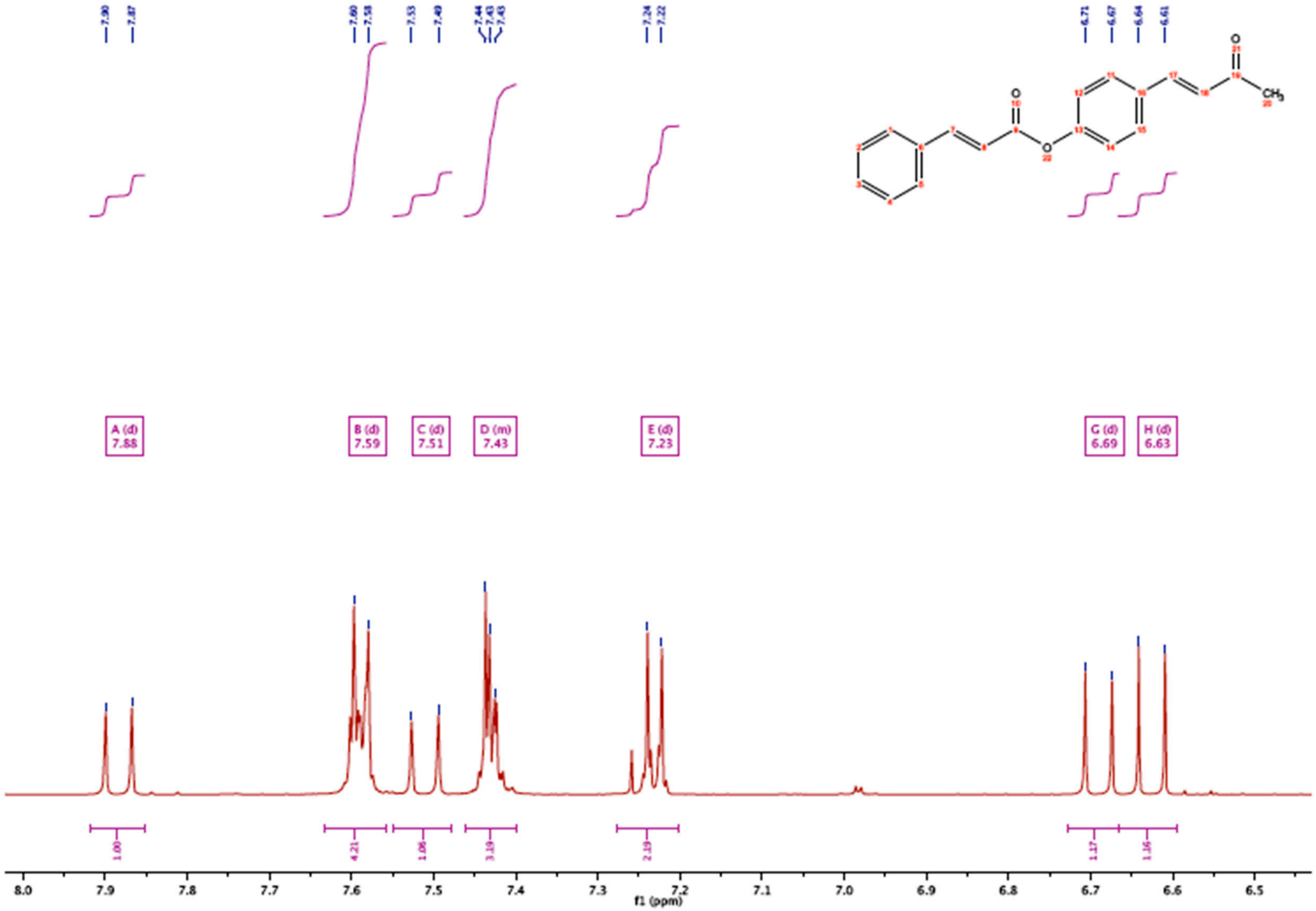

NMR *Retro*-Curcuminoid **9**

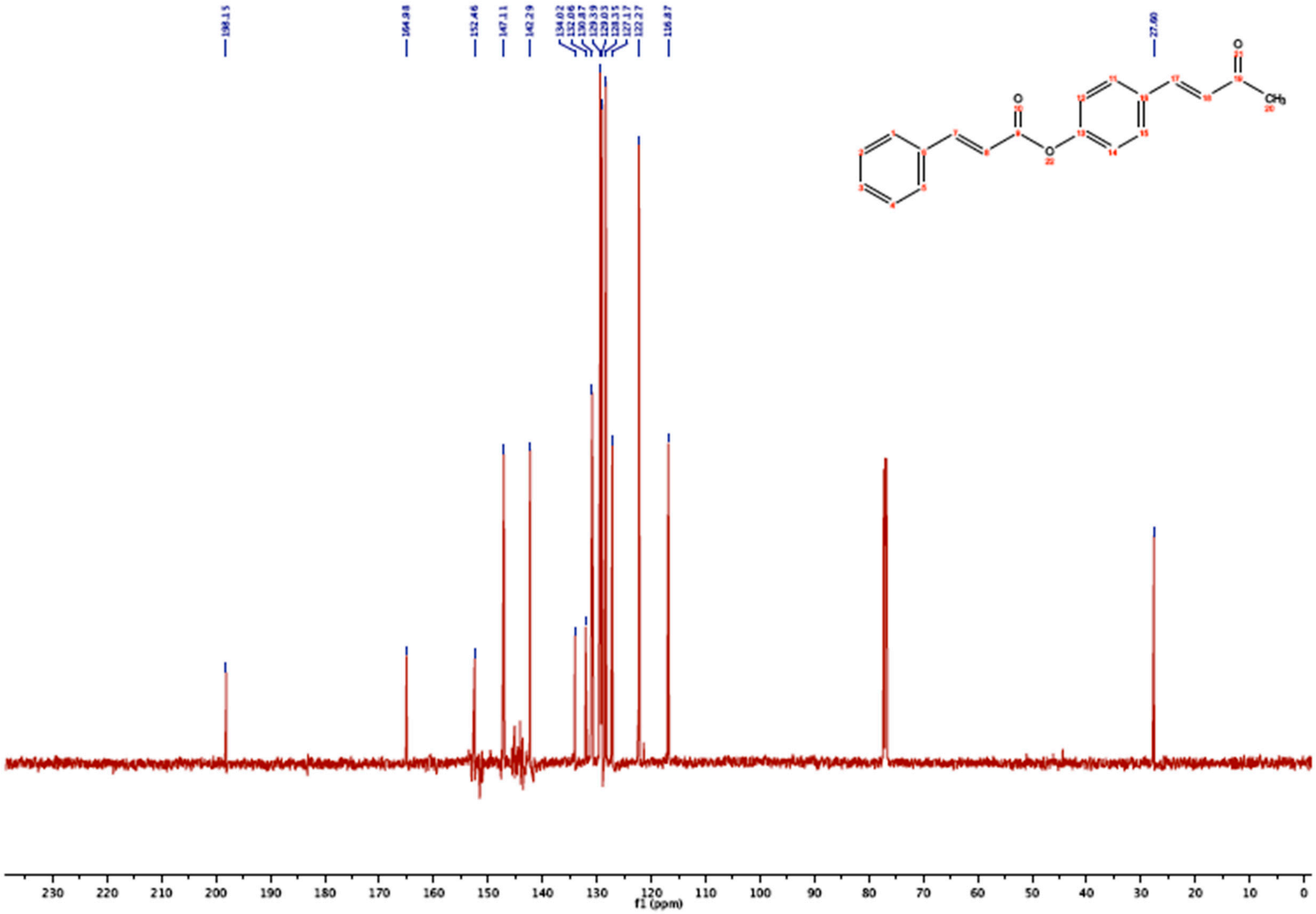

NMR *Retro*-Curcuminoid **9**

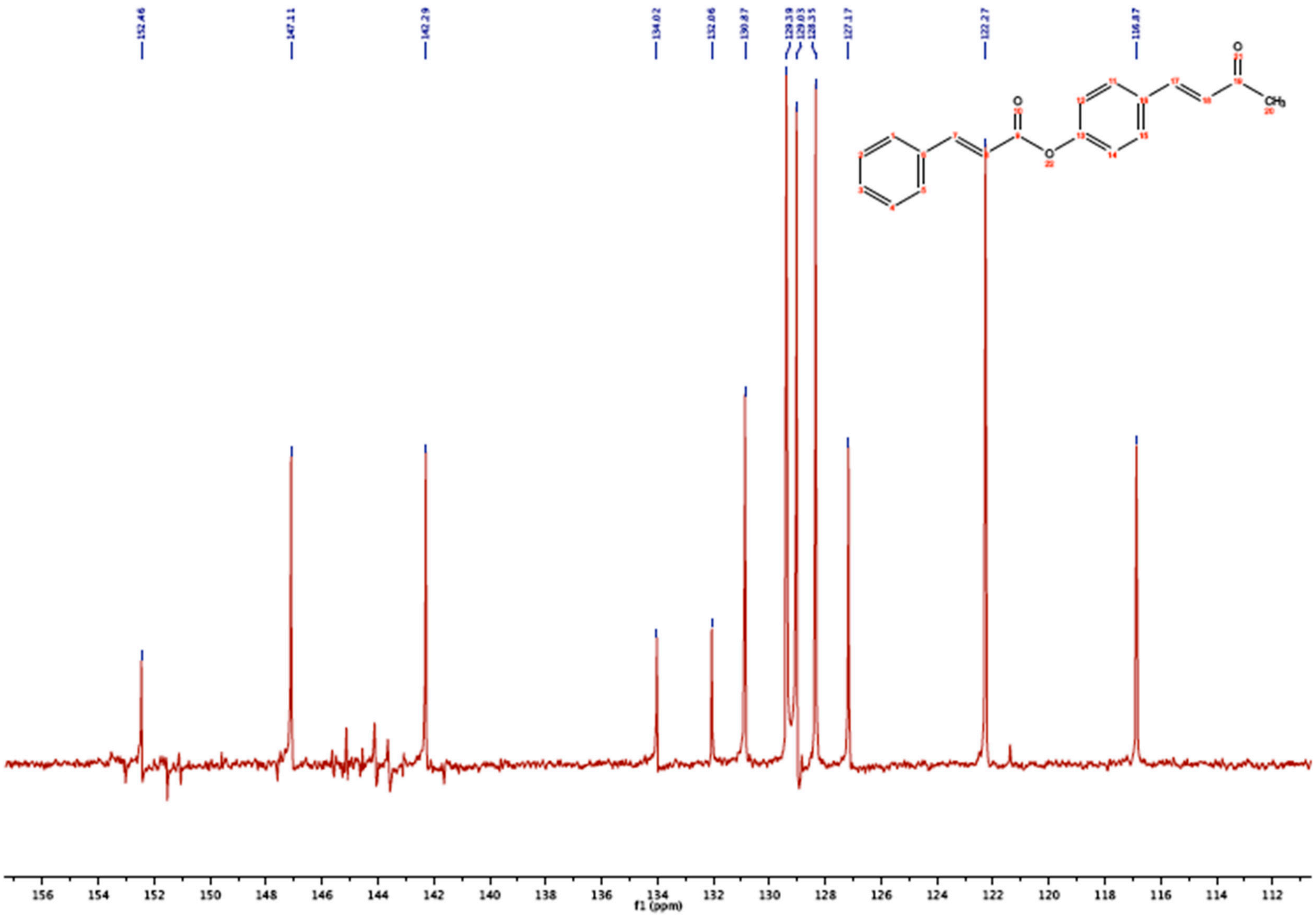

NMR *Retro*-Curcuminoid **9**

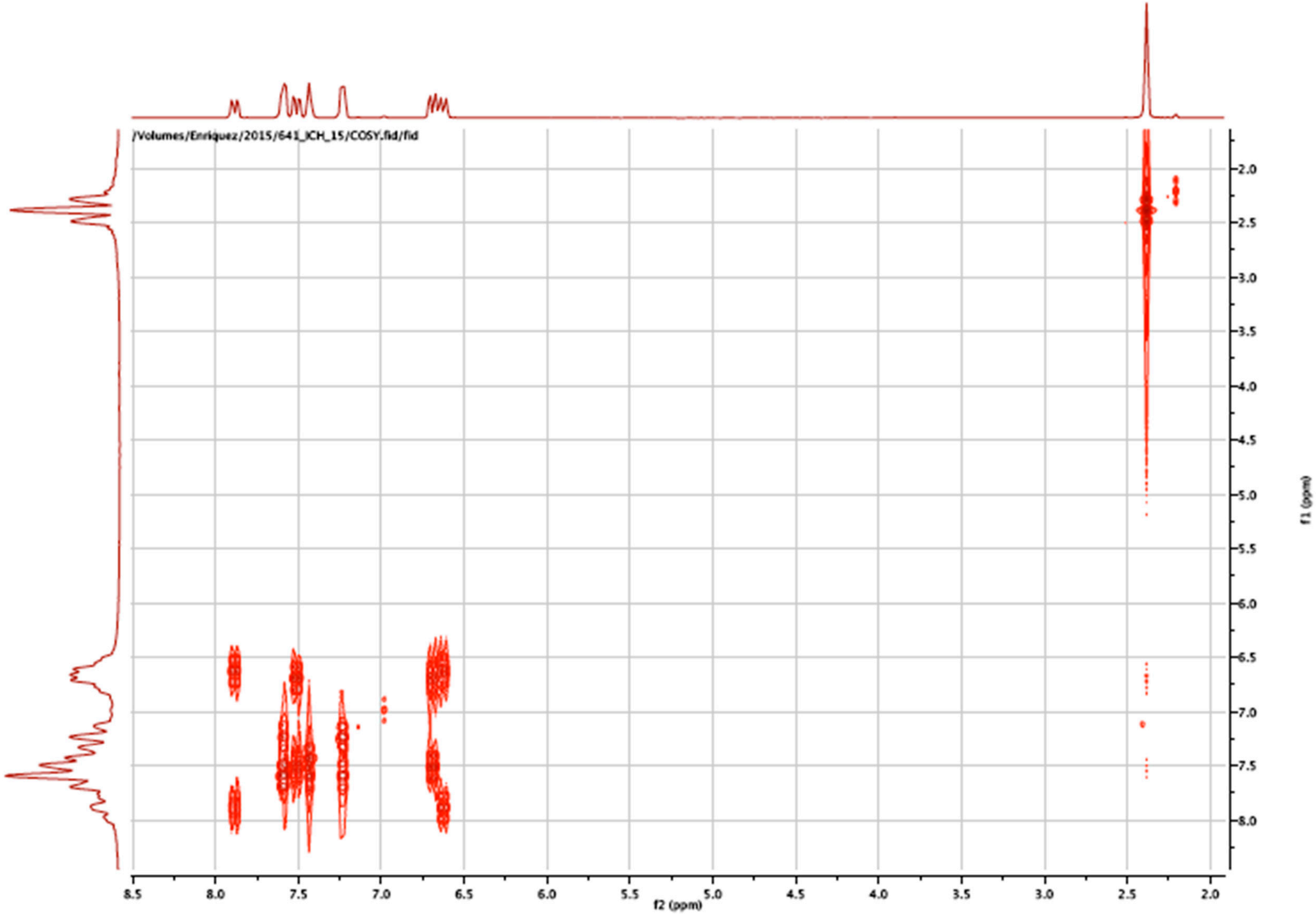

NMR *Retro*-Curcuminoid **9**

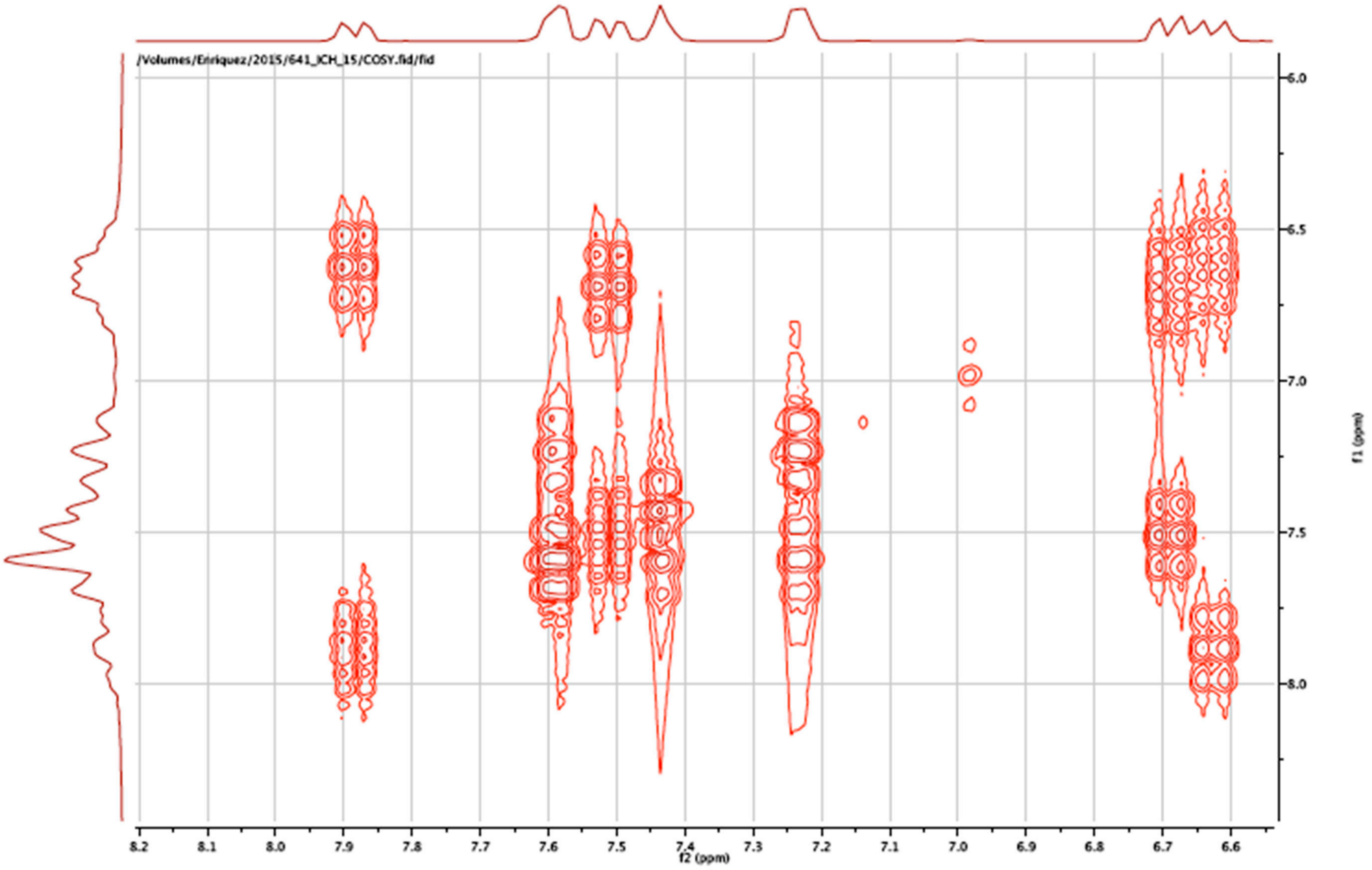

NMR *Retro*-Curcuminoid **9**

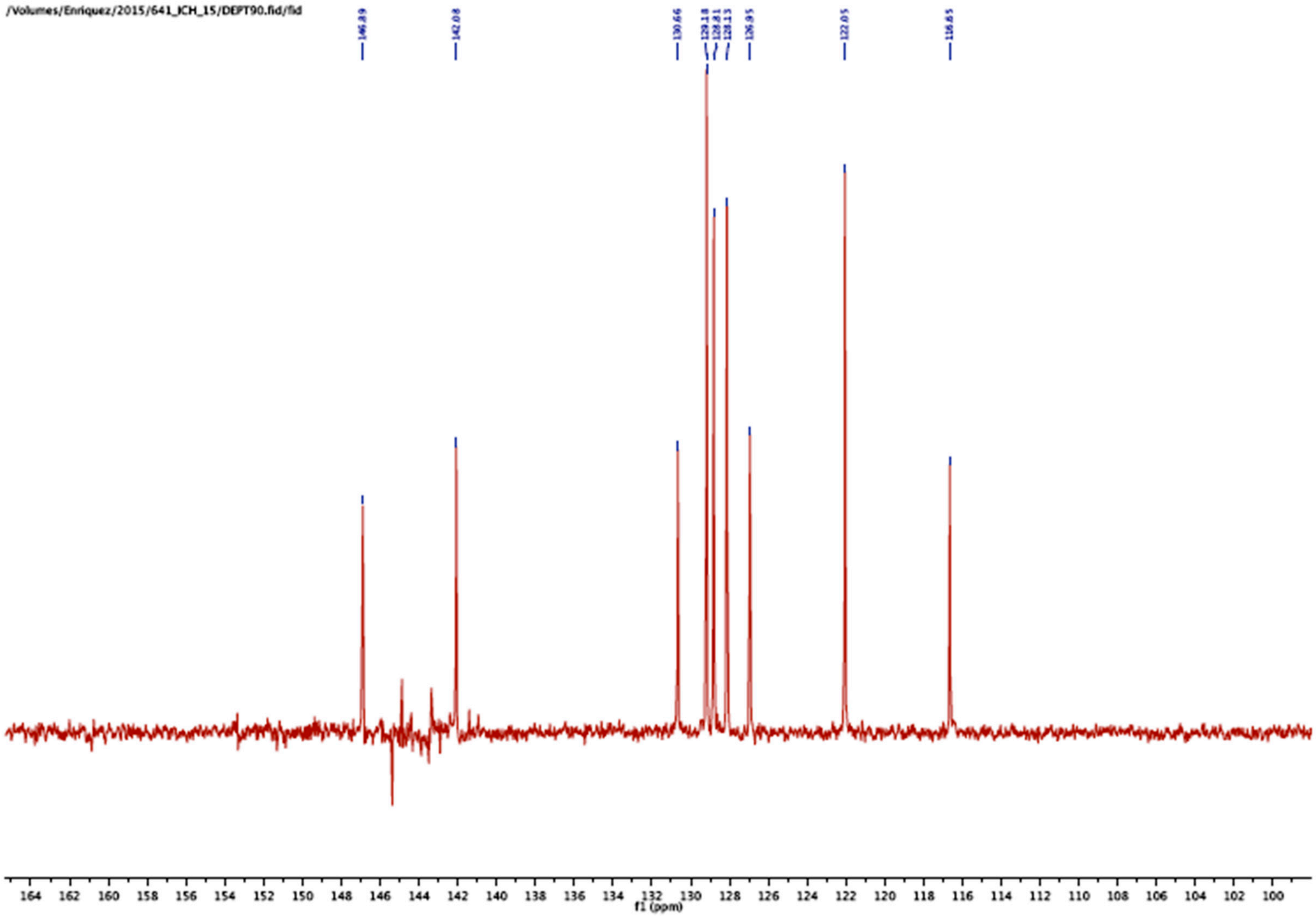

(**a**)

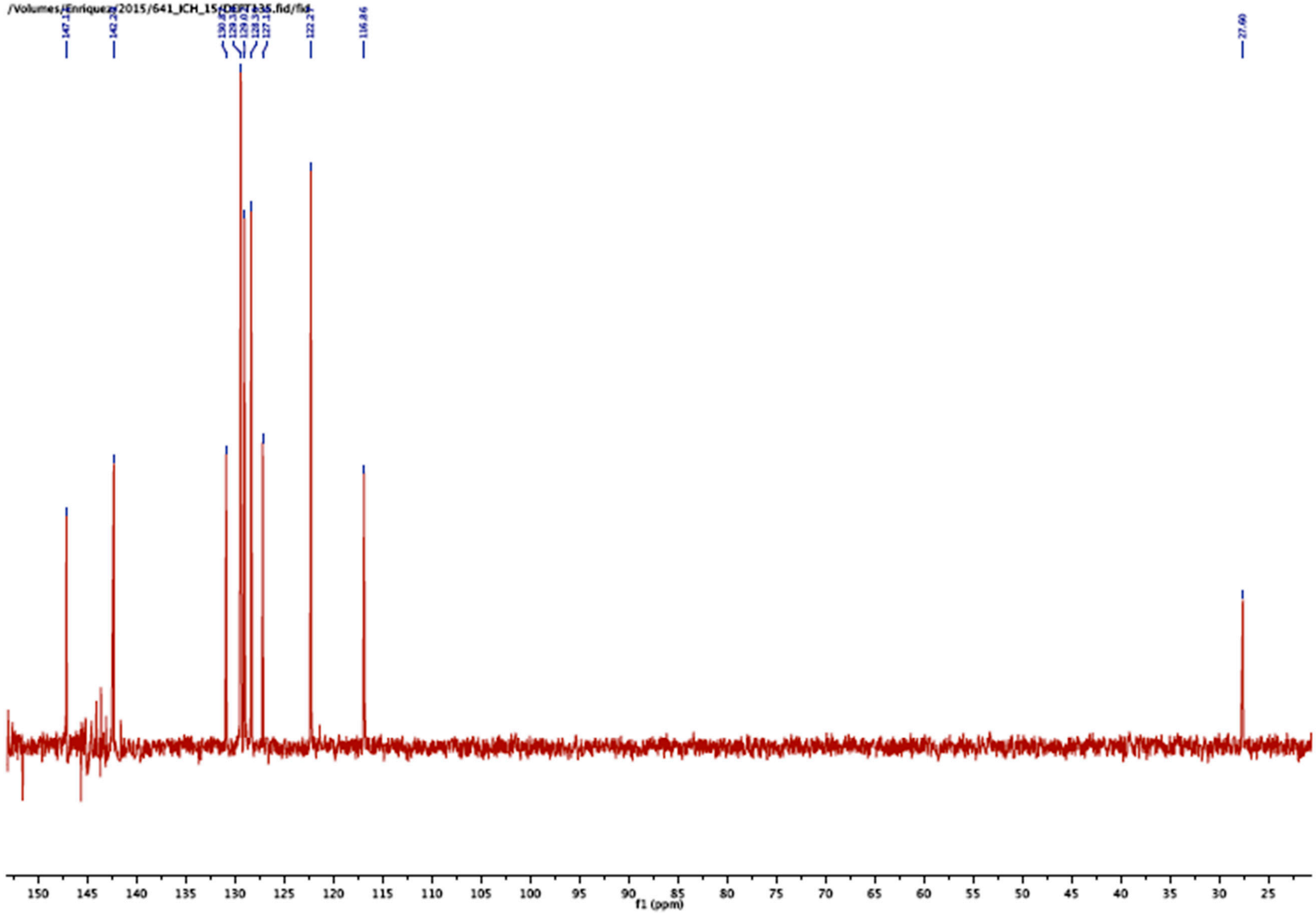
/Volumes/Enriquez/2015/641_ICH_15/DEPT135.fid/fid
116.86
27.60
150 145 140 135 130 125 120 115 110 105 100 95 90 85 80 75 70 65 60 55 50 45 40 35 30 25
f1 (ppm)

(**b**)

NMR *Retro*-Curcuminoid **9**

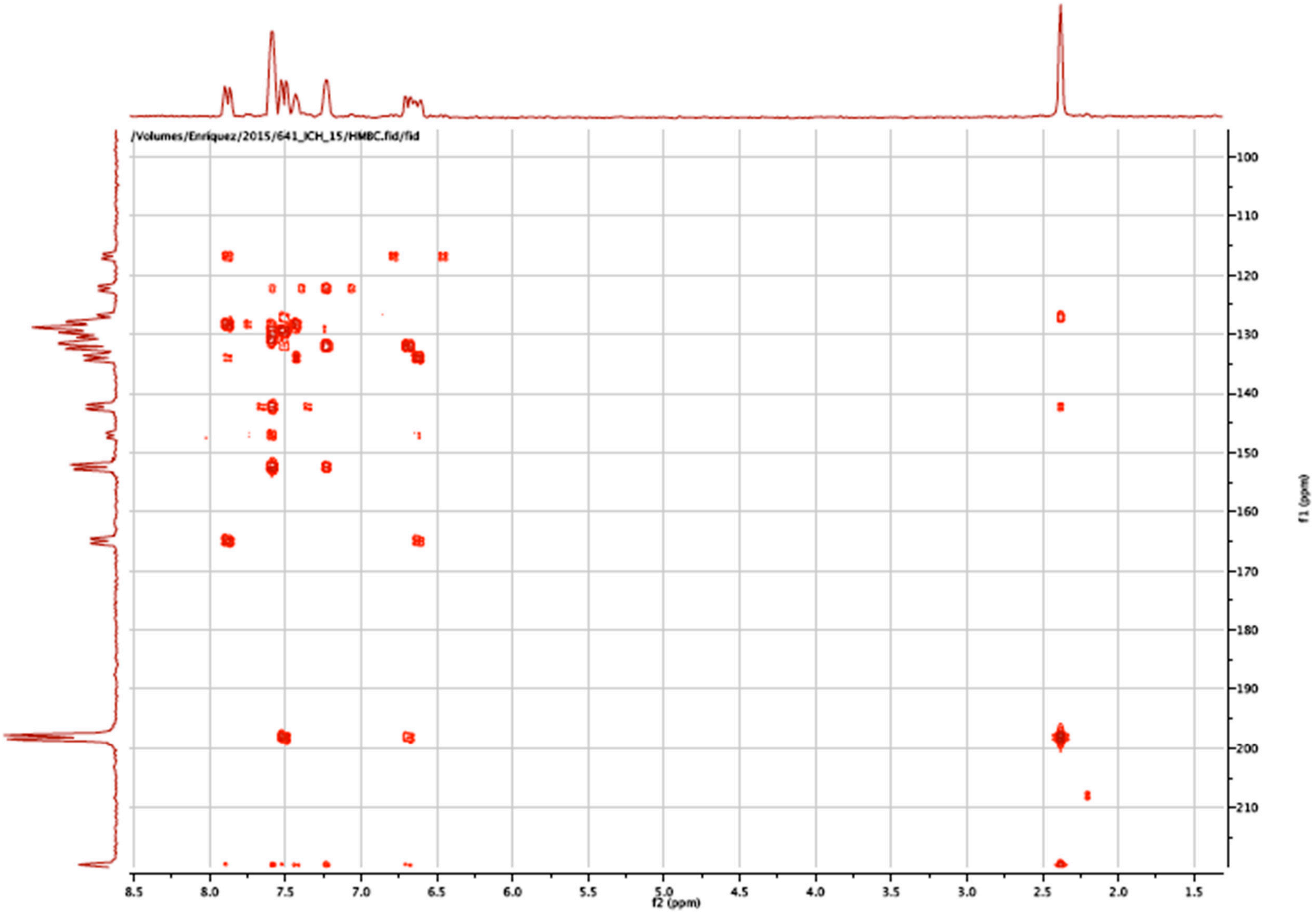

NMR *Retro*-Curcuminoid **9**

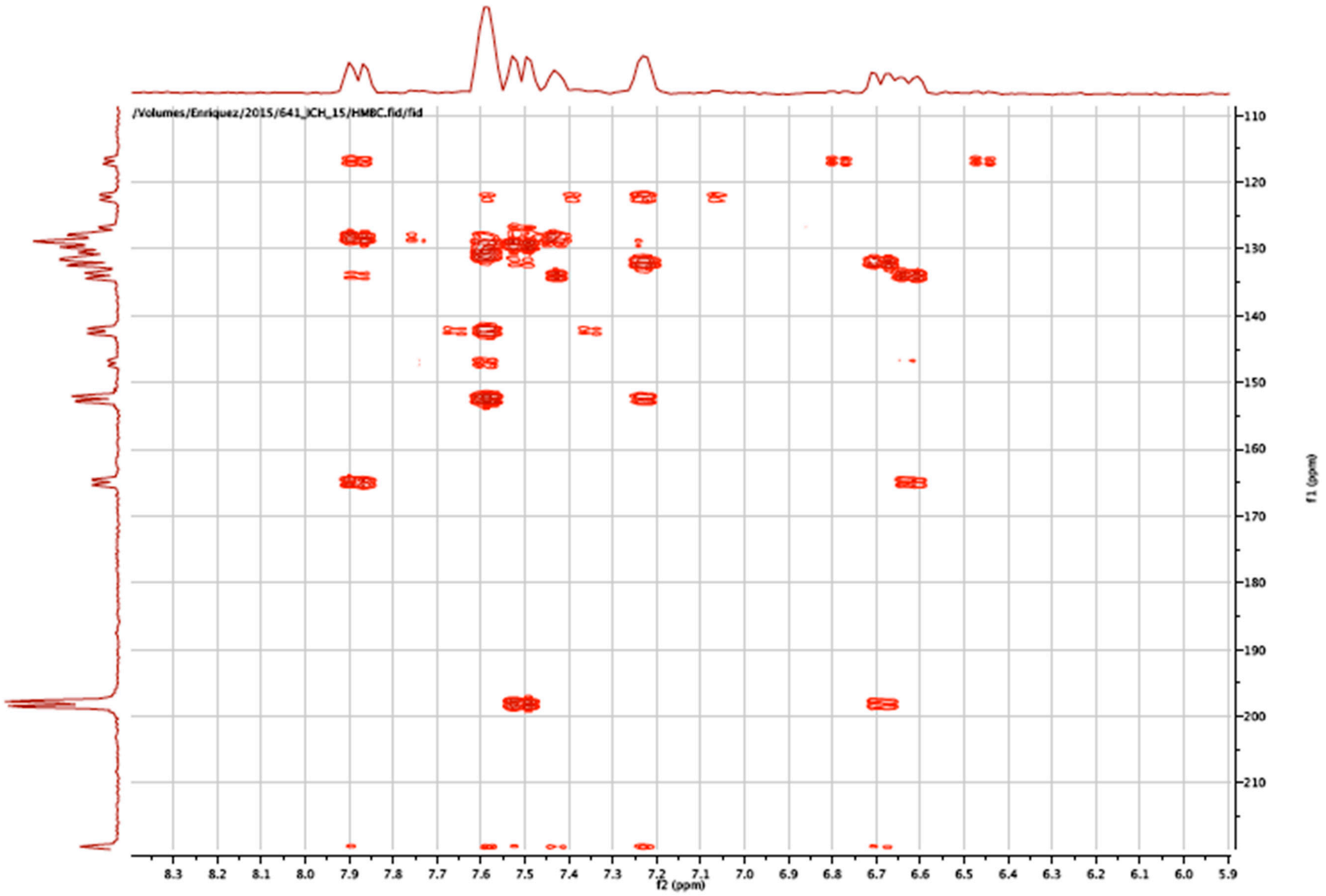

NMR *Retro*-Curcuminoid **9**

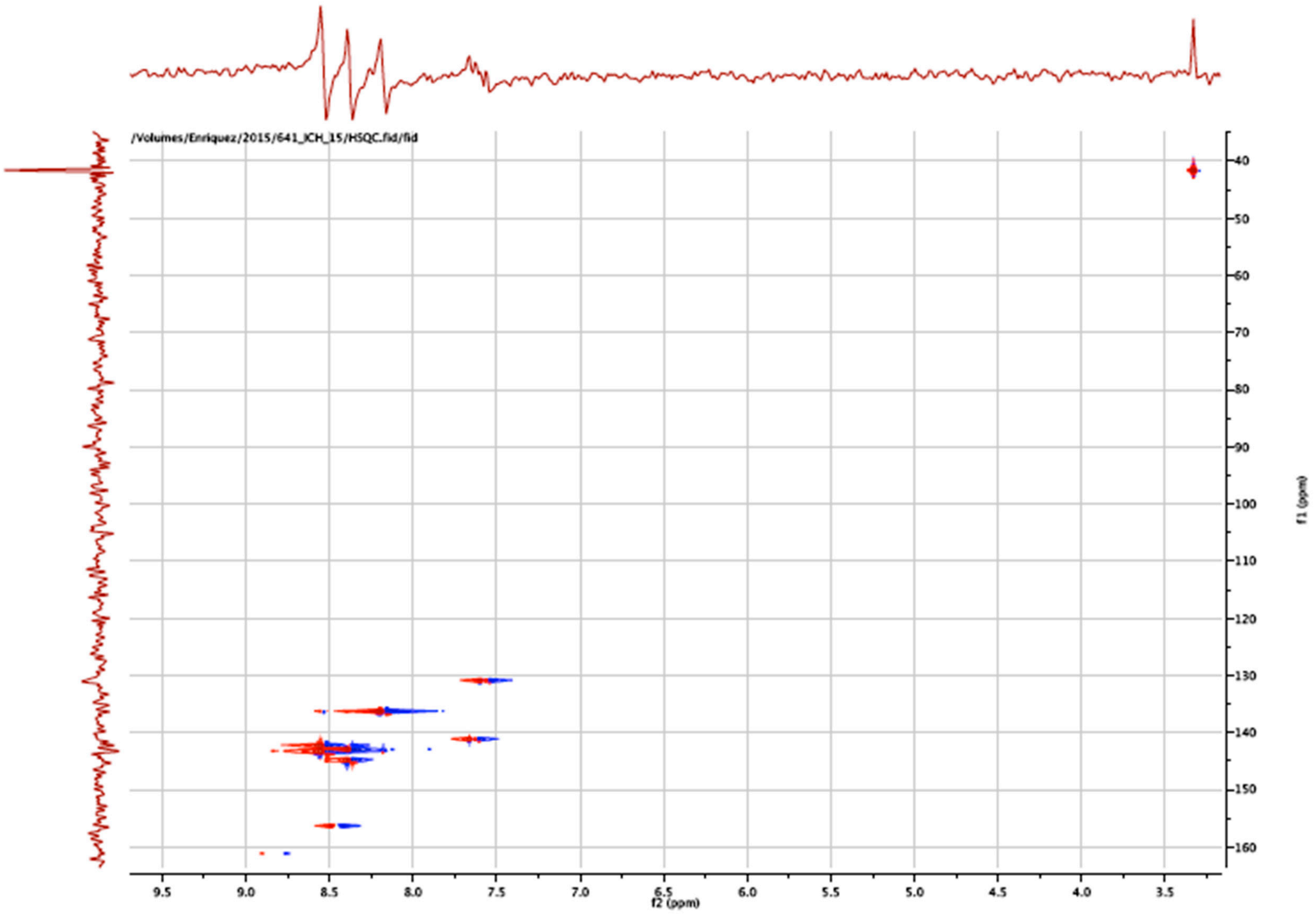

NMR *Retro*-Curcuminoid **9**

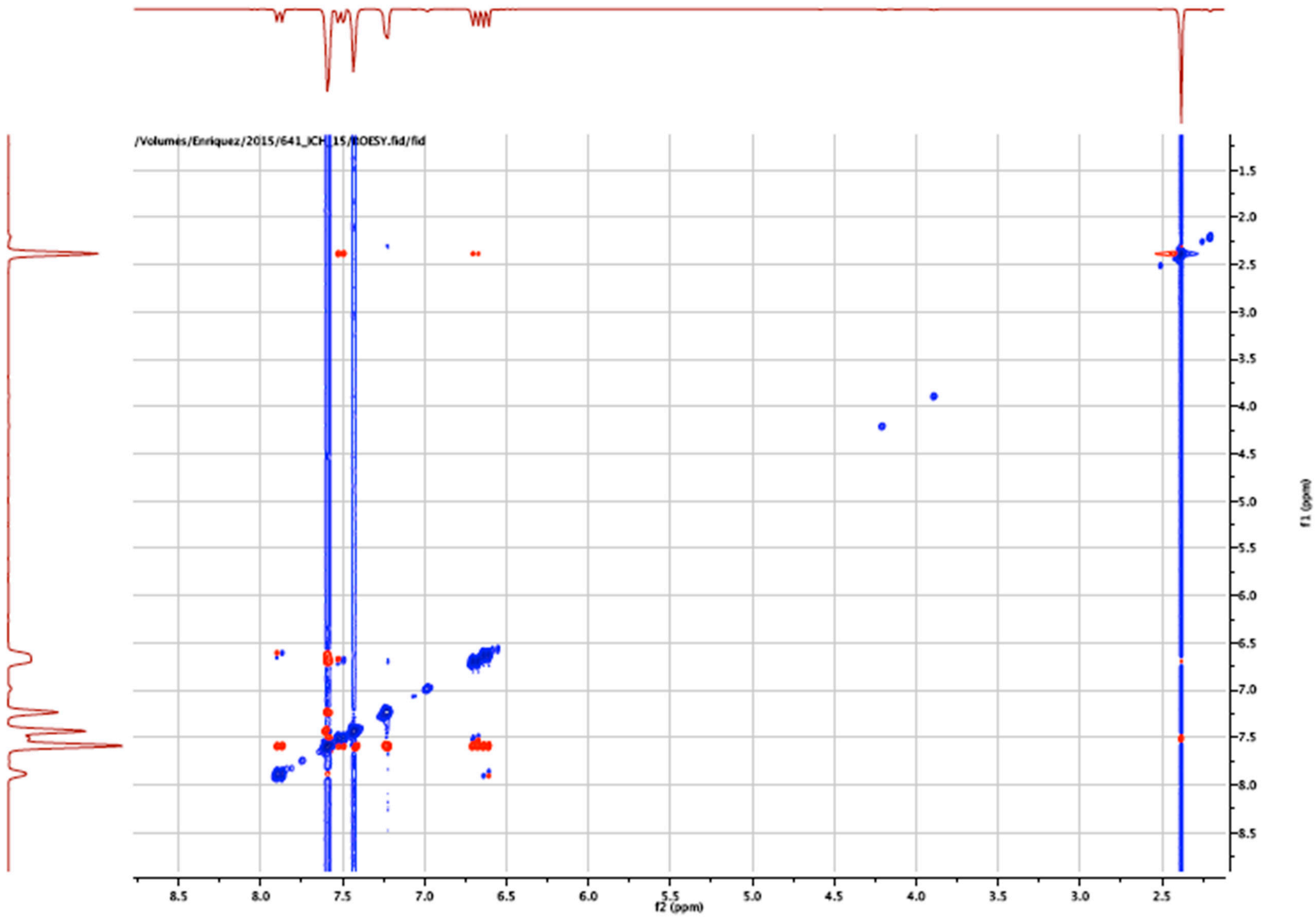

NMR *Retro*-Curcuminoid **10**

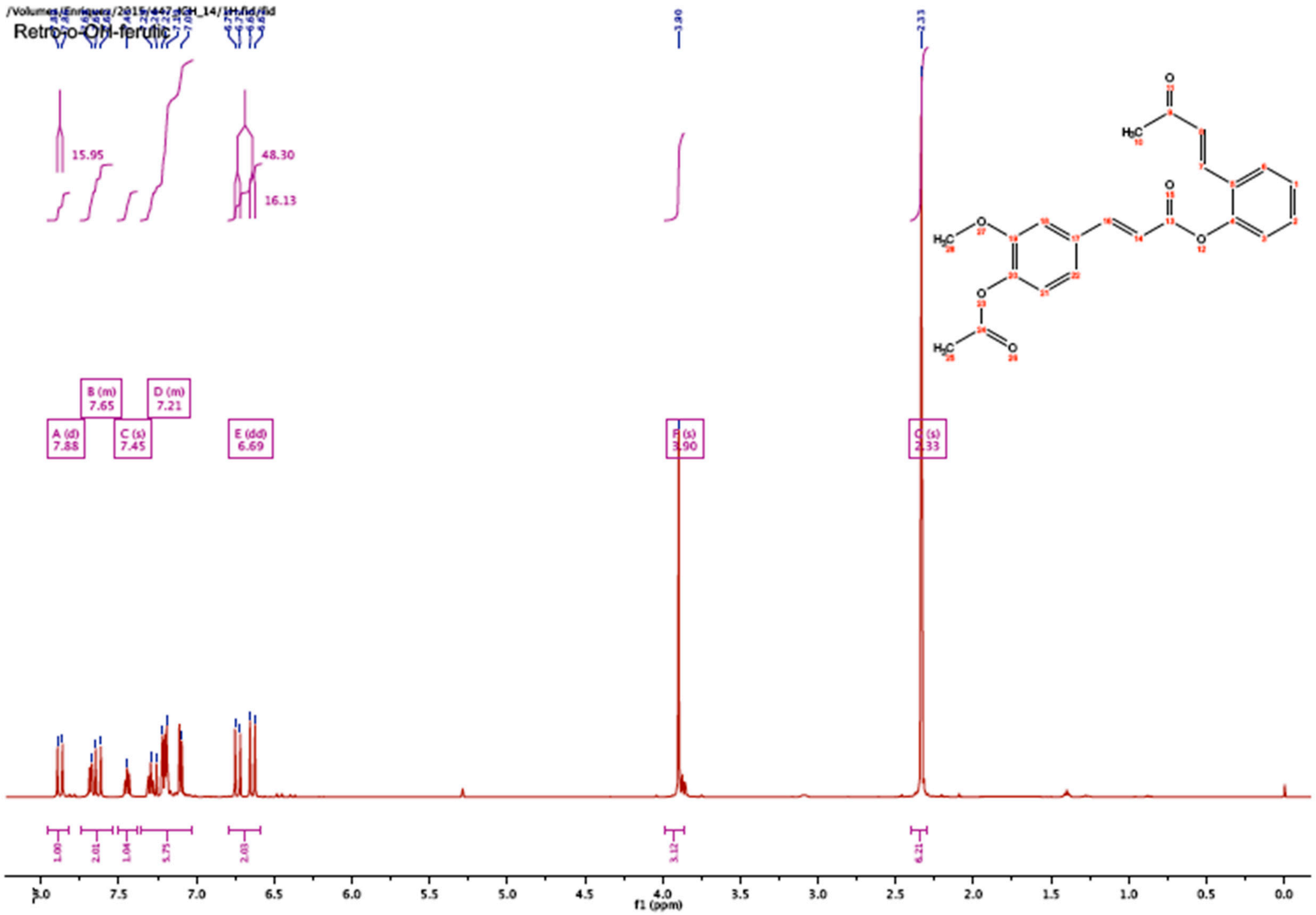

NMR *Retro*-Curcuminoid **10**

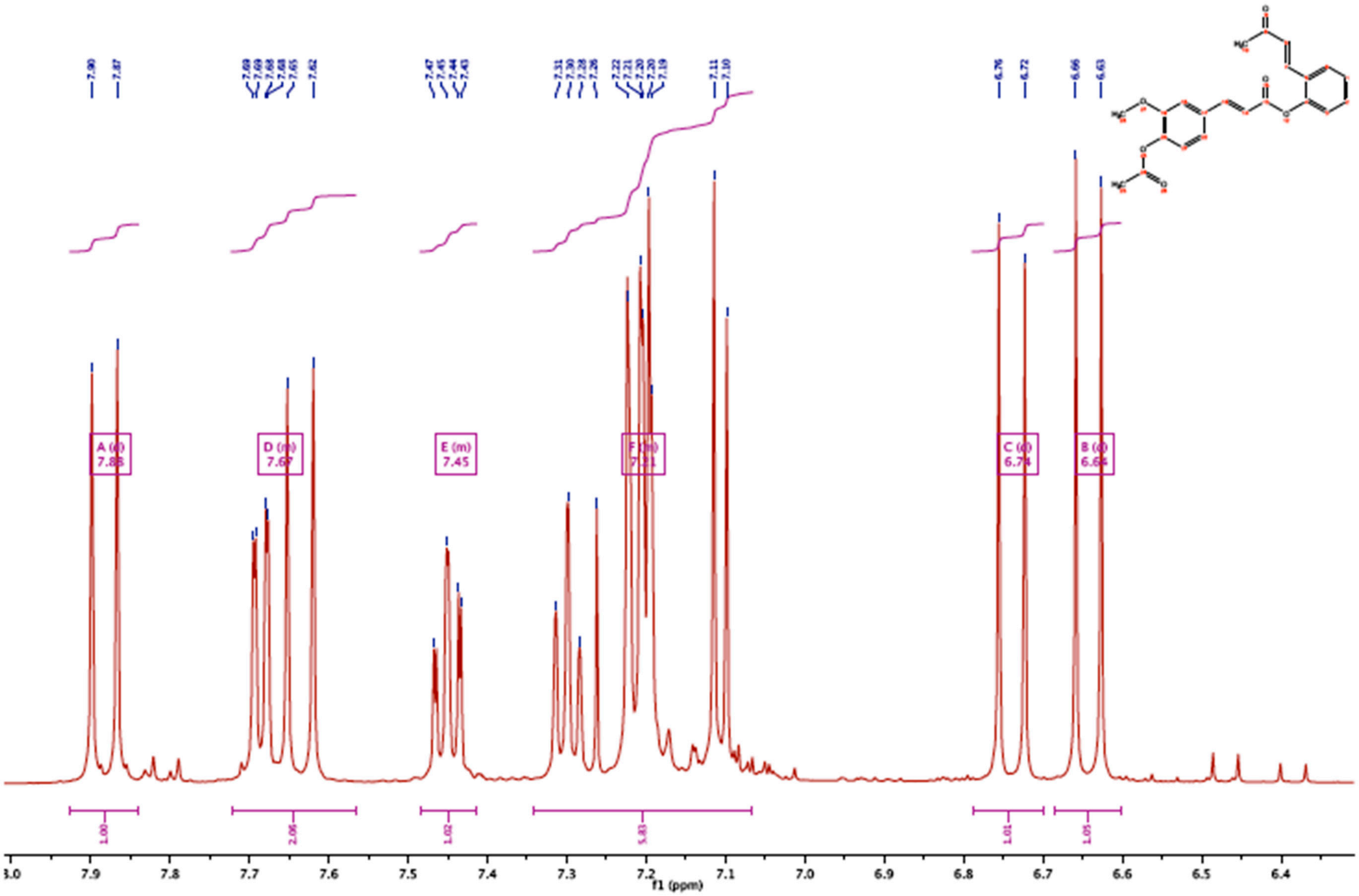

NMR *Retro*-Curcuminoid **10**

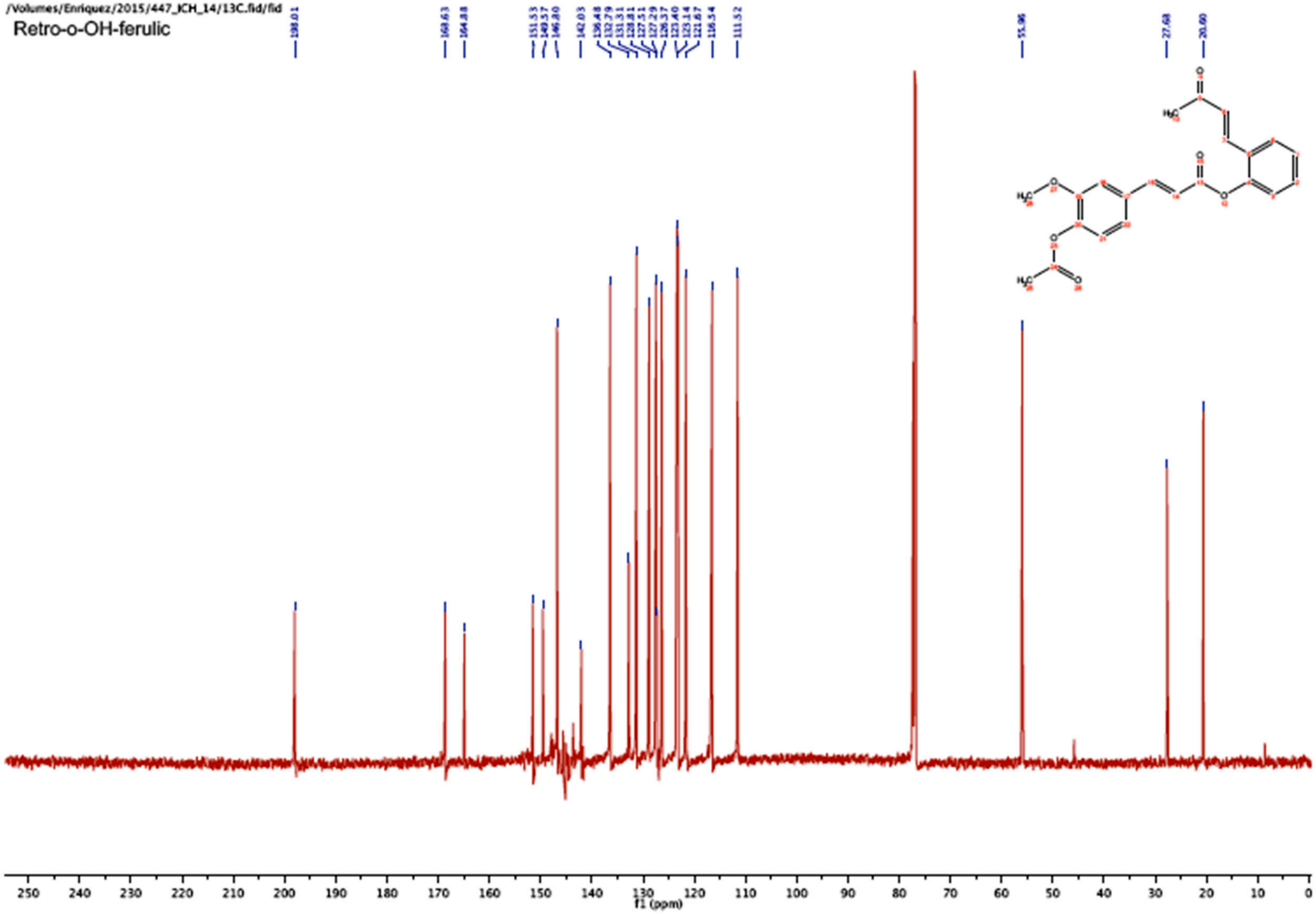

NMR *Retro*-Curcuminoid **10**

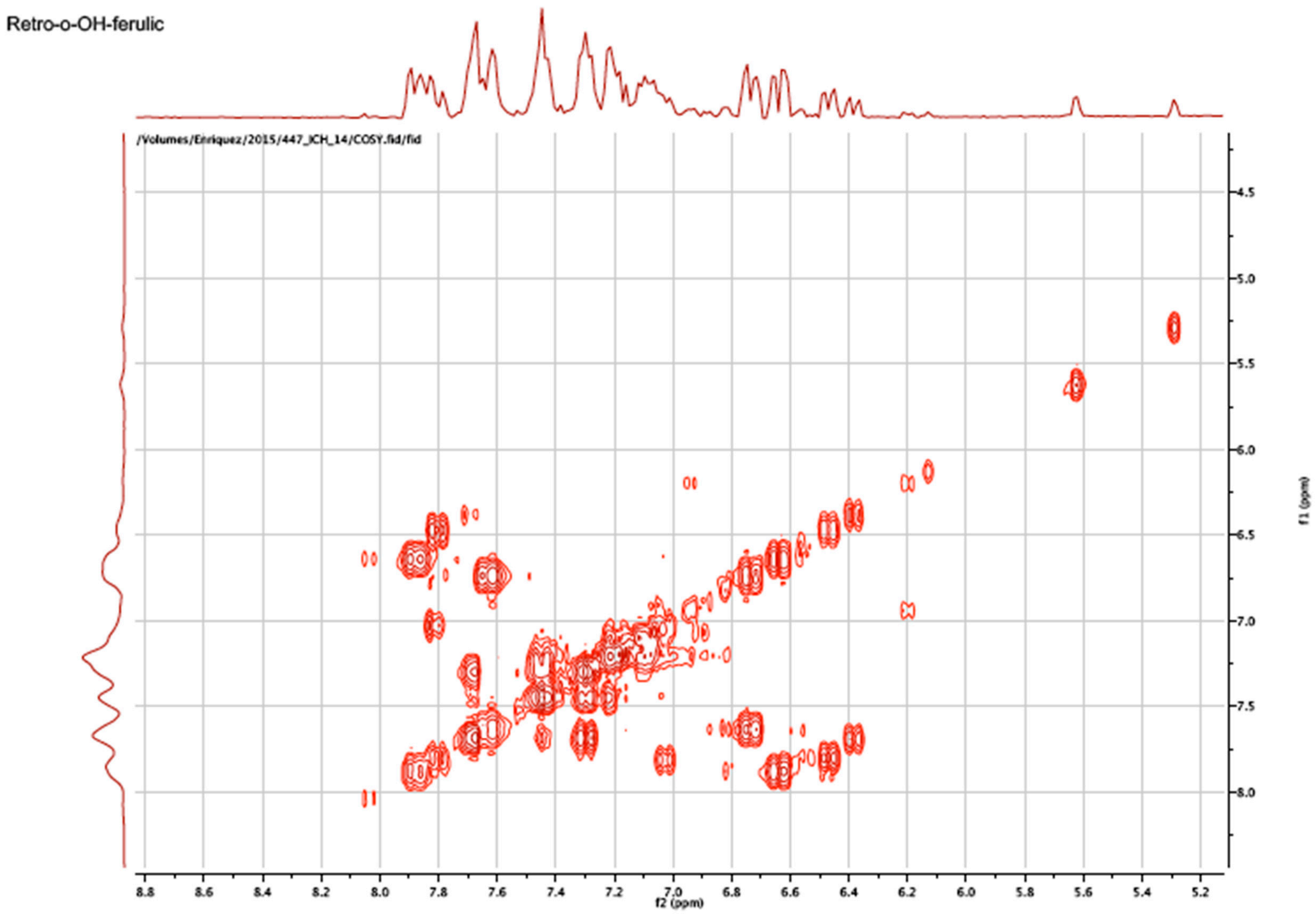

NMR *Retro*-Curcuminoid **10**

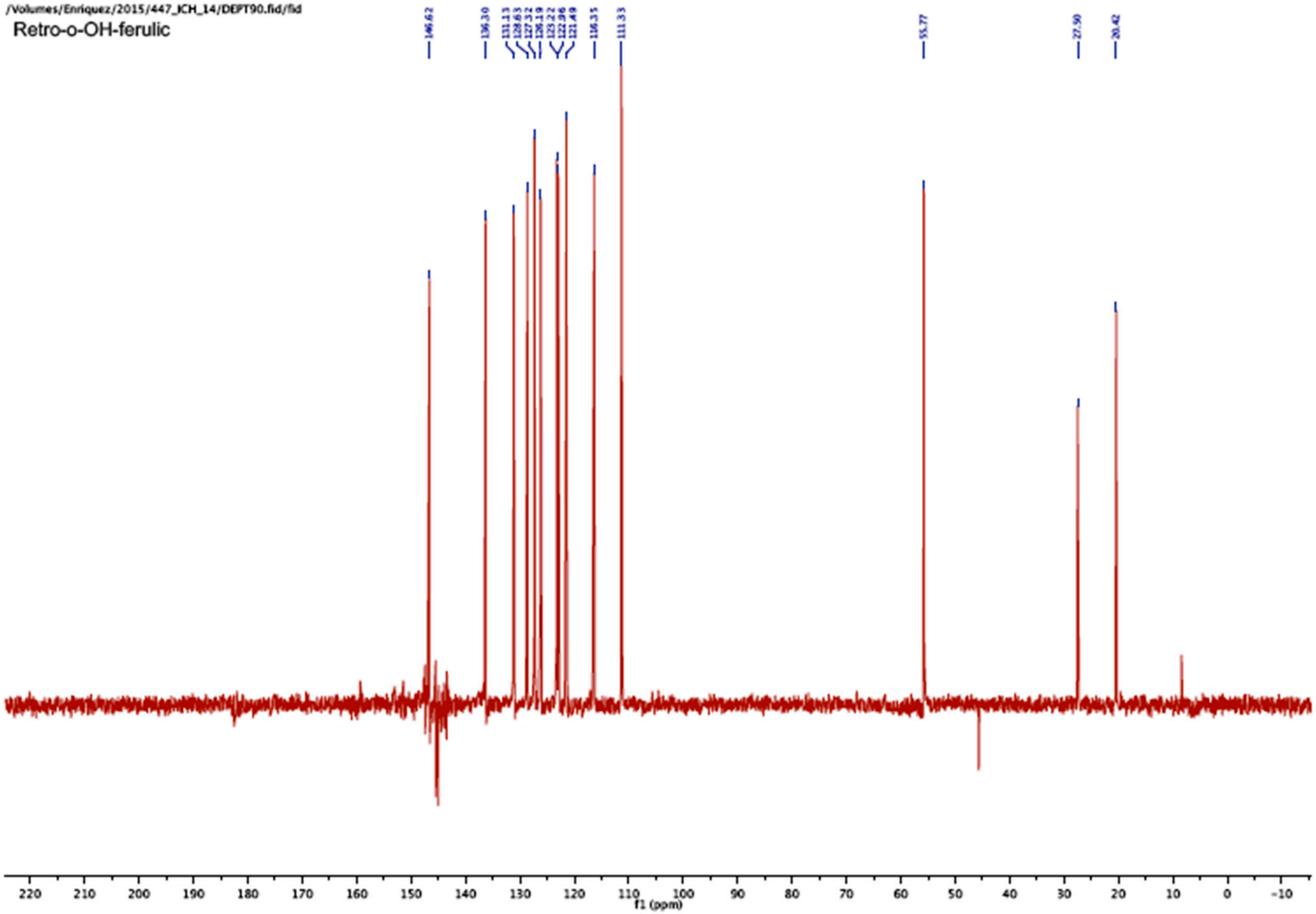

NMR *Retro*-Curcuminoid **10**

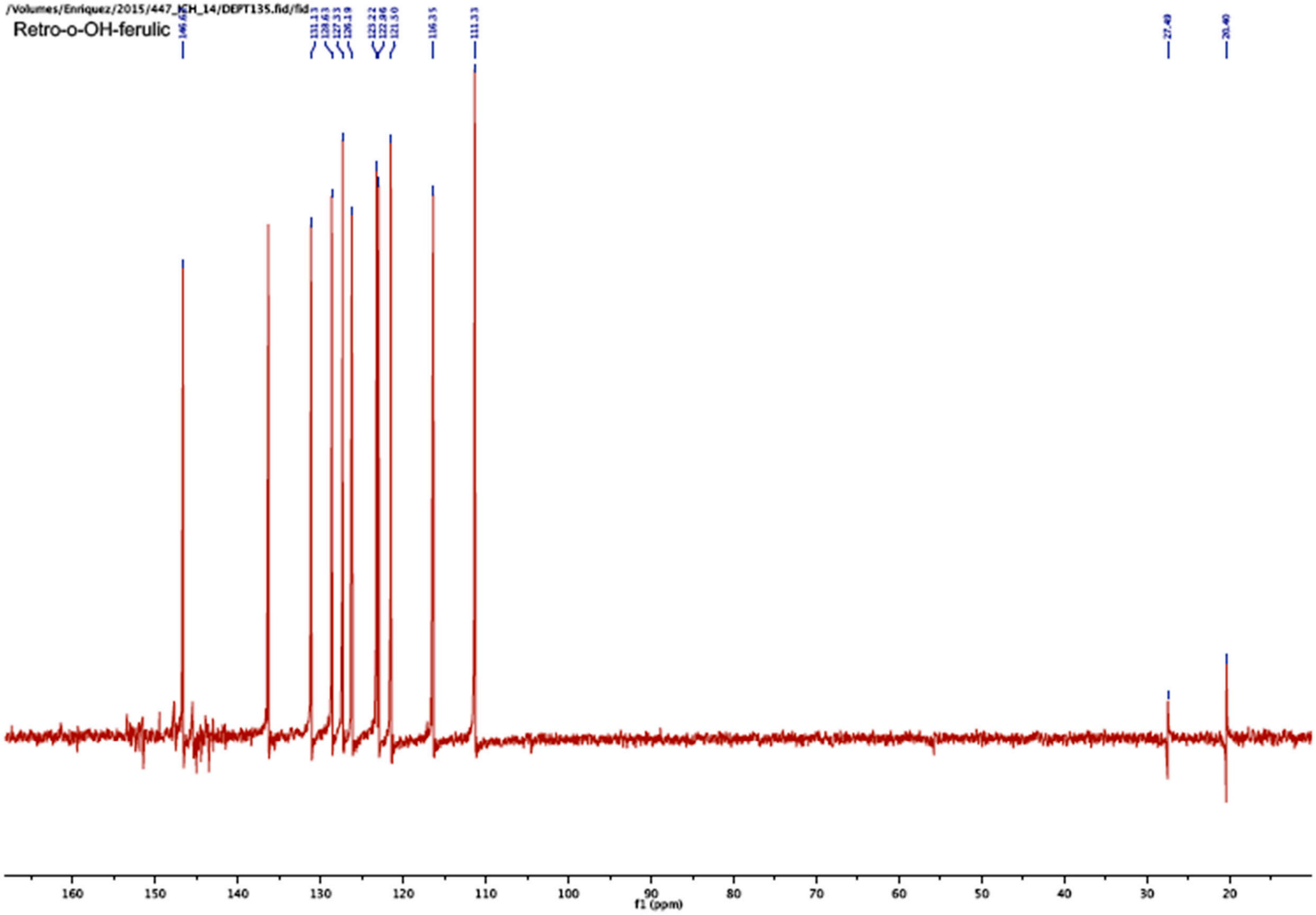

NMR *Retro*-Curcuminoid **10**

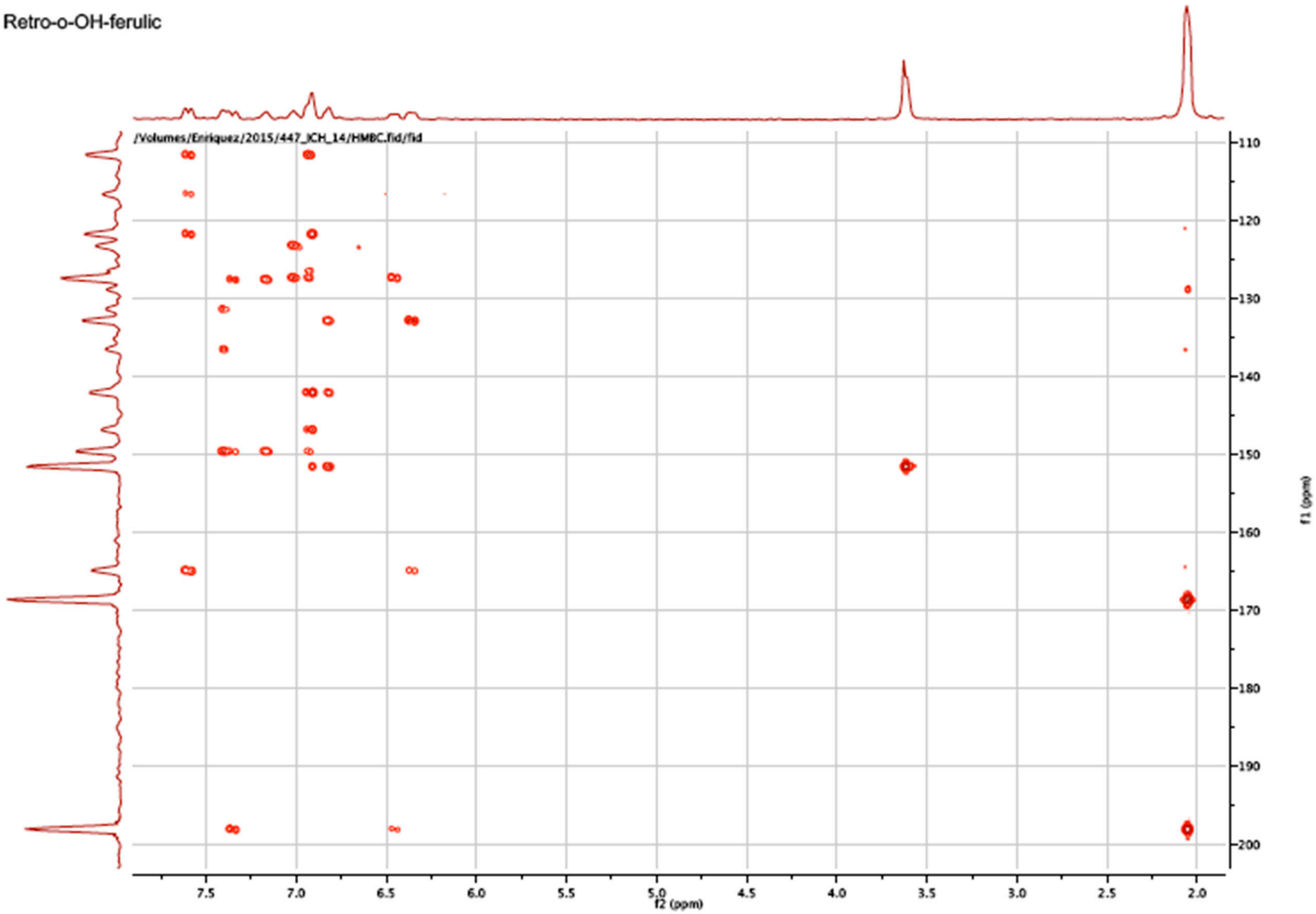

NMR *Retro*-Curcuminoid **10**

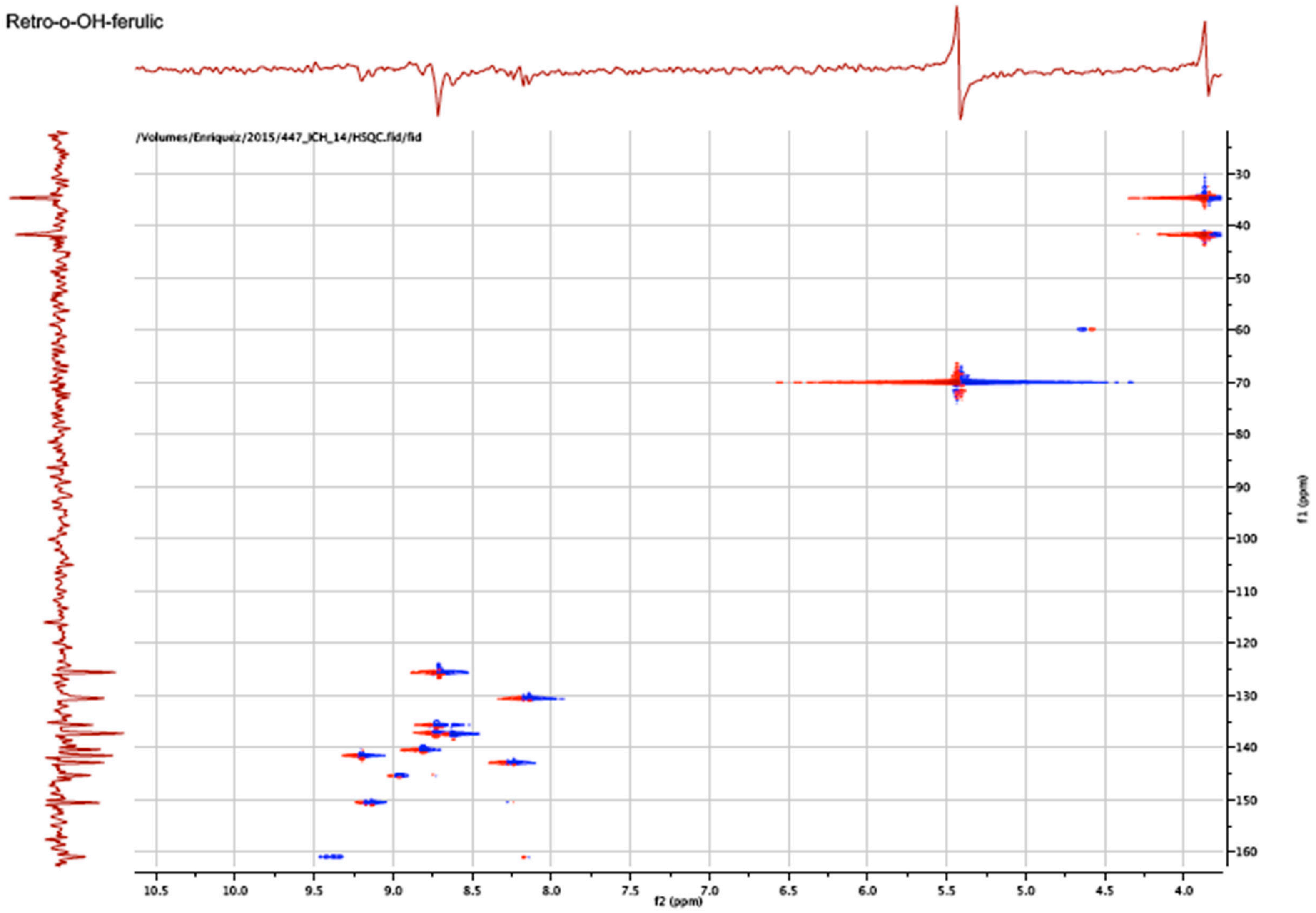

NMR *Retro*-Curcuminoid **10**

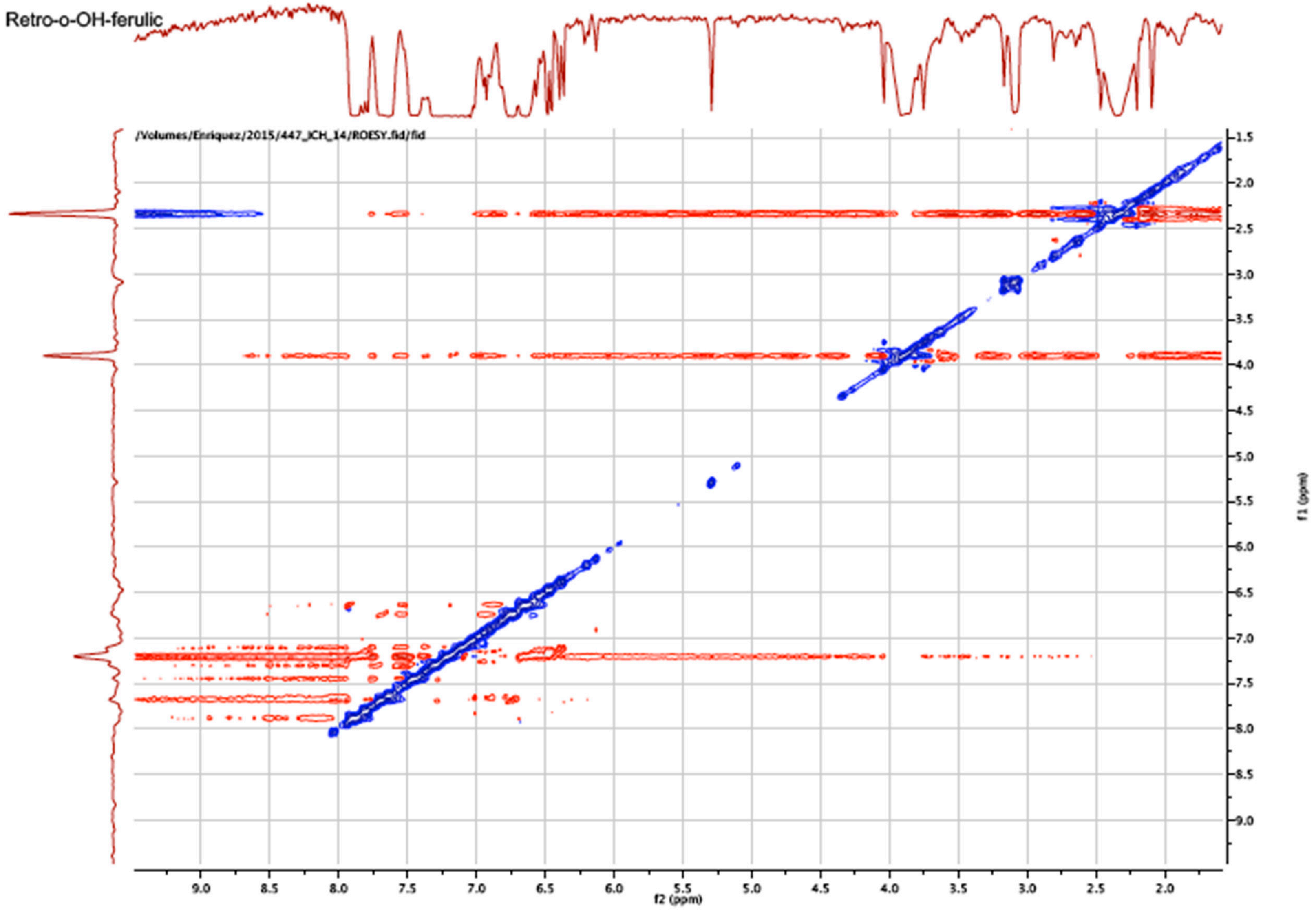

NMR *Retro*-Curcuminoid **11**

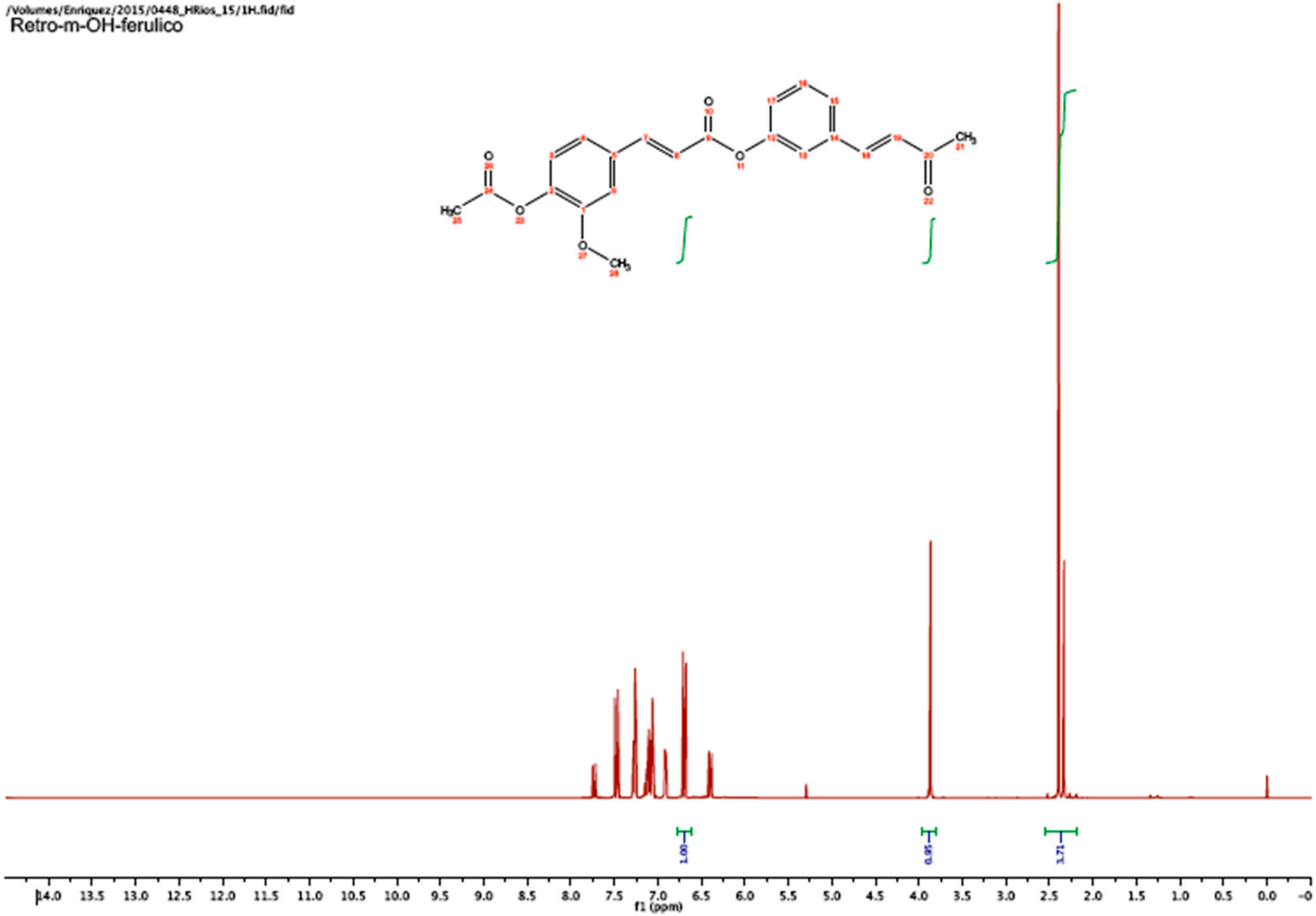

NMR *Retro*-Curcuminoid **11**

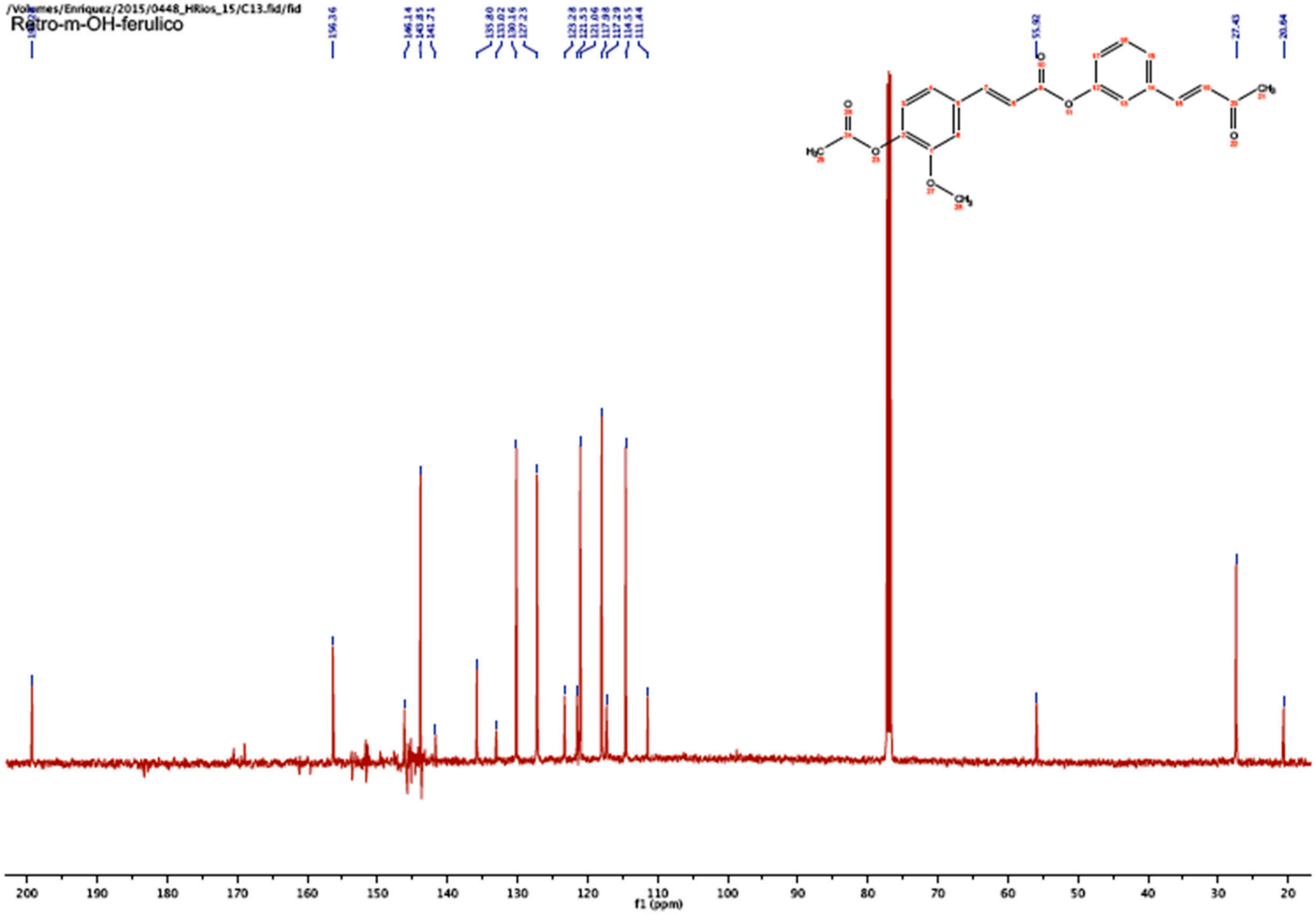

NMR *Retro*-Curcuminoid **11**

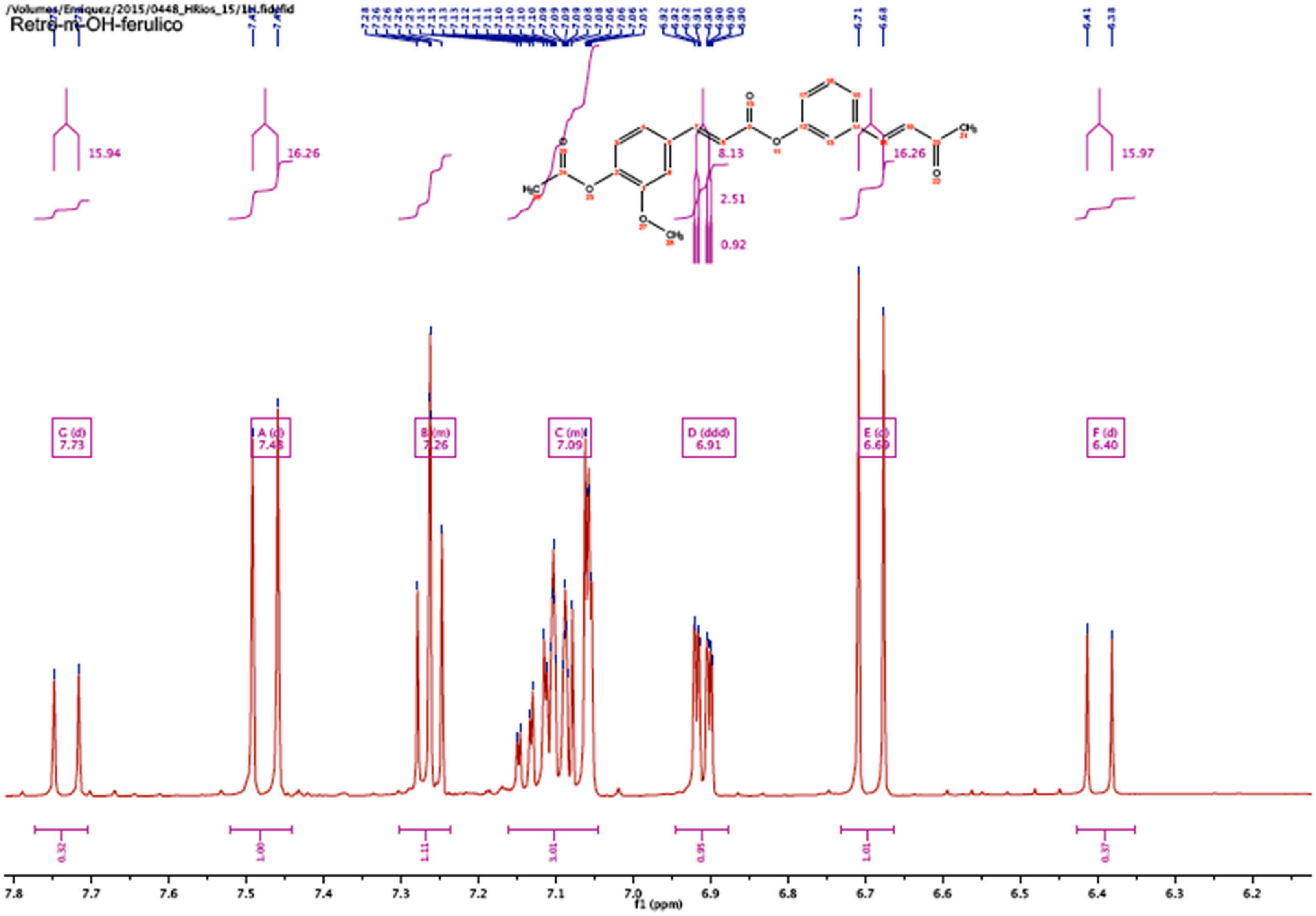

NMR *Retro*-Curcuminoid **11**

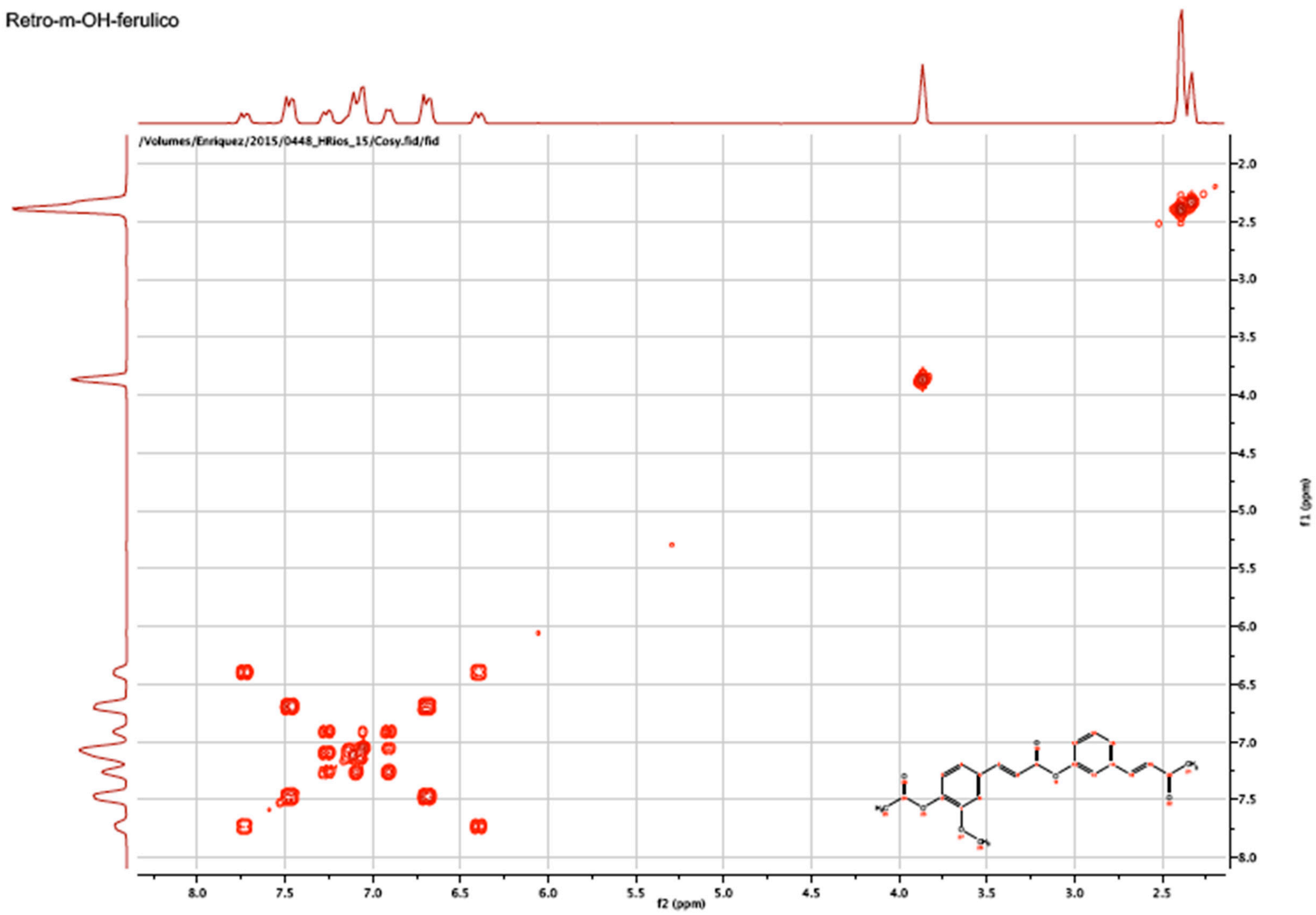

NMR *Retro*-Curcuminoid **11**

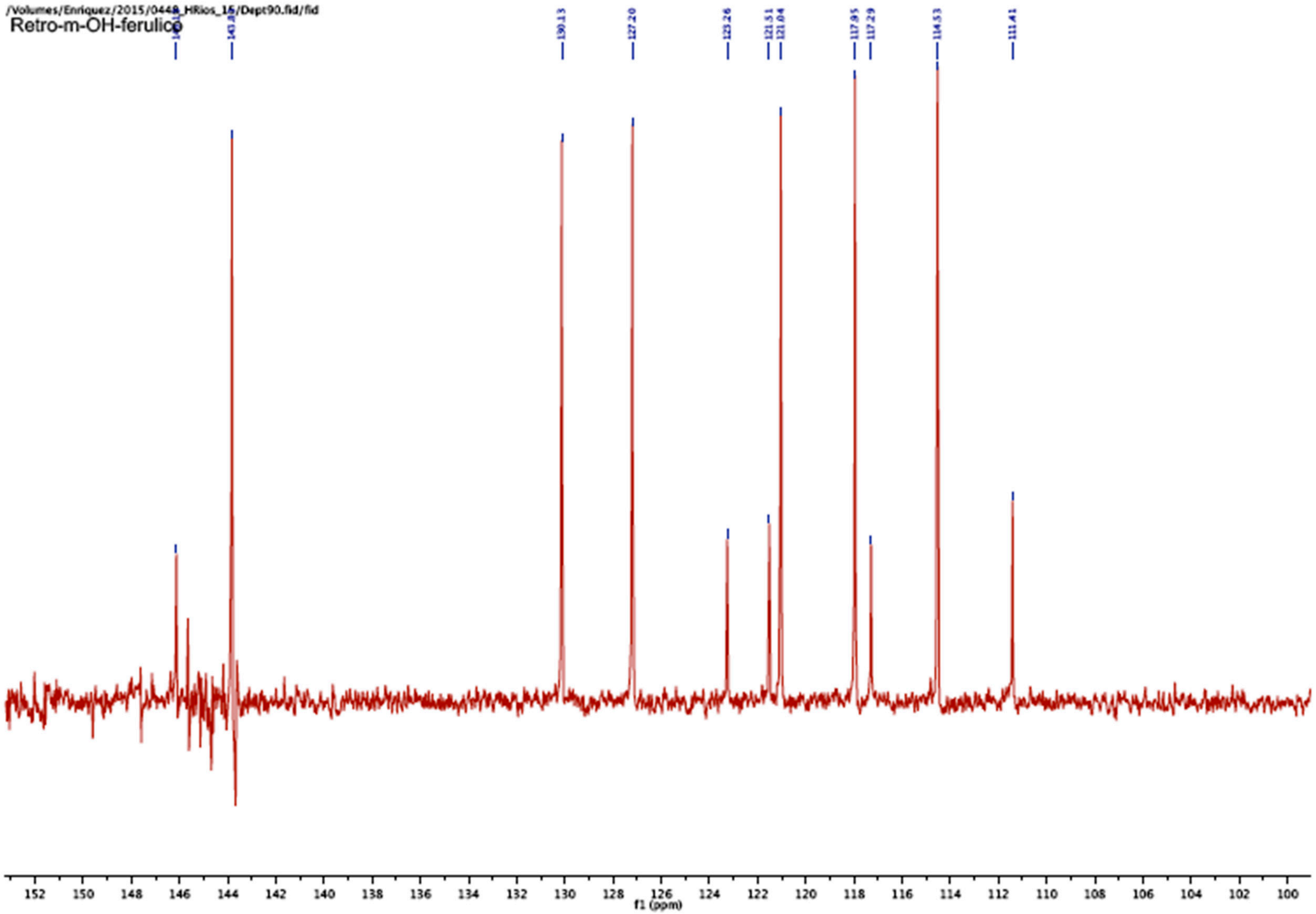

NMR *Retro*-Curcuminoid **11**

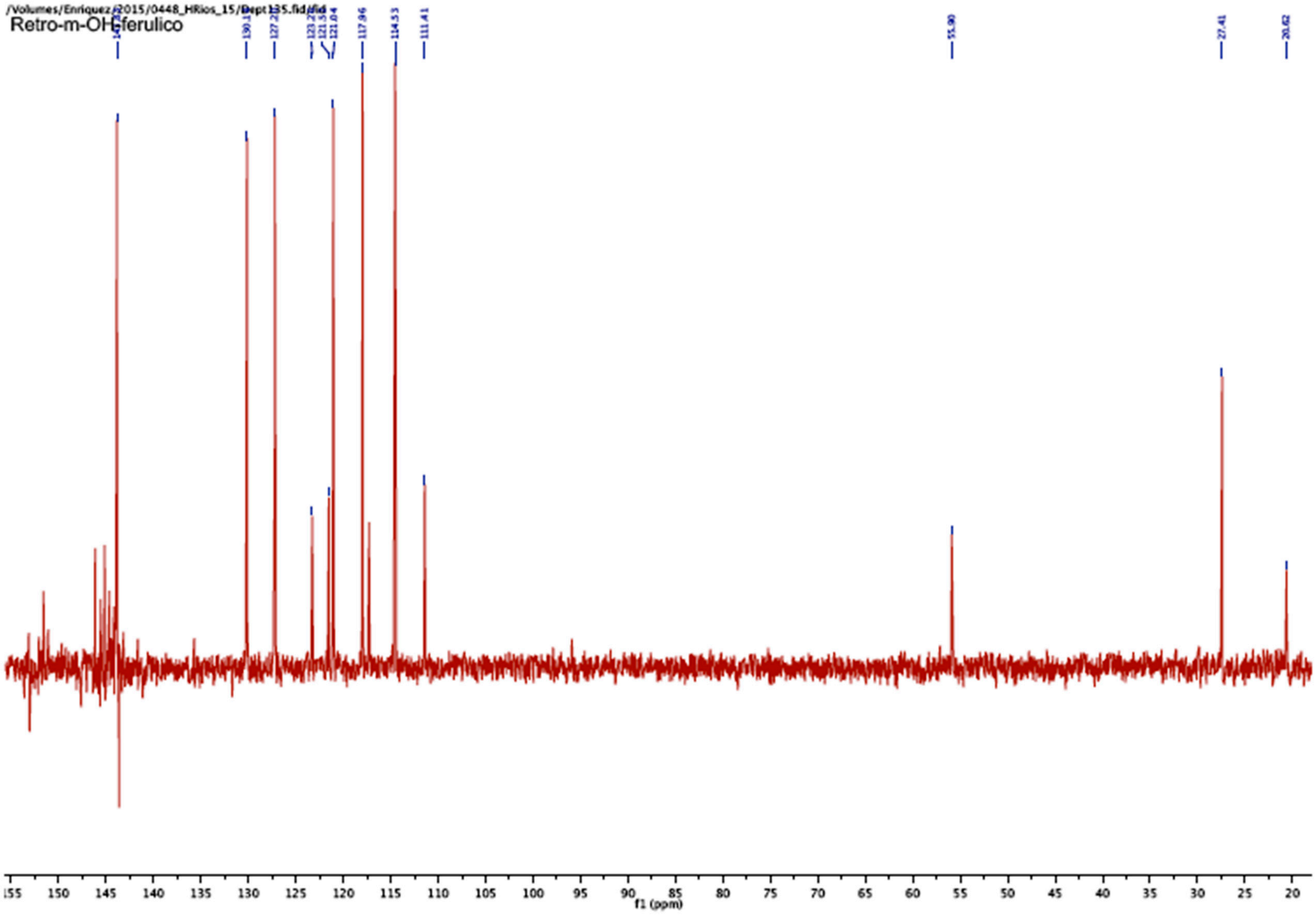

NMR *Retro*-Curcuminoid **11**

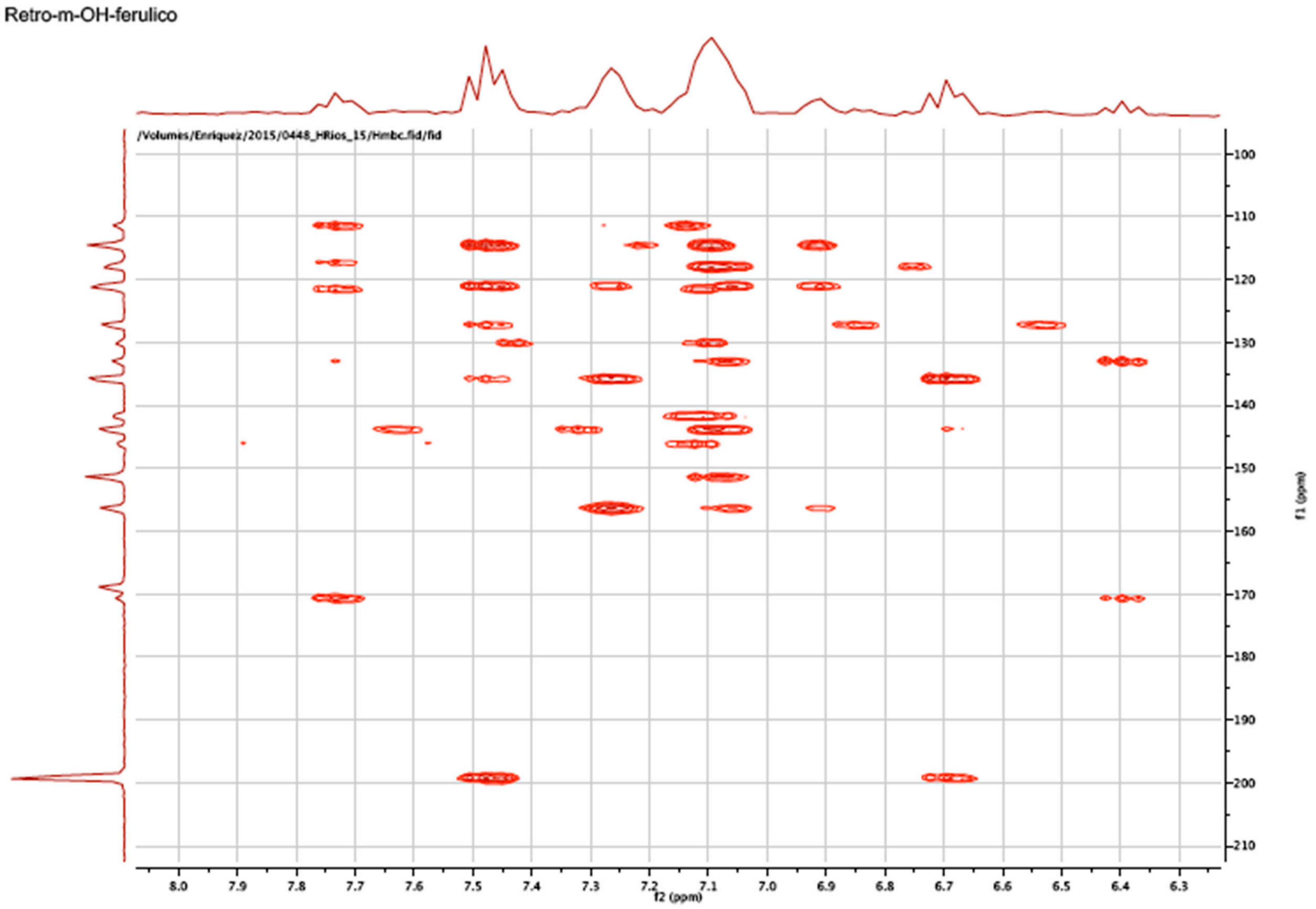

NMR *Retro*-Curcuminoid **11**

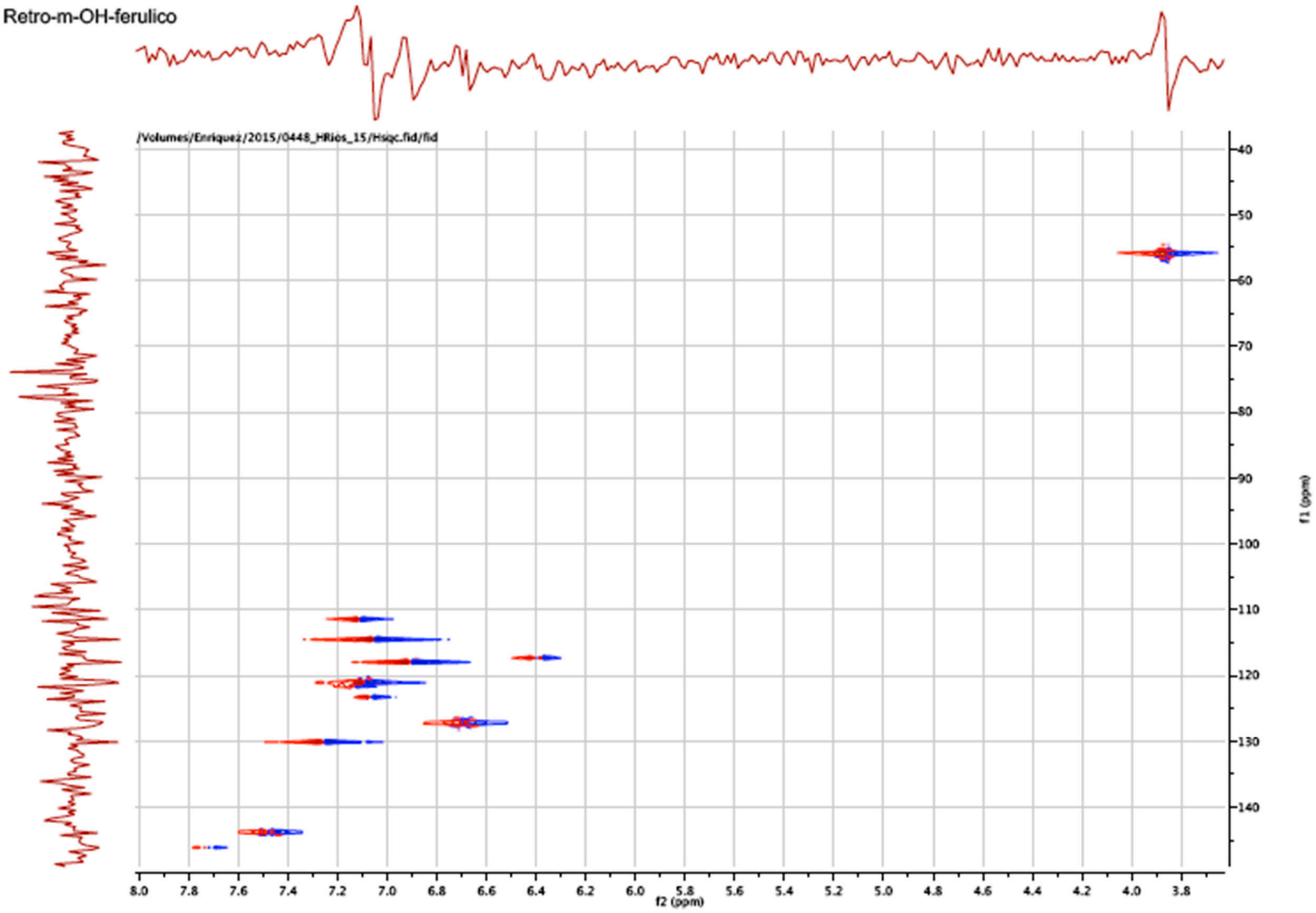

NMR *Retro*-Curcuminoid **11**

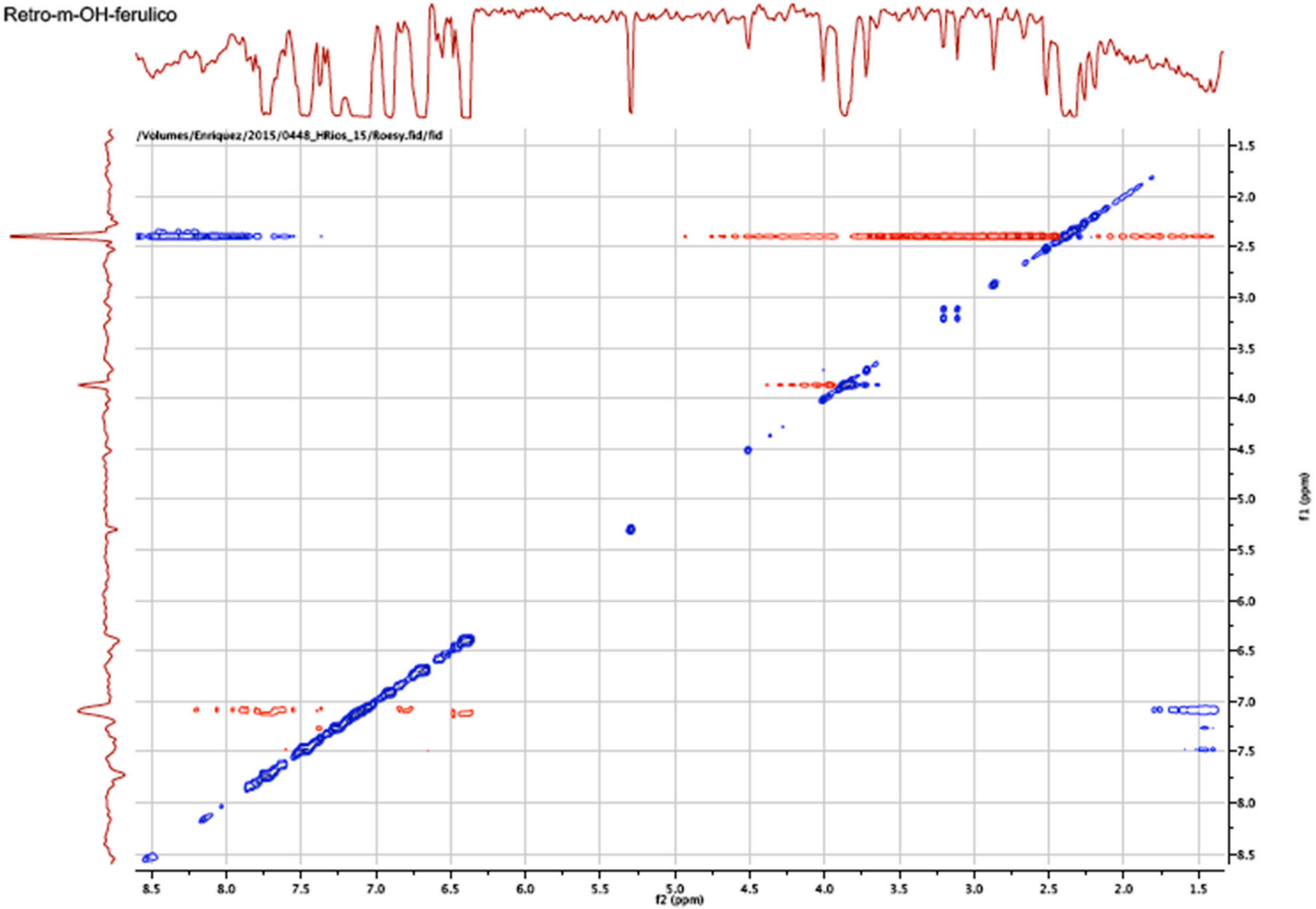

NMR *Retro*-Curcuminoid **12**

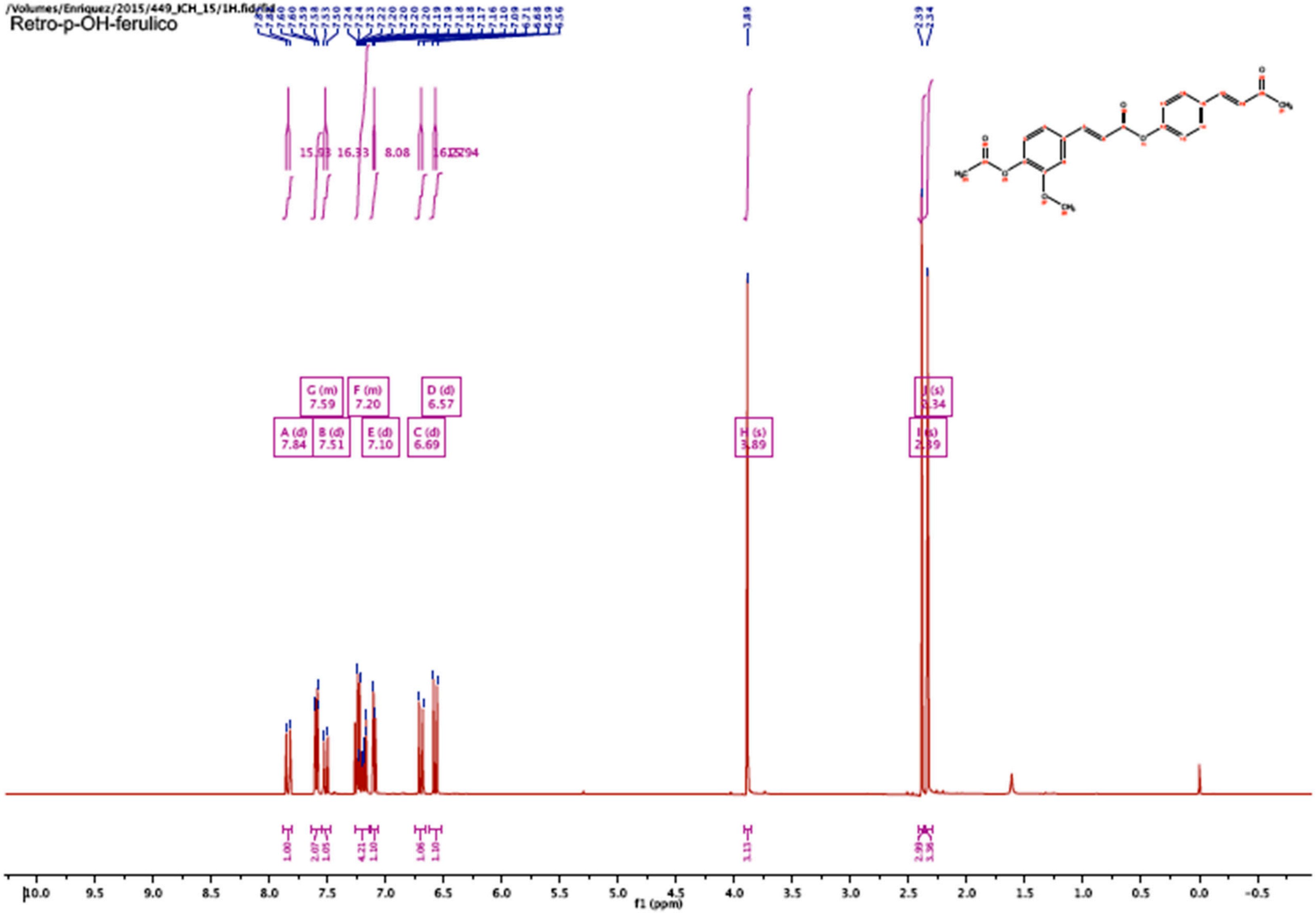

NMR *Retro*-Curcuminoid **12**

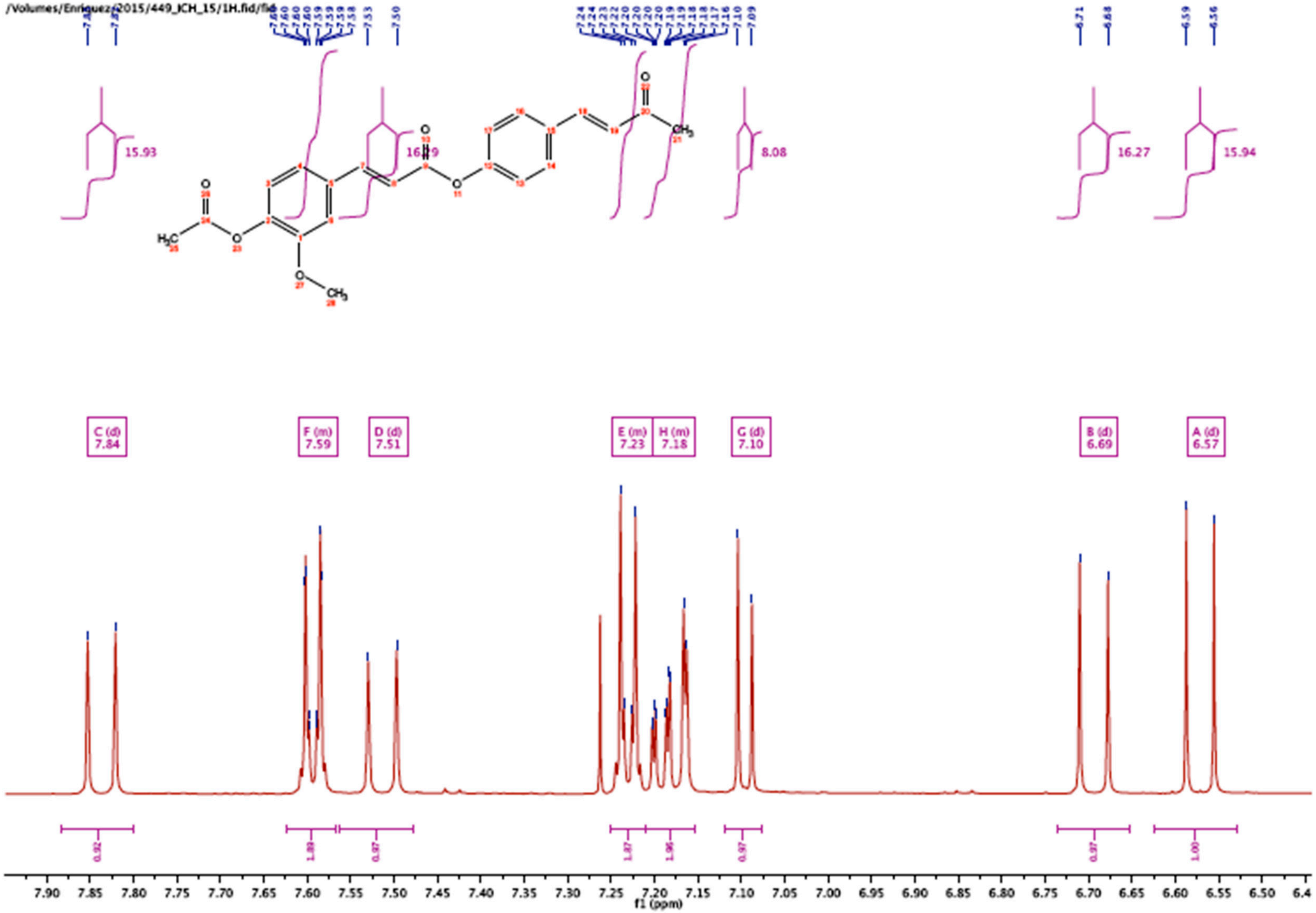

NMR *Retro*-Curcuminoid **12**

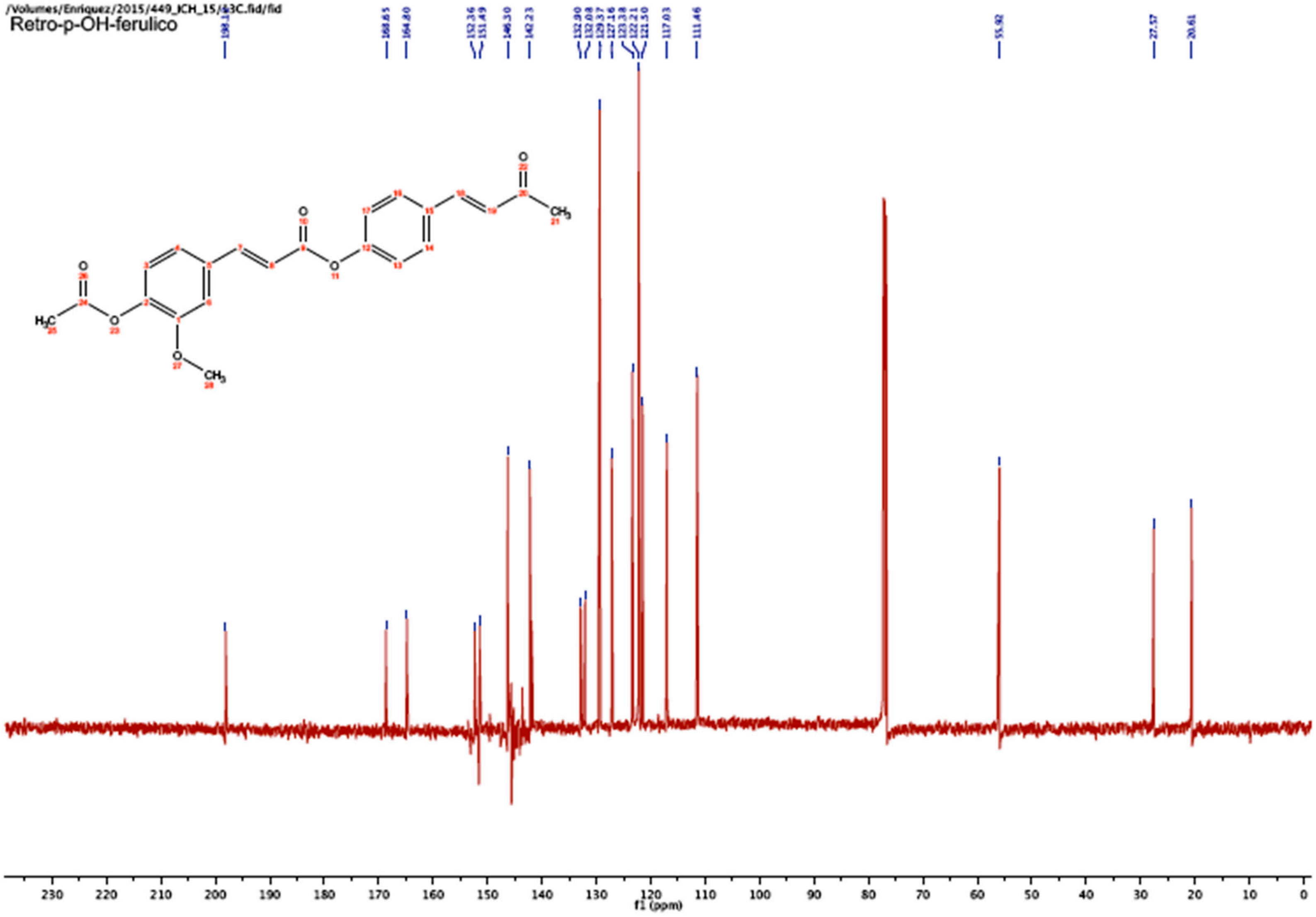

NMR *Retro*-Curcuminoid **12**

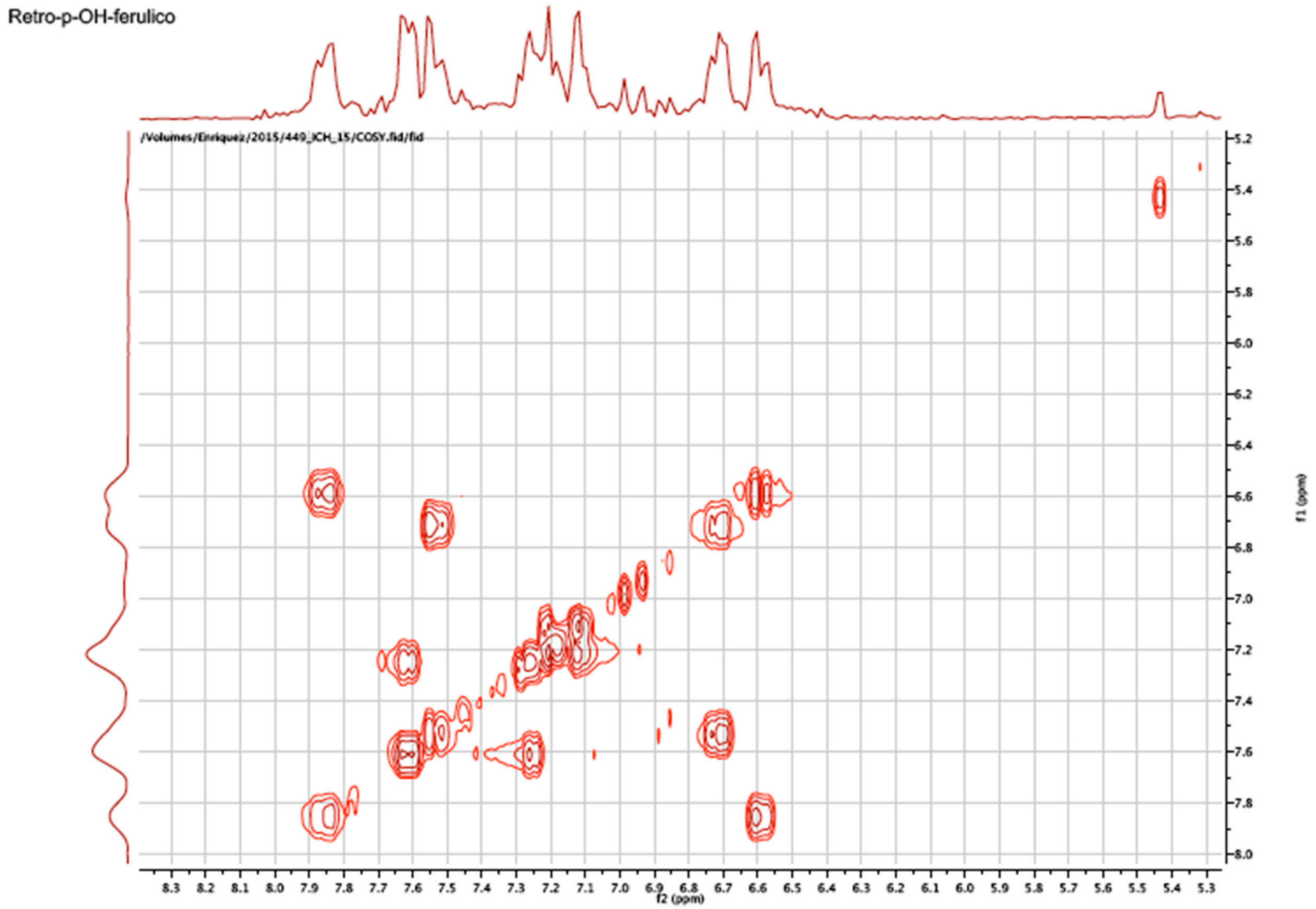

NMR *Retro*-Curcuminoid **12**

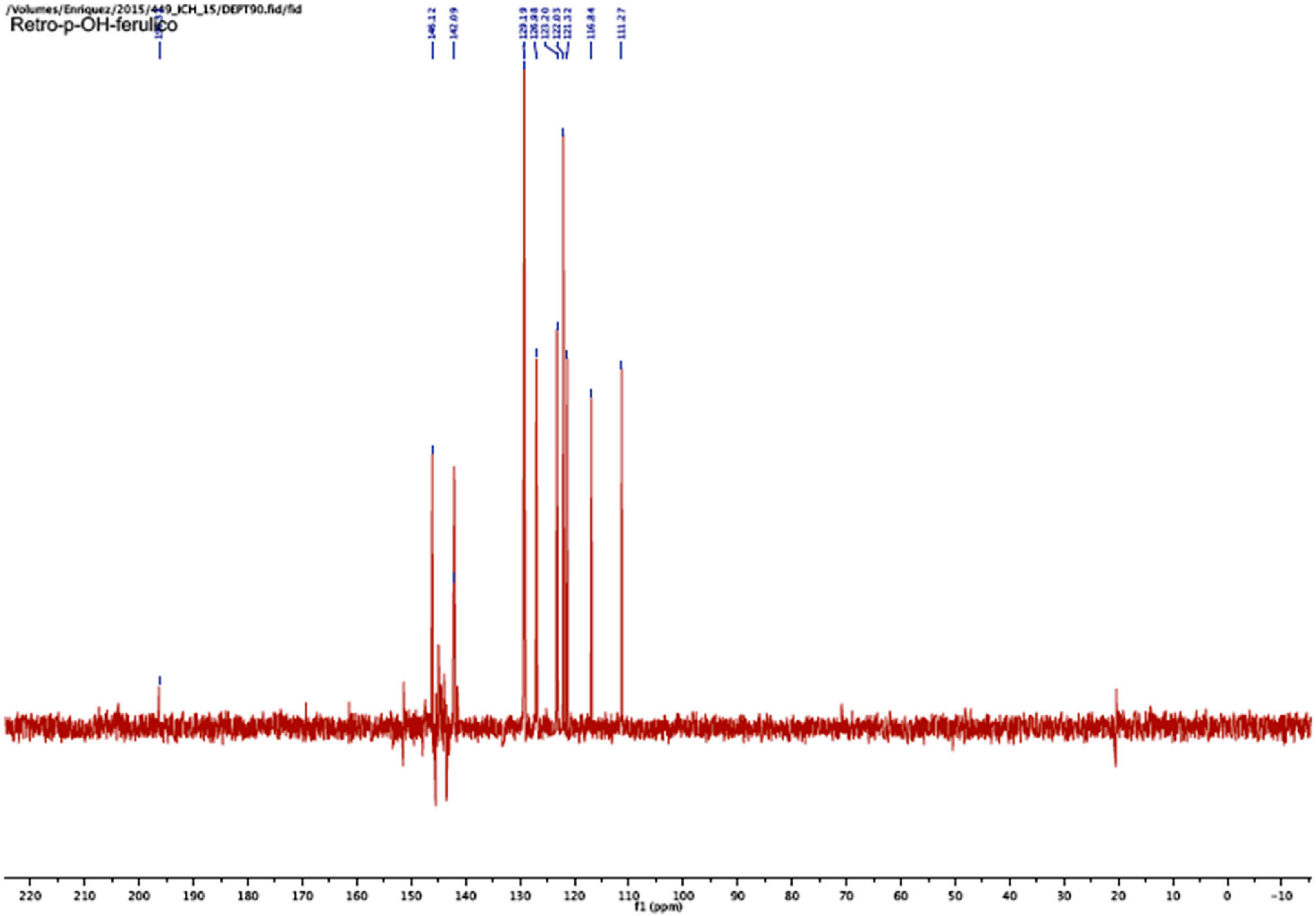

NMR *Retro*-Curcuminoid **12**

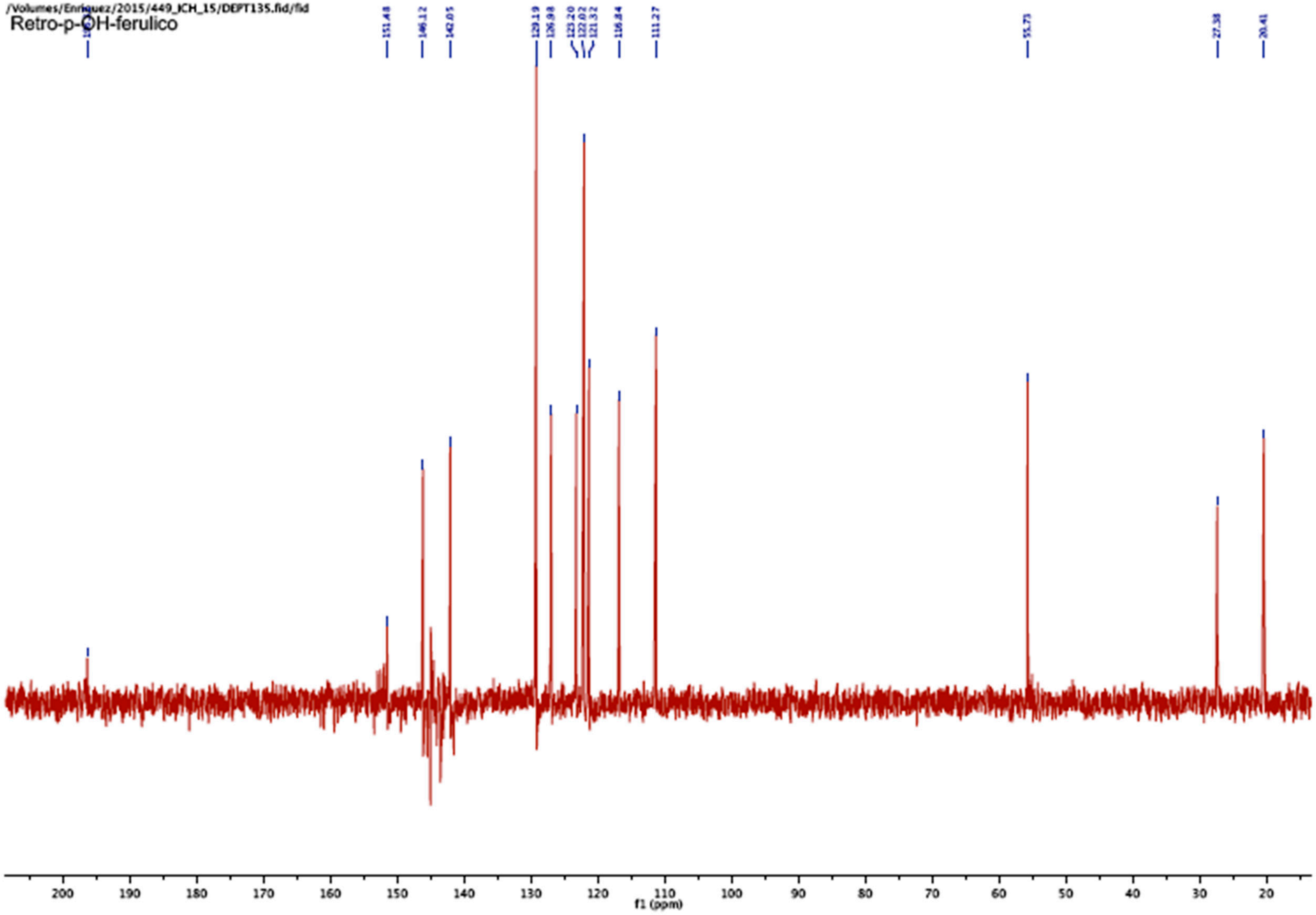

NMR *Retro*-Curcuminoid **12**

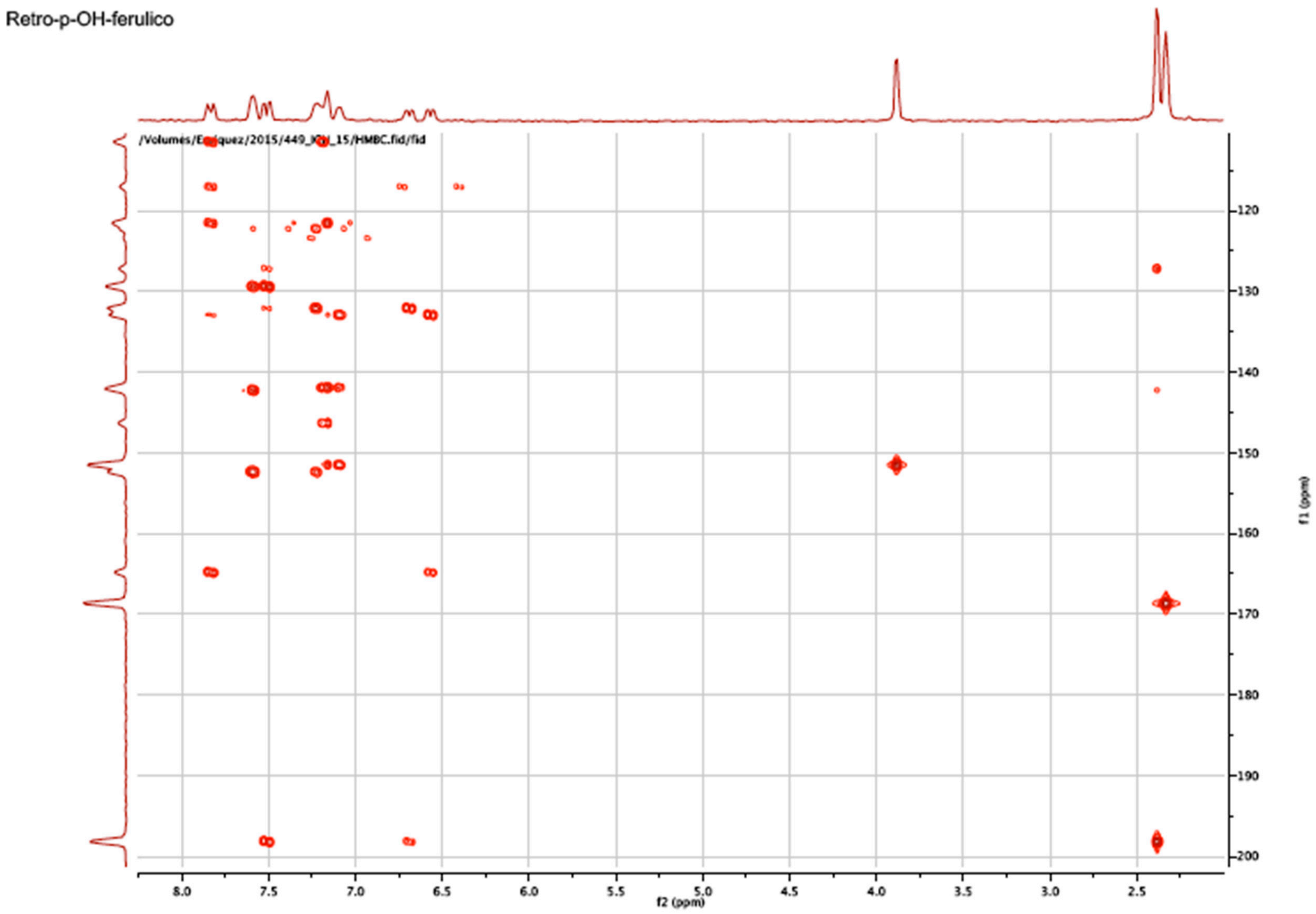

NMR *Retro*-Curcuminoid **12**

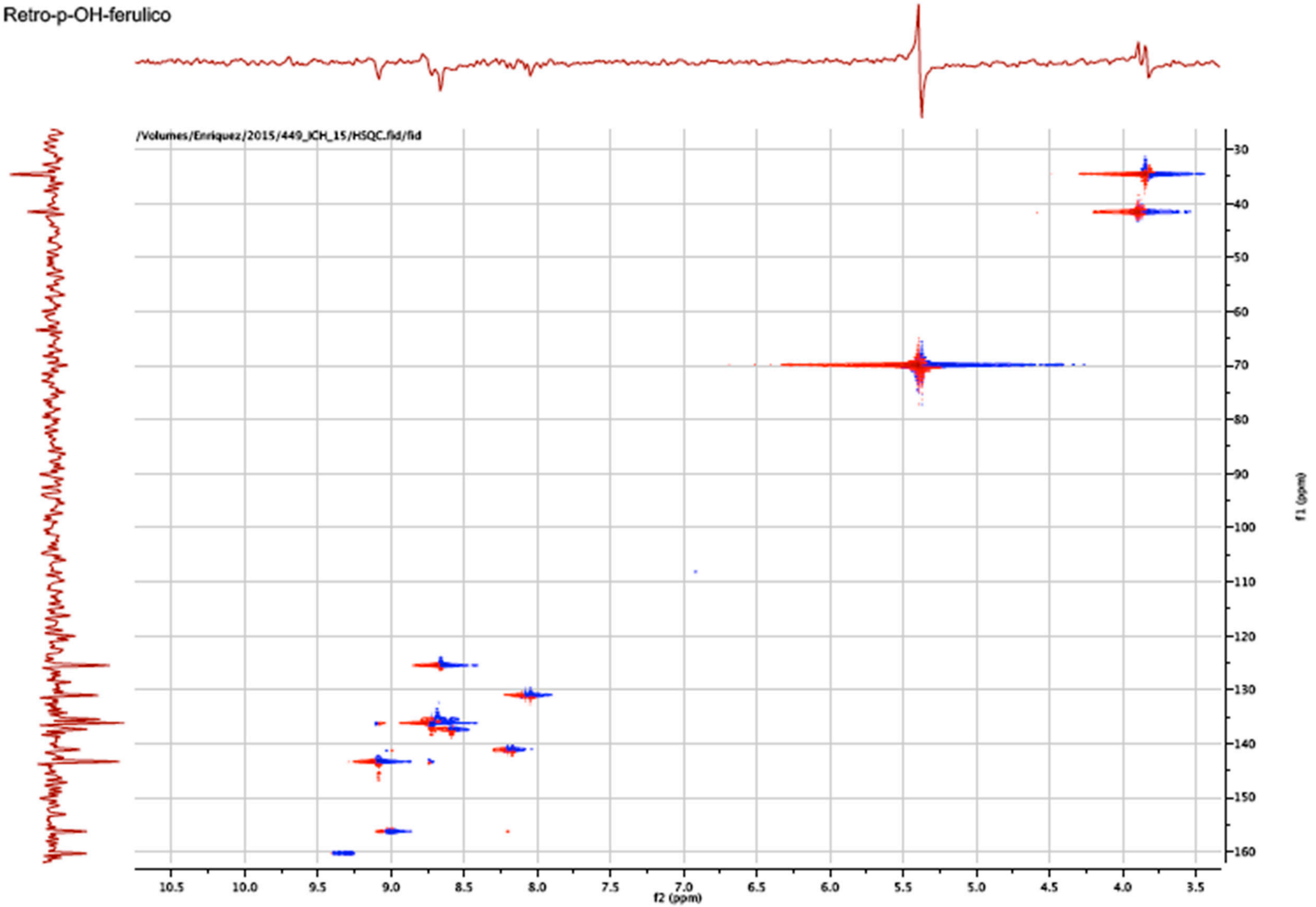

NMR *Retro*-Curcuminoid **12**

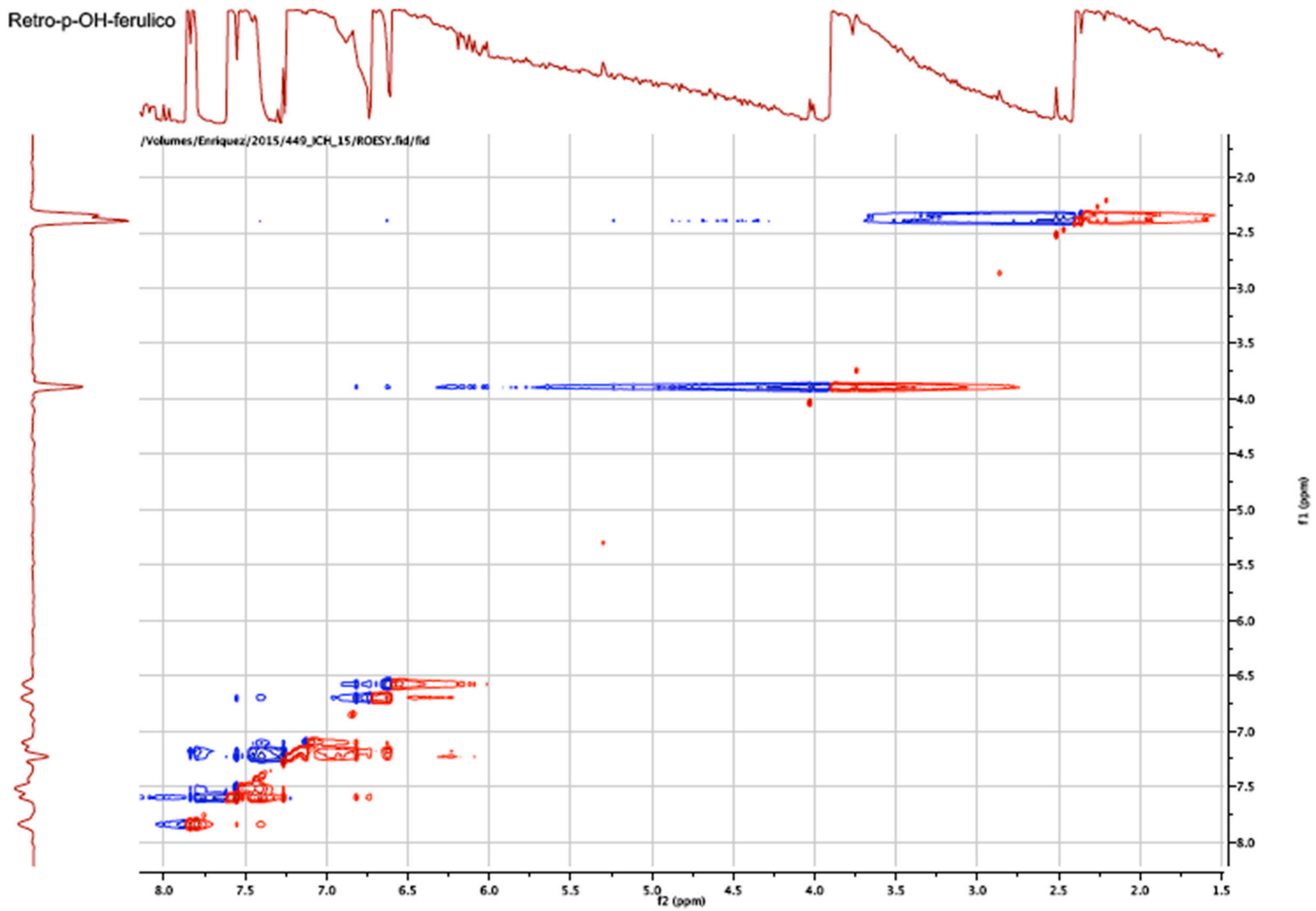

NMR *Retro*-Curcuminoid **13**

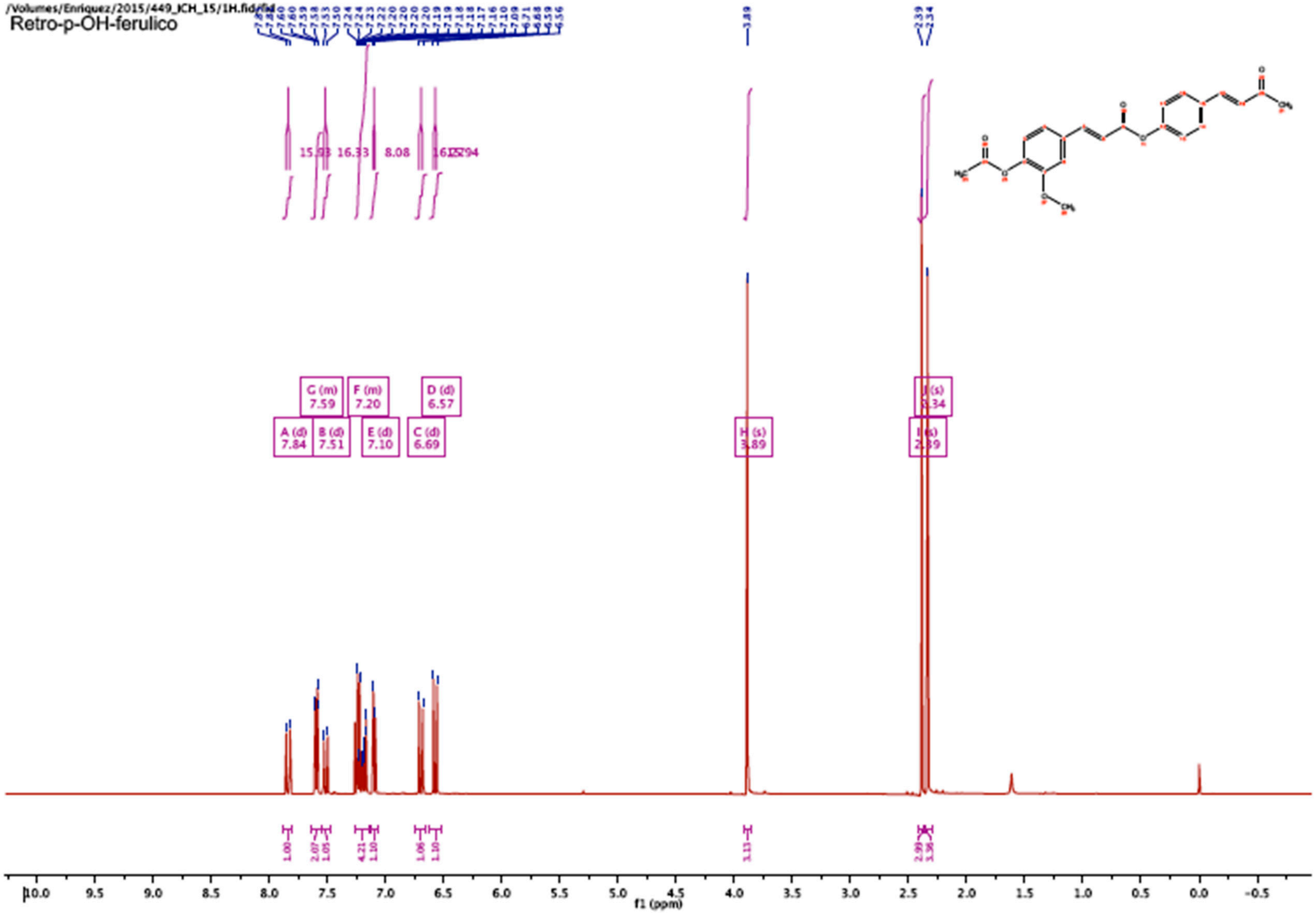

NMR *Retro*-Curcuminoid **13**

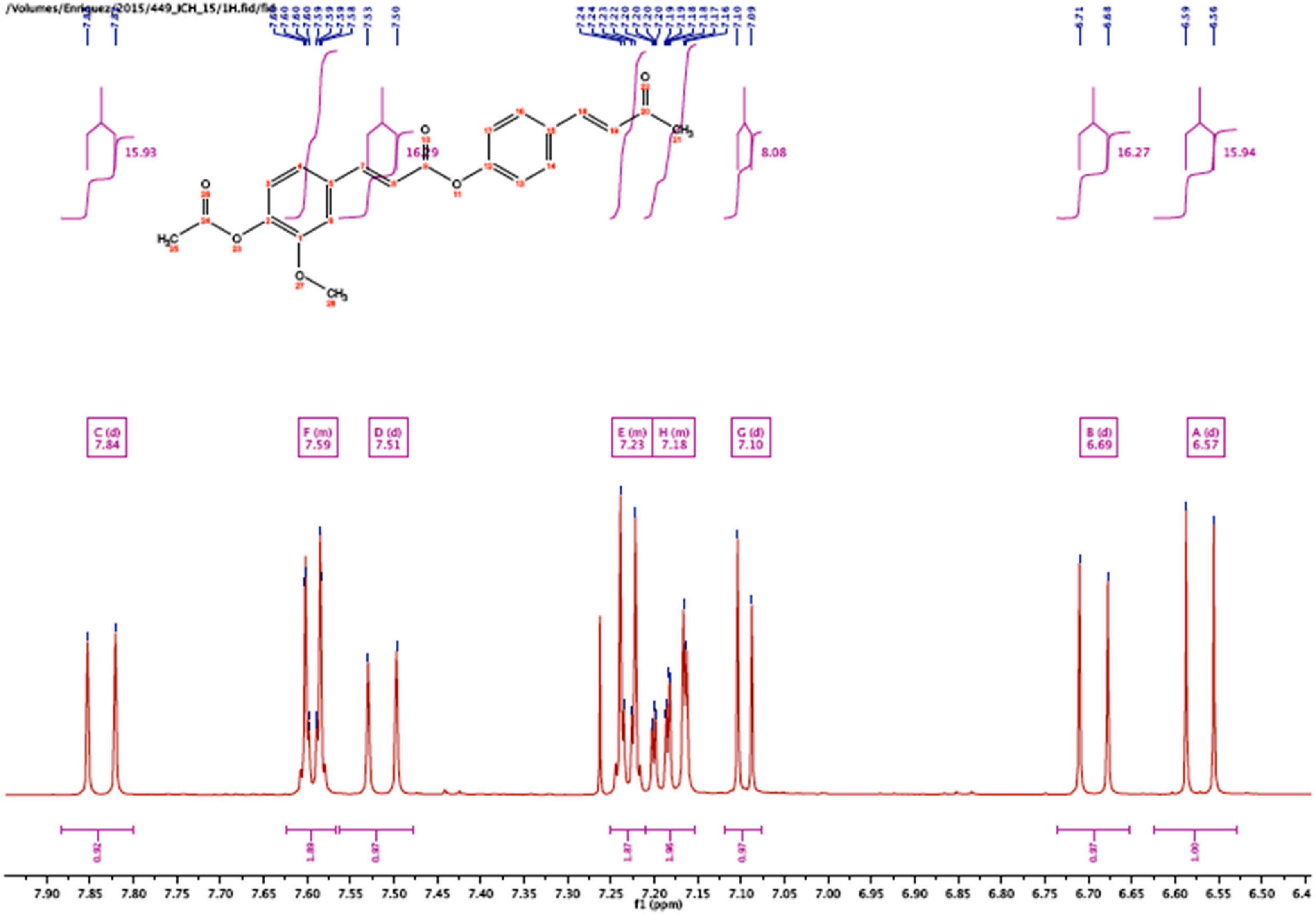

NMR *Retro*-Curcuminoid **13**

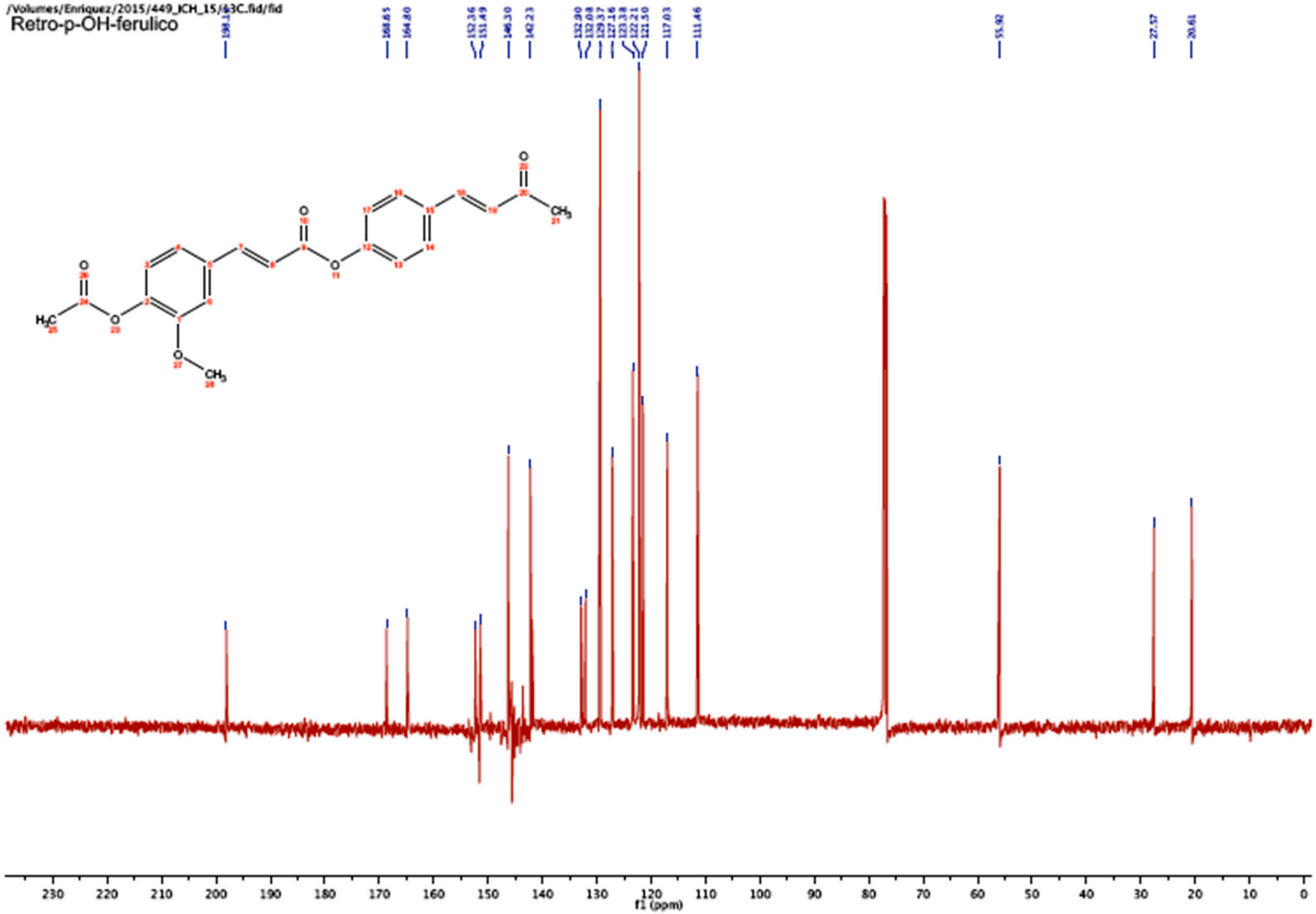

NMR *Retro*-Curcuminoid **13**

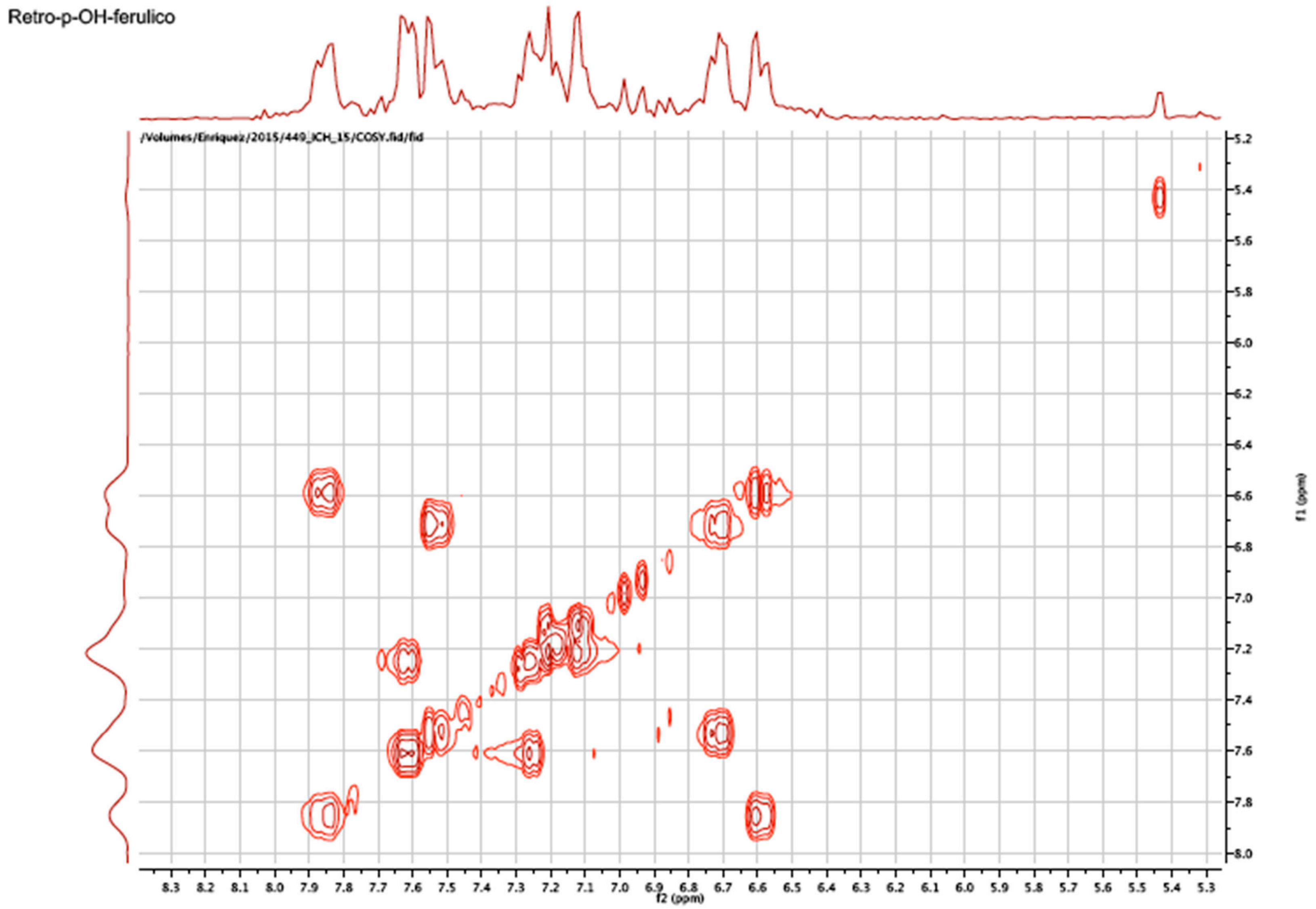

NMR *Retro*-Curcuminoid **13**

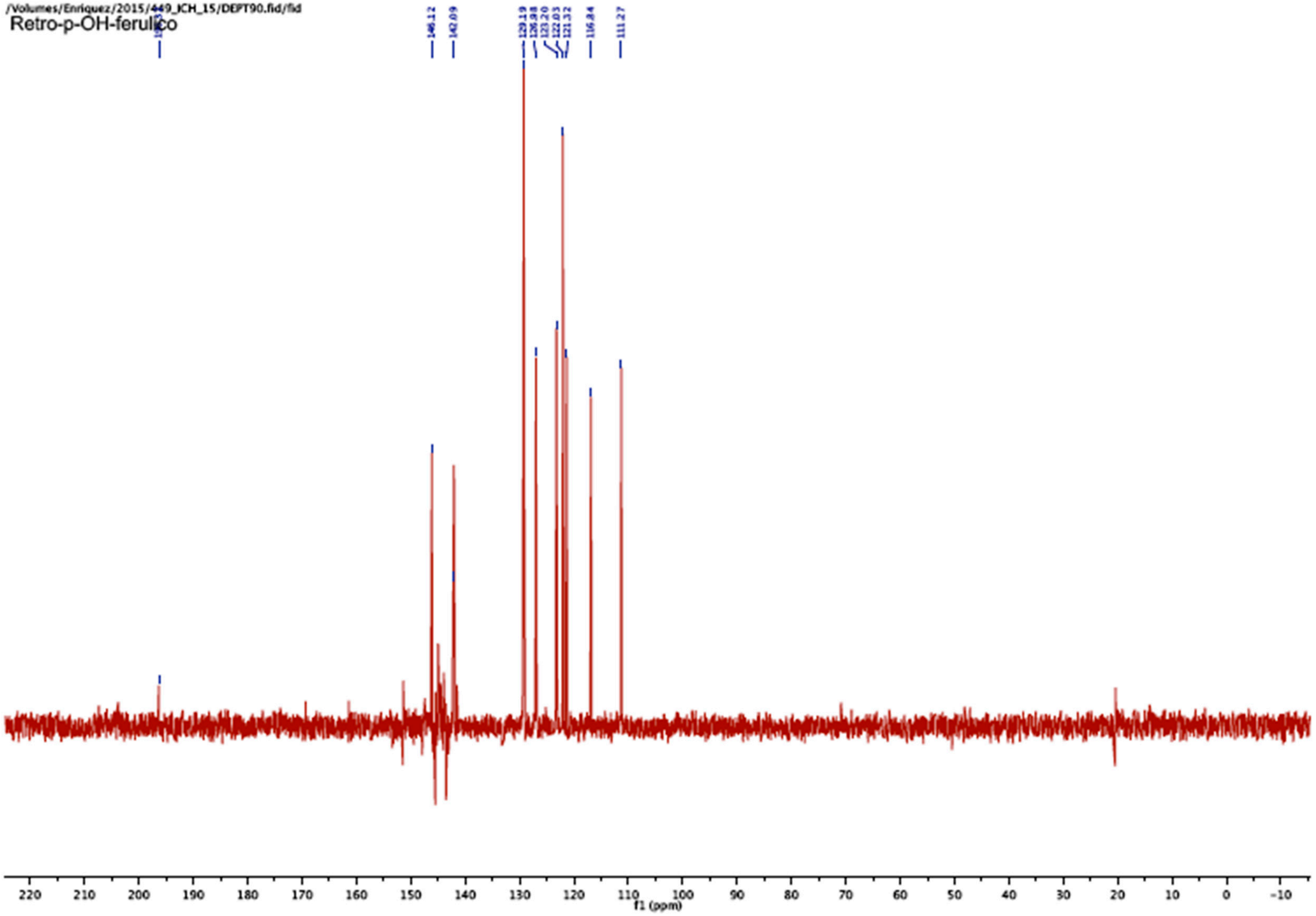

NMR *Retro*-Curcuminoid **13**

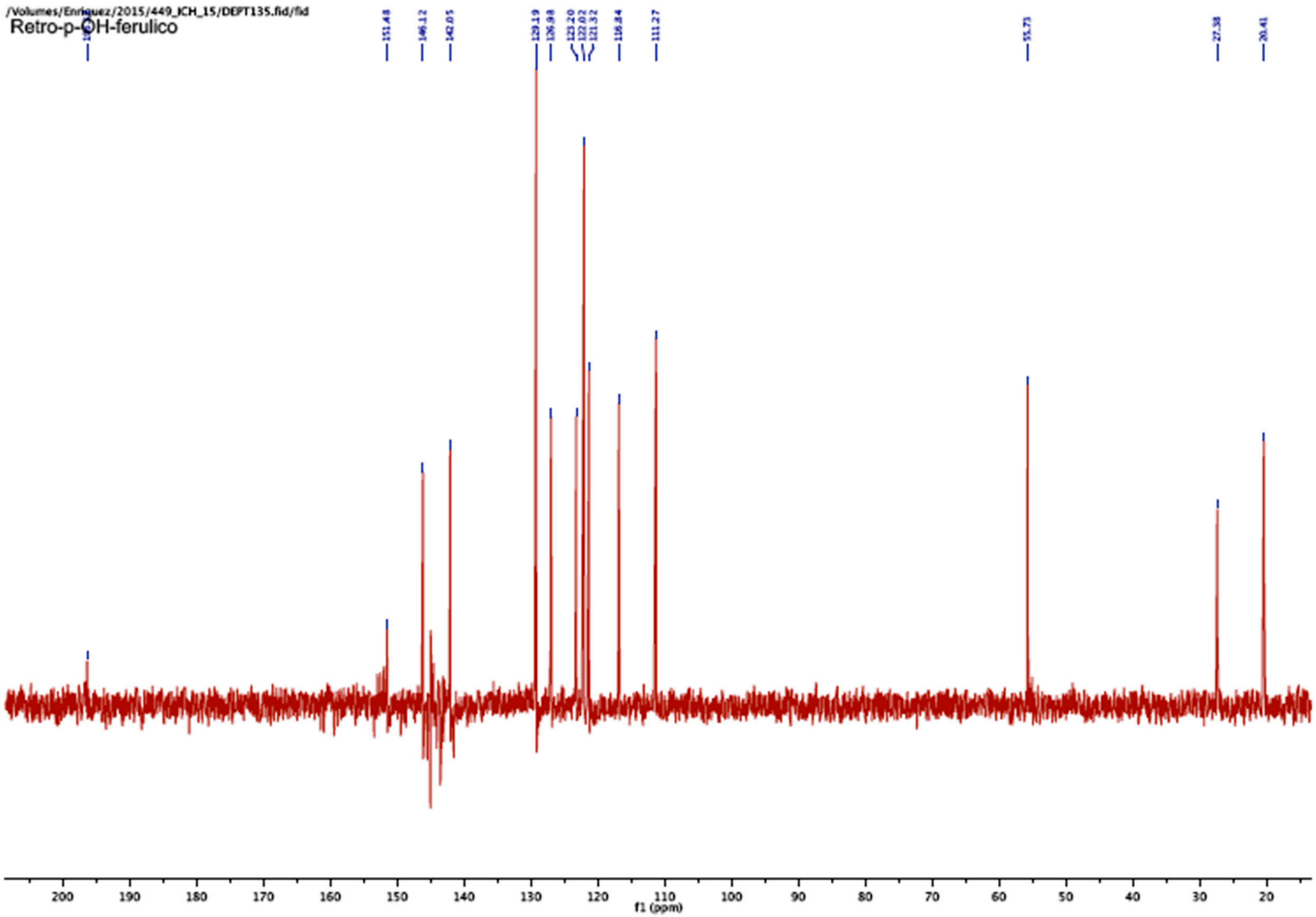

NMR *Retro*-Curcuminoid **13**

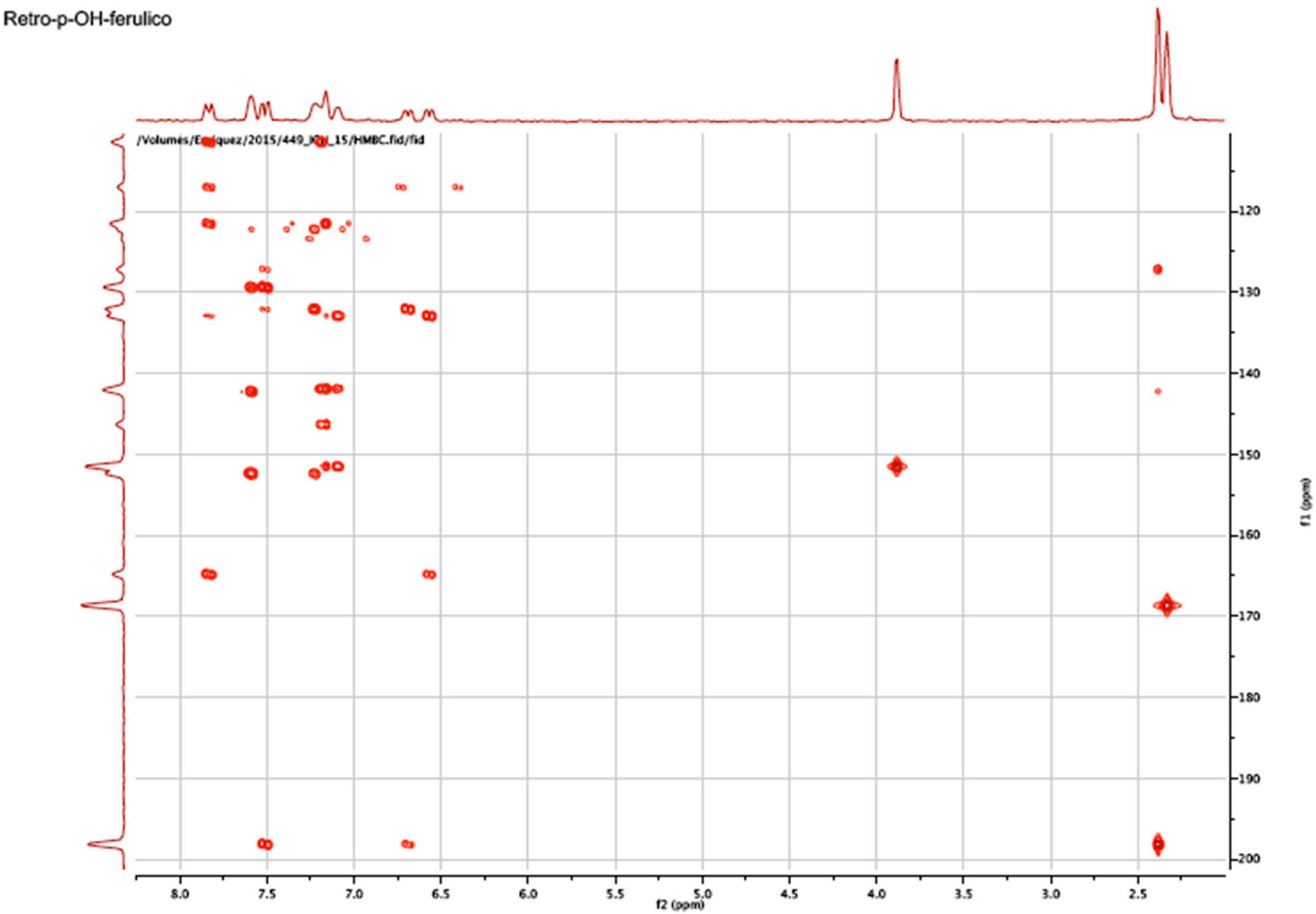

NMR *Retro*-Curcuminoid **13**

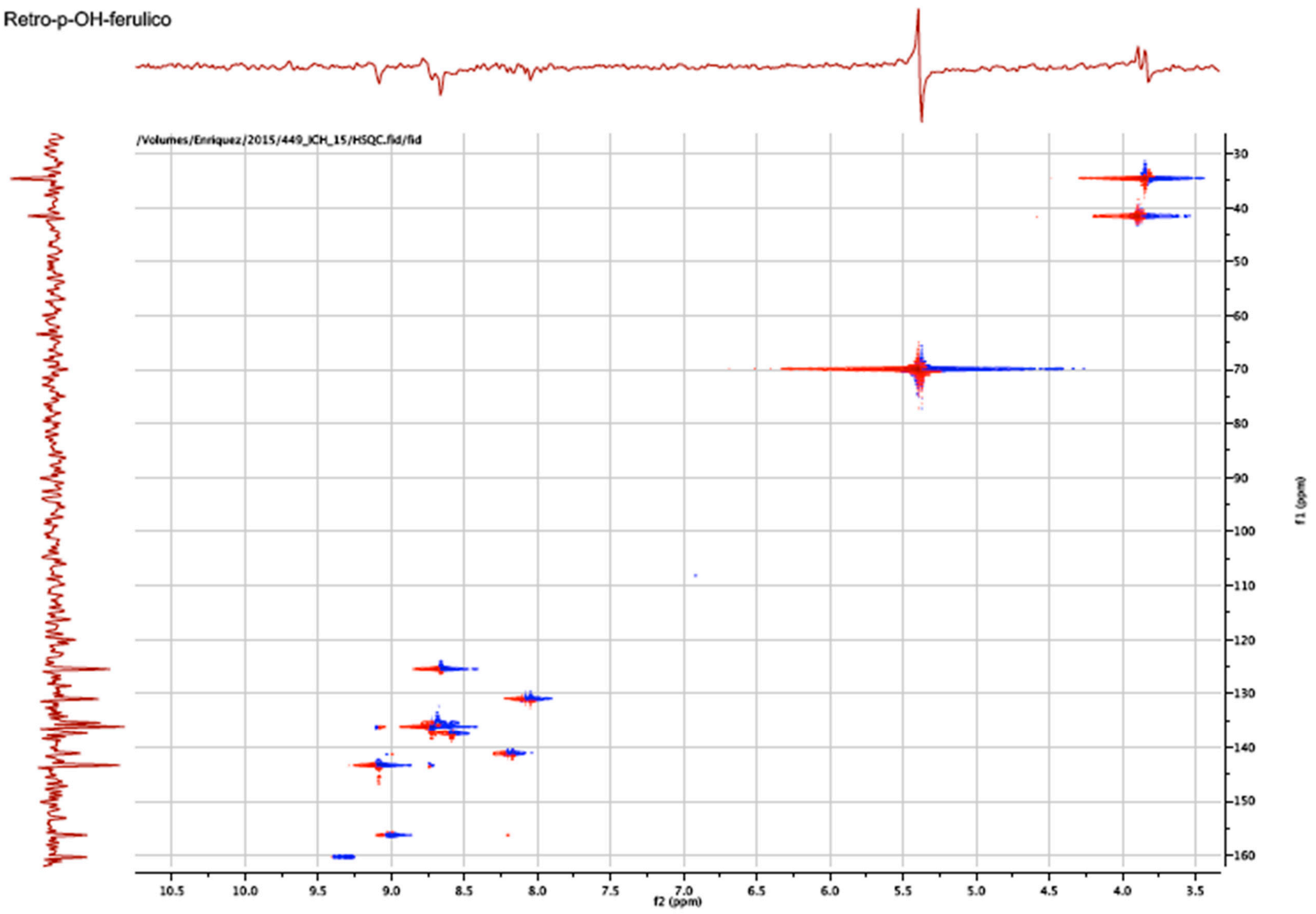

NMR *Retro*-Curcuminoid **14**

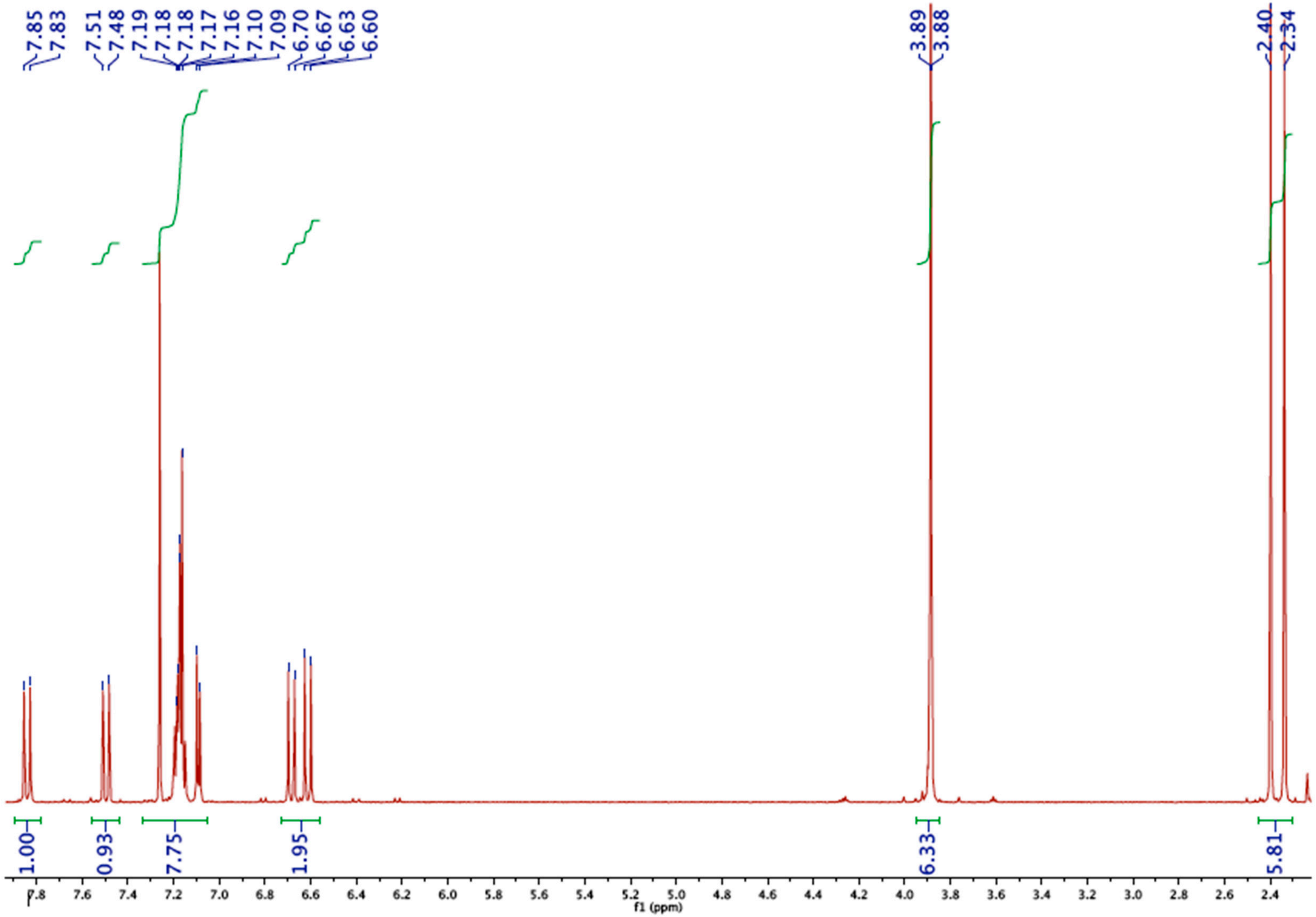

NMR *Retro*-Curcuminoid **14**

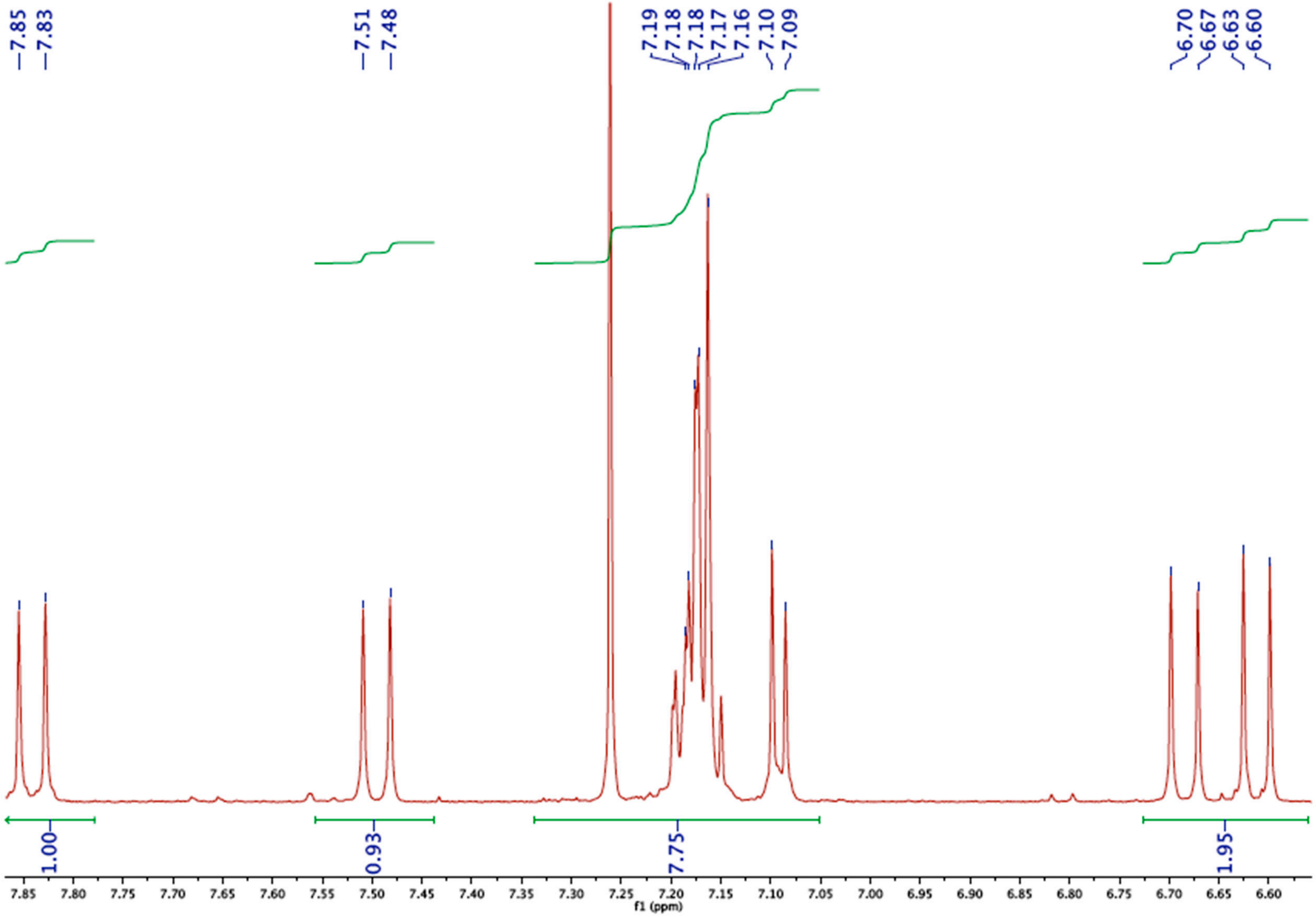

NMR *Retro*-Curcuminoid **14**

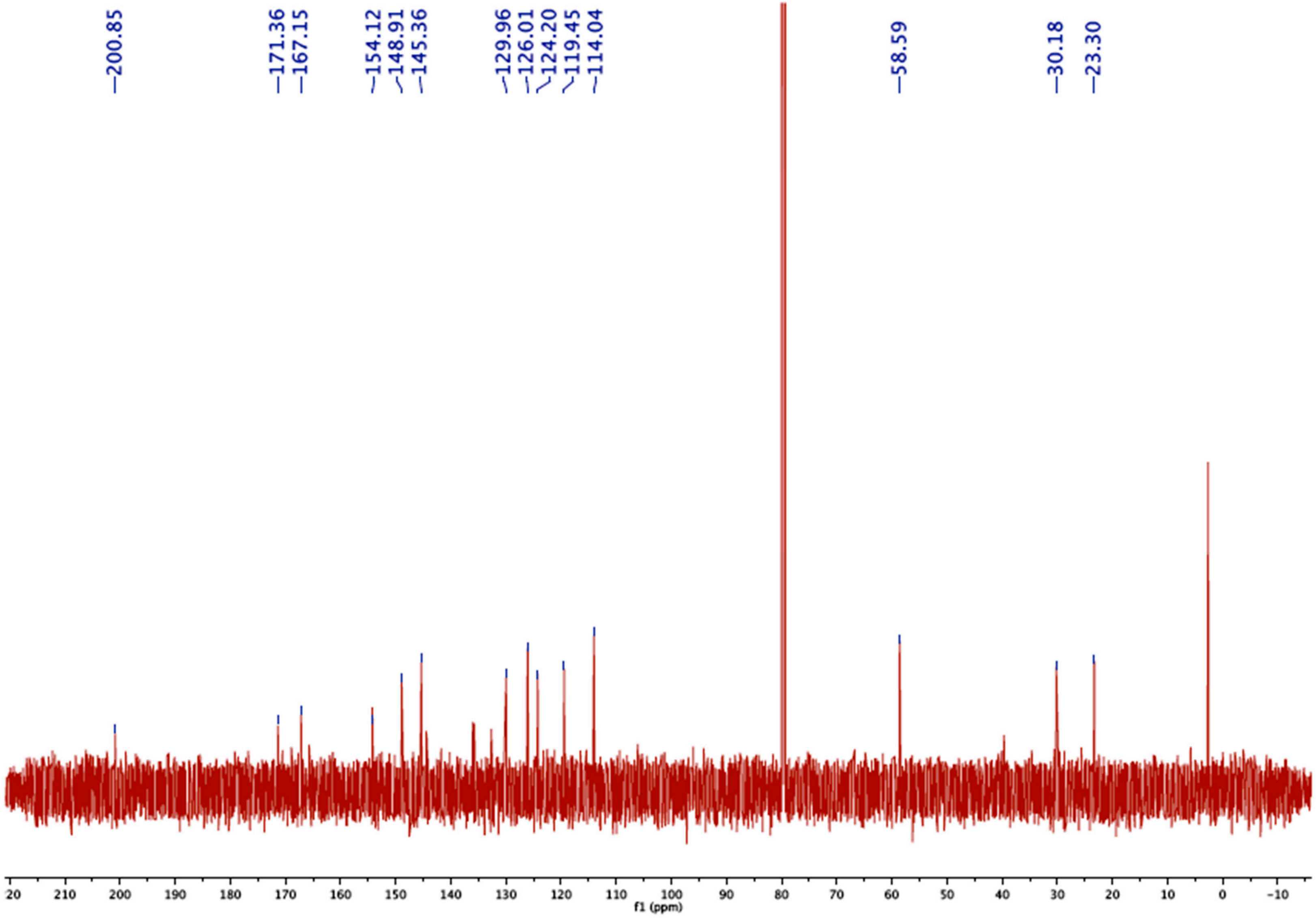

NMR *Retro*-Curcuminoid **14**

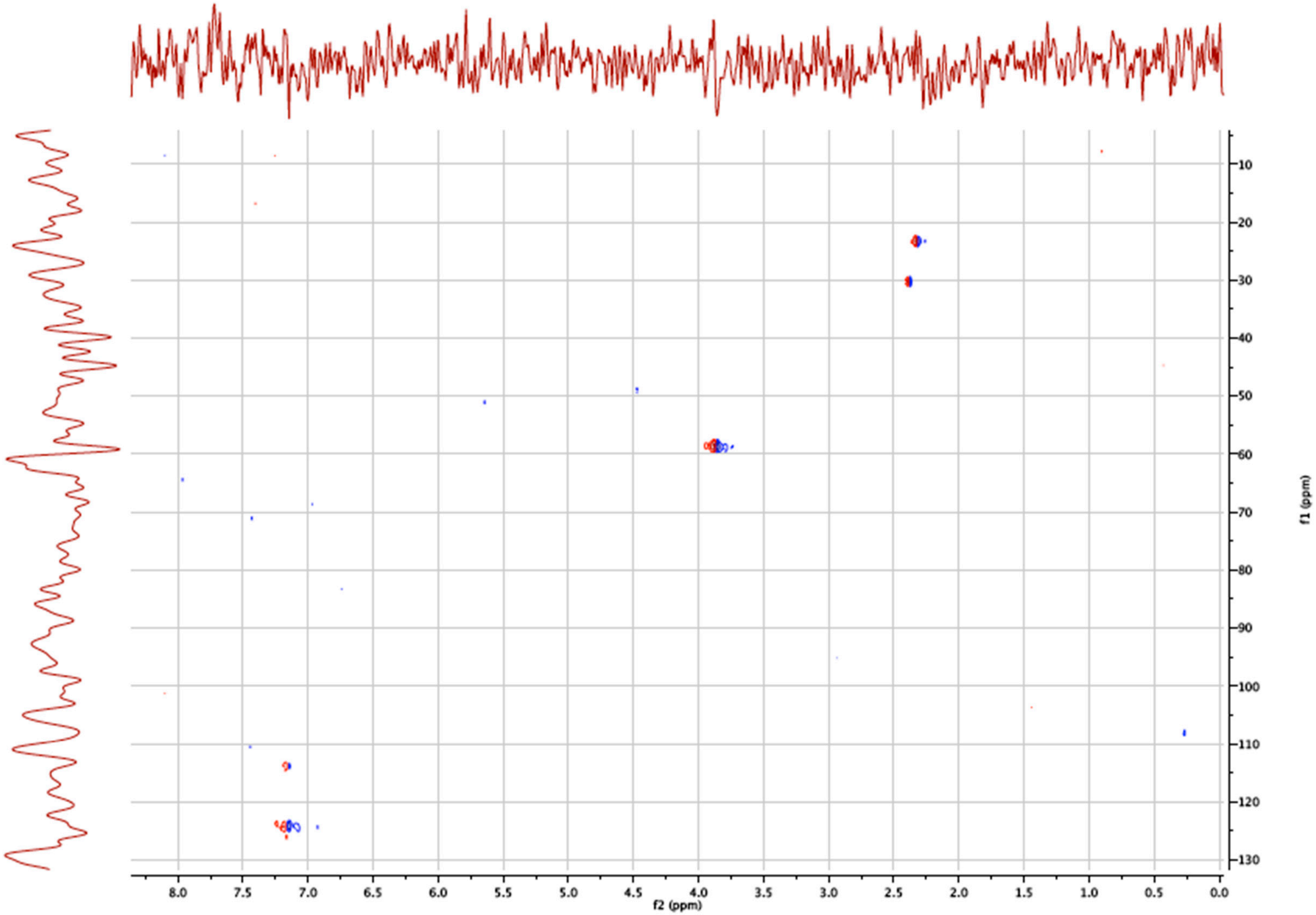

X-ray Data

Table S1. Bond distances (Å) and angles (deg) for 4-((*E*)-3-oxobut−1-en-1-yl)phenyl(*E*)-3-(4-acetoxy-3-methoxyphenyl)acrylate. Compound **4**.

O1–C1	1.187(4)	O2–C1	1.363(4)
O2–C13	1.405(3)	O3–C6	1.370(3)
O3–C10	1.426(4)	O4–C11	1.353(4)
O4–C7	1.405(3)	O5–C11	1.195(4)
O6–C21	1.214(4)	C1–C2	1.446(4)
C2–C3	1.323(4)	C3–C4	1.463(4)
C4–C9	1.385(4)	C4–C5	1.399(4)
C5–C6	1.377(4)	C6–C7	1.387(4)
C7–C8	1.367(4)	C8–C9	1.382(4)
C11–C12	1.482(4)	C13–C14	1.368(4)
C13–C18	1.372(4)	C14–C15	1.379(4)
C15–C16	1.371(4)	C16–C17	1.390(4)
C16–C19	1.480(4)	C17–C18	1.387(4)
C19–C20	1.310(4)	C20–C21	1.476(4)
C21–C22	1.489(5)		
C1–O2–C13	123.7(2)	C6–O3–C10	117.6(2)
C11–O4–C7	117.1(2)	O1–C1–O2	123.3(3)
O1–C1–C2	126.9(3)	O2–C1–C2	109.8(3)
C3–C2–C1	121.0(3)	C2–C3–C4	128.0(3)
C9–C4–C5	118.6(3)	C9–C4–C3	123.2(3)
C5–C4–C3	118.2(3)	C6–C5–C4	121.5(3)
O3–C6–C5	125.5(3)	O3–C6–C7	116.1(2)
C5–C6–C7	118.3(3)	C8–C7–C6	121.1(3)
C8–C7–O4	118.4(3)	C6–C7–O4	120.4(3)
C7–C8–C9	120.3(3)	C8–C9–C4	120.1(3)
O5–C11–O4	122.0(3)	O5–C11–C12	126.9(3)
O4–C11–C12	111.1(3)	C14–C13–C18	120.7(3)
C14–C13–O2	113.6(3)	C18–C13–O2	125.6(3)
C13–C14–C15	119.9(3)	C16–C15–C14	121.3(3)
C15–C16–C17	117.9(3)	C15–C16–C19	119.5(3)
C17–C16–C19	122.5(3)	C18–C17–C16	121.4(3)
C13–C18–C17	118.8(3)	C20–C19–C16	127.1(3)
C19–C20–C21	126.1(3)	O6–C21–C20	118.6(3)
O6–C21–C22	120.7(3)	C20–C21–C22	120.7(3)

Table S2. Bond distances (Å) and angles (deg) for 2-((*E*)-3-oxobut-1-en−1-yl)phenyl(*E*)-3-(4-acetoxy-3-methoxyphenyl)acrylate. Compound **6**.

O1–C1	1.370(2)	O1–C13	1.412(2)
O2–C1	1.200(2)	O3–C6	1.359(2)
O3–C10	1.427(2)	O4–C11	1.361(3)
O4–C7	1.400(2)	O5–C11	1.194(2)
O6–C21	1.219(3)	C1–C2	1.458(3)
C2–C3	1.320(3)	C3–C4	1.472(2)
C4–C9	1.394(3)	C4–C5	1.400(3)
C5–C6	1.386(3)	C6–C7	1.388(3)
C7–C8	1.371(3)	C8–C9	1.383(3)
C11–C12	1.488(3)	C13–C18	1.371(3)
C13–C14	1.392(3)	C14–C15	1.412(3)
C14–C19	1.455(3)	C15–C16	1.372(3)
C16–C17	1.387(3)	C17–C18	1.389(3)
C19–C20	1.325(3)	C20–C21	1.460(3)
C21–C22	1.473(3)		
C1–O1–C13	117.08(15)	C6–O3–C10	116.79(15)
C11–O4–C7	116.86(15)	O2–C1–O1	122.41(18)
O2–C1–C2	126.9(2)	O1–C1–C2	110.67(18)
C3–C2–C1	120.54(19)	C2–C3–C4	127.39(19)
C9–C4–C5	119.47(17)	C9–C4–C3	122.02(18)
C5–C4–C3	118.51(17)	C6–C5–C4	120.51(18)
O3–C6–C5	125.87(18)	O3–C6–C7	115.51(17)
C5–C6–C7	118.62(19)	C8–C7–C6	121.54(18)
C8–C7–O4	119.13(17)	C6–C7–O4	119.12(18)
C7–C8–C9	120.06(19)	C8–C9–C4	119.74(19)
O5–C11–O4	122.75(19)	O5–C11–C12	126.6(2)
O4–C11–C12	110.63(18)	C18–C13–C14	122.88(19)
C18–C13–O1	117.89(18)	C14–C13–O1	119.15(19)
C13–C14–C15	116.9(2)	C13–C14–C19	120.83(18)
C15–C14–C19	122.2(2)	C16–C15–C14	120.8(2)
C15–C16–C17	120.4(2)	C16–C17–C18	120.1(2)
C13–C18–C17	118.8(2)	C20–C19–C14	126.8(2)
C19–C20–C21	122.5(2)	O6–C21–C20	121.7(2)
O6–C21–C22	119.6(3)	C20–C21–C22	118.6(2)

Table S3. Bond distances (Å) and angles (deg) for 2-methoxy-4-((*E*)-3-oxobut-1-en-1-yl)phenyl cinnamate. Compound **7**.

O1–C2	1.227(3)	O2–C7	1.361(3)
O2–C11	1.424(4)	O3–C12	1.348(3)
O3–C8	1.402(3)	O4–C12	1.185(3)
C1–C2	1.498(4)	C2–C3	1.457(4)
C3–C4	1.319(4)	C4–C5	1.484(4)
C5–C10	1.389(4)	C5–C6	1.390(3)
C6–C7	1.388(3)	C7–C8	1.381(4)
C8–C9	1.371(4)	C9–C10	1.393(4)
C12–C13	1.474(3)	C13–C14	1.310(3)
C14–C15	1.467(3)	C15–C16	1.378(3)
C15–C20	1.385(3)	C16–C17	1.383(4)
C17–C18	1.366(4)	C18–C19	1.363(4)
C19–C20	1.375(4)		
C7–O2–C11	117.3(2)	C12–O3–C8	118.5(2)
O1–C2–C3	122.2(3)	O1–C2–C1	120.9(2)
C3–C2–C1	116.8(3)	C4–C3–C2	120.7(3)
C3–C4–C5	127.9(3)	C10–C5–C6	118.5(2)
C10–C5–C4	124.2(2)	C6–C5–C4	117.3(2)
C7–C6–C5	121.2(2)	O2–C7–C8	116.2(2)
O2–C7–C6	124.6(2)	C8–C7–C6	119.2(2)
C9–C8–C7	120.7(2)	C9–C8–O3	120.0(2)
C7–C8–O3	119.1(2)	C8–C9–C10	120.0(3)
C5–C10–C9	120.5(2)	O4–C12–O3	122.7(2)
O4–C12–C13	127.6(2)	O3–C12–C13	109.7(2)
C14–C13–C12	122.2(3)	C13–C14–C15	127.1(2)
C16–C15–C20	117.8(2)	C16–C15–C14	122.8(2)
C20–C15–C14	119.3(2)	C15–C16–C17	120.8(2)
C18–C17–C16	120.3(3)	C19–C18–C17	119.6(2)
C18–C19–C20	120.4(2)	C19–C20–C15	121.0(2)

Table S4. Bond distances (Å) and angles (deg) for 4-((*E*)-3-oxobut-1-en-1-yl)phenyl cinnamate. Compound **8**.

O1–C1	1.3587(19)	O1–C10	1.3988(18)
O2–C1	1.1963(19)	C1–C2	1.460(2)
C2–C3	1.319(2)	C3–C4	1.460(2)
C4–C5	1.386(2)	C4–C9	1.387(2)
C5–C6	1.370(2)	C6–C7	1.360(3)
C7–C8	1.377(3)	C8–C9	1.371(2)
C10–C15	1.366(2)	C10–C11	1.374(2)
C11–C12	1.378(3)	C12–C13	1.388(3)
C13–C14	1.383(3)	C13–C16A	1.446(6)
C13–C16	1.519(4)	C14–C15	1.371(2)
C16–C17	1.326(4)	C17–C18	1.456(4)
C18–O3	1.209(4)	C18–C19	1.455(7)
C16A–C17A	1.338(6)	C17A-C18A	1.443(6)
C18A–O3A	1.245(7)	C18A–C19A	1.428(8)
C1–O1–C10	118.87(12)	O2–C1–O1	122.75(15)
O2–C1–C2	126.98(15)	O1–C1–C2	110.25(14)
C3–C2–C1	121.47(16)	C2–C3–C4	127.83(16)
C5–C4–C9	117.78(16)	C5–C4–C3	119.48(15)
C9–C4–C3	122.73(15)	C6–C5–C4	121.23(18)
C7–C6–C5	120.34(18)	C6–C7–C8	119.61(17)
C9–C8–C7	120.41(18)	C8–C9–C4	120.64(17)
C15–C10–C11	121.17(15)	C15–C10–O1	117.20(14)
C11–C10–O1	121.51(15)	C10–C11–C12	118.72(18)
C11–C12–C13	121.46(16)	C14–C13–C12	117.77(16)
C14–C13–C16A	137.7(3)	C12–C13–C16A	104.5(3)
C14–C13–C16	111.4(2)	C12–C13–C16	130.8(2)
C15–C14–C13	121.37(17)	C10–C15–C14	119.49(15)
C17–C16–C13	123.3(4)	C16–C17–C18	124.8(3)
O3–C18–C19	119.3(4)	O3–C18–C17	118.3(4)
C19–C18–C17	122.2(4)	C17A–C16A–C13	117.7(6)
C16A–C17A–C18A	125.4(5)	O3A–C18A–C19A	120.0(6)
O3A–C18A–C17A	118.4(6)	C19A–C18A–C17A	121.5(6)

Table S5. Bond distances (Å) and angles (deg) for 3-((*E*)-3-oxobut-1-en-1-yl)phenyl cinnamate. Compound **9**.

O1–C1	1.360(2)	O1–C10	1.415(2)
O2–C1	1.195(2)	O3–C18	1.209(3)
C1–C2	1.461(3)	C2–C3	1.323(3)
C3–C4	1.465(3)	C4–C9	1.385(3)
C4–C5	1.388(3)	C5–C6	1.381(3)
C6–C7	1.368(3)	C7–C8	1.377(3)
C8–C9	1.377(3)	C10–C15	1.368(3)
C10–C11	1.377(3)	C11–C12	1.399(3)
C12–C13	1.386(3)	C12–C16	1.466(3)
C13–C14	1.378(3)	C14–C15	1.380(3)
C16–C17	1.310(3)	C17–C18	1.475(3)
C18–C19	1.486(3)		
C1–O1–C10	118.89(15)	O2–C1–O1	123.75(18)
O2–C1–C2	125.65(19)	O1–C1–C2	110.60(17)
C3–C2–C1	119.79(19)	C2–C3–C4	127.37(19)
C9–C4–C5	118.36(19)	C9–C4–C3	121.85(18)
C5–C4–C3	119.78(18)	C6–C5–C4	120.7(2)
C7–C6–C5	120.0(2)	C6–C7–C8	120.1(2)
C9–C8–C7	120.0(2)	C8–C9–C4	120.8(2)
C15–C10–C11	122.46(18)	C15–C10–O1	122.05(17)
C11–C10–O1	115.45(17)	C10–C11–C12	119.37(18)
C13–C12–C11	118.19(18)	C13–C12–C16	119.58(18)
C11–C12–C16	122.21(18)	C14–C13–C12	121.08(19)
C13–C14–C15	120.7(2)	C10–C15–C14	118.15(19)
C17–C16–C12	126.32(19)	C16–C17–C18	126.1(2)
O3–C18–C17	118.8(2)	O3–C18–C19	121.2(2)
C17–C18–C19	119.9(2)		

Table S6. Bond distances (Å) and angles (deg) for 2-((*E*)-3 -oxobut-1-en-1-yl)phenyl cinnamate. Compound **10**.

O1–C1	1.352(6)	O1–C10	1.394(4)
O2–C1	1.188(5)	C1–C2	1.528(6)
C2–C3	1.252(5)	C3–C4	1.481(6)
C4–C9	1.372(5)	C4–C5	1.384(6)
C5–C6	1.381(5)	C6–C7	1.372(6)
C7–C8	1.372(6)	C8–C9	1.363(6)
C10–C15	1.361(5)	C10–C11	1.386(6)
C11–C12	1.385(6)	C11–C16A	1.467(19)
C11–C16	1.535(15)	C12–C13	1.380(6)
C13–C14	1.356(5)	C14–C15	1.378(5)
C16–C17	1.32(2)	C17–C18	1.495(12)
C18–O3	1.209(11)	C18–C19	1.510(13)
C16A–C17A	1.27(3)	C17A–C18A	1.496(13)
C18A–O3A	1.196(12)	C18A–C19A	1.517(13)
C1–O1–C10	117.3(4)	O2–C1–O1	124.8(6)
O2–C1–C2	126.9(6)	O1–C1–C2	108.2(5)
C3–C2–C1	122.8(5)	C2–C3–C4	127.1(5)
C9–C4–C5	118.3(5)	C9–C4–C3	118.6(5)
C5–C4–C3	123.0(5)	C6–C5–C4	120.6(5)
C7–C6–C5	119.7(5)	C8–C7–C6	120.1(5)
C9–C8–C7	119.8(5)	C8–C9–C4	121.6(5)
C15–C10–C11	123.8(5)	C15–C10–O1	116.7(5)
C11–C10–O1	119.4(5)	C10–C11–C12	115.5(5)
C10–C11–C16A	138.5(8)	C12–C11–C16A	105.8(8)
C10–C11–C16	112.0(7)	C12–C11–C16	132.2(8)
C16A-C11–C16	27.6(6)	C13–C12–C11	121.8(5)
C14–C13–C12	120.0(6)	C13–C14–C15	120.4(5)
C10–C15–C14	118.4(5)	C17–C16–C11	121.6(12)
C16–C17–C18	126.1(13)	O3–C18–C17	117.7(11)
O3–C18–C19	122.7(13)	C17–C18–C19	119.5(11)
C17A–C16A–C11	121.8(16)	C16A–C17A–C18A	118.1(16)
O3A–C18A–C17A	122.6(13)	O3A–C18A–C19A	122.2(15)
C17A–C18A–C19A	115.1(12)		

Table S7. Atomic coordinates and displacement parameters (Å^2) for 4-((*E*)-3-oxobut-1-en−1-yl)phenyl(*E*)-3-(4-acetoxy-3-methoxy phenyl)acrylate. Compound **4**.

	x	y	z	U11	U22	U33	U23	U13	U12
O1	0.60250(10)	0.0651(4)	0.7022(2)	0.0479(15)	0.153(3)	0.0590(18)	−0.0314(17)	−0.0074(13)	0.0223(15)
O2	0.54118(9)	0.1386(3)	0.84428(18)	0.0337(13)	0.0884(19)	0.0492(14)	−0.0062(12)	−0.0045(11)	0.0003(11)
O3	0.90397(9)	−0.0559(3)	1.0289(2)	0.0420(14)	0.0757(19)	0.0542(15)	−0.0079(12)	−0.0037(11)	0.0159(11)
O4	0.89138(9)	0.0710(3)	1.25598(18)	0.0456(13)	0.0682(17)	0.0440(13)	0.0090(11)	−0.0112(10)	−0.0084(11)
O5	0.93925(10)	0.2830(3)	1.1666(2)	0.0607(16)	0.076(2)	0.0738(18)	0.0109(15)	−0.0091(13)	−0.0173(13)
O6	0.22473(10)	0.1608(4)	0.3101(2)	0.0649(16)	0.102(2)	0.0498(16)	−0.0077(14)	−0.0082(12)	0.0068(14)
C1	0.59558(14)	0.1080(4)	0.8044(3)	0.036(2)	0.066(3)	0.052(2)	−0.0026(18)	−0.0053(17)	0.0054(16)
C2	0.64131(13)	0.1338(4)	0.9045(3)	0.041(2)	0.058(2)	0.0424(19)	0.0001(16)	−0.0008(15)	−0.0013(16)
C3	0.69624(13)	0.0790(4)	0.8962(3)	0.0388(19)	0.051(2)	0.0446(19)	0.0009(15)	−0.0004(15)	−0.0041(15)
C4	0.74681(12)	0.0876(4)	0.9899(3)	0.0381(18)	0.041(2)	0.0406(19)	0.0065(15)	0.0006(14)	−0.0014(14)
C5	0.80078(12)	0.0156(4)	0.9622(3)	0.0432(19)	0.044(2)	0.0393(18)	0.0007(15)	−0.0015(15)	−0.0009(15)
C6	0.84973(13)	0.0136(4)	1.0472(3)	0.0377(19)	0.048(2)	0.044(2)	0.0000(15)	−0.0022(15)	0.0011(15)
C7	0.84424(13)	0.0832(4)	1.1630(3)	0.0383(19)	0.045(2)	0.044(2)	0.0065(15)	−0.0104(15)	−0.0044(15)
C8	0.79223(13)	0.1570(4)	1.1913(3)	0.047(2)	0.050(2)	0.0402(18)	−0.0036(15)	−0.0002(16)	−0.0046(16)
C9	0.74338(13)	0.1598(4)	1.1054(3)	0.0377(18)	0.047(2)	0.050(2)	−0.0016(16)	0.0032(15)	−0.0018(15)
C10	0.91421(14)	−0.1036(5)	0.9062(3)	0.052(2)	0.082(3)	0.064(3)	0.007(2)	0.0133(18)	0.0093(19)
C11	0.93775(14)	0.1805(5)	1.2486(3)	0.044(2)	0.053(3)	0.055(2)	−0.0092(19)	−0.0040(17)	0.0018(18)
C12	0.98328(14)	0.1552(5)	1.3538(3)	0.059(2)	0.087(3)	0.062(2)	−0.017(2)	−0.0236(19)	0.009(2)
C13	0.48748(13)	0.1179(4)	0.7701(3)	0.0314(18)	0.057(2)	0.045(2)	−0.0011(16)	−0.0095(15)	0.0000(15)
C14	0.44018(13)	0.0811(4)	0.8353(3)	0.041(2)	0.078(3)	0.044(2)	0.0011(17)	0.0028(16)	0.0015(17)
C15	0.38385(14)	0.0719(5)	0.7754(3)	0.041(2)	0.078(3)	0.052(2)	0.0018(18)	0.0060(17)	−0.0024(17)
C16	0.37415(13)	0.0955(4)	0.6506(3)	0.0352(18)	0.047(2)	0.053(2)	−0.0024(16)	−0.0029(16)	0.0004(14)
C17	0.42307(14)	0.1266(5)	0.5855(3)	0.048(2)	0.087(3)	0.042(2)	0.0055(18)	−0.0021(17)	−0.0009(18)
C18	0.47984(14)	0.1398(5)	0.6448(3)	0.036(2)	0.099(3)	0.052(2)	0.011(2)	−0.0007(17)	−0.0079(18)
C19	0.31273(13)	0.0915(4)	0.5908(3)	0.042(2)	0.054(2)	0.055(2)	−0.0037(17)	0.0010(16)	−0.0029(16)
C20	0.29584(14)	0.1175(4)	0.4741(3)	0.045(2)	0.065(3)	0.052(2)	−0.0024(17)	0.0021(17)	0.0035(16)
C21	0.23411(13)	0.1164(4)	0.4170(3)	0.044(2)	0.056(2)	0.048(2)	−0.0095(17)	−0.0057(17)	0.0085(16)
C22	0.18461(14)	0.0576(5)	0.4879(3)	0.046(2)	0.067(3)	0.074(3)	−0.0063(19)	−0.0012(19)	0.0053(17)

Table S8. Atomic coordinates and displacement parameters (Å^2) for 2-((*E*)-3-oxobut-1-en-1-yl)phenyl(*E*)-3-(4-acetoxy-3-methoxyphenyl)acrylate Compound **6**.

	x	y	z	U11	U22	U33	U23	U13	U12
O1	0.8087(2)	1.14704(8)	0.21564(8)	0.0337(8)	0.0428(9)	0.0381(9)	−0.0123(7)	−0.0022(7)	−0.0081(6)
O2	1.0179(2)	1.10146(8)	0.12438(9)	0.0414(9)	0.0458(10)	0.0548(11)	−0.0166(8)	0.0099(8)	−0.0136(7)
O3	0.4285(2)	0.76878(8)	−0.04855(8)	0.0396(8)	0.0480(9)	0.0365(9)	−0.0138(8)	0.0064(7)	−0.0144(7)
O4	0.0599(2)	0.75979(8)	0.02750(8)	0.0302(7)	0.0364(9)	0.0405(9)	0.0019(7)	−0.0048(7)	−0.0099(6)
O5	0.2711(3)	0.66795(9)	0.07869(10)	0.0668(10)	0.0440(10)	0.0697(13)	0.0152(9)	−0.0306(10)	−0.0112(8)
O6	1.1927(3)	0.95602(12)	0.36514(13)	0.0908(14)	0.0686(14)	0.122(2)	0.0332(13)	−0.0379(14)	−0.0169(12)
C1	0.8521(3)	1.09764(12)	0.15923(12)	0.0328(11)	0.0332(12)	0.0317(13)	−0.0009(10)	−0.0054(10)	−0.0011(9)
C2	0.6731(3)	1.04253(11)	0.14947(11)	0.0303(10)	0.0346(12)	0.0311(13)	0.0019(10)	−0.0017(9)	−0.0048(9)
C3	0.5117(3)	0.93235(11)	0.07856(11)	0.0294(10)	0.0306(11)	0.0334(13)	0.0054(10)	−0.0029(9)	−0.0003(9)
C4	0.5117(3)	0.93235(11)	0.07856(11)	0.0269(10)	0.0282(11)	0.0277(12)	0.0045(9)	−0.0048(9)	−0.0020(8)
C5	0.5553(3)	0.87992(11)	0.02240(11)	0.0248(10)	0.0350(12)	0.0280(12)	0.0027(10)	−0.0008(9)	−0.0030(8)
C6	0.4067(3)	0.82178(11)	0.00555(11)	0.0313(10)	0.0334(12)	0.0242(12)	−0.0009(10)	−0.0026(9)	−0.0029(9)
C7	0.2159(3)	0.81603(11)	0.04618(11)	0.0241(10)	0.0328(12)	0.0304(12)	0.0025(10)	−0.0056(9)	−0.0054(8)
C8	0.1687(3)	0.86785(12)	0.10033(12)	0.0247(10)	0.0421(13)	0.0374(13)	−0.0006(11)	0.0038(9)	−0.0013(9)
C9	0.3152(3)	0.92658(11)	0.11677(12)	0.0296(10)	0.0359(12)	0.0354(13)	−0.0041(10)	0.0005(9)	−0.0002(9)
C10	0.6137(3)	0.77711(13)	−0.09509(12)	0.0341(11)	0.0506(14)	0.0364(13)	−0.0112(11)	0.0022(10)	−0.0011(10)
C11	0.1124(3)	0.68530(13)	0.04338(12)	0.0455(13)	0.0418(14)	0.0331(13)	0.0068(11)	−0.0036(11)	−0.0104(11)
C12	−0.0514(4)	0.63159(13)	0.01024(14)	0.0626(15)	0.0463(15)	0.0495(16)	0.0039(12)	−0.0105(13)	−0.0222(12)
C13	0.9700(3)	1.20315(12)	0.23232(11)	0.0339(11)	0.0387(13)	0.0310(13)	−0.0114(10)	0.0023(9)	−0.0074(9)
C14	1.1500(3)	1.18261(13)	0.27571(12)	0.0372(11)	0.0456(14)	0.0285(12)	−0.0061(11)	0.0008(10)	−0.0069(10)
C15	1.3007(3)	1.24172(14)	0.29390(12)	0.0406(12)	0.0546(15)	0.0292(13)	−0.0109(12)	−0.0021(10)	−0.0068(11)
C16	1.2691(4)	1.31562(13)	0.26917(12)	0.0456(13)	0.0457(15)	0.0390(14)	−0.0168(12)	0.0025(11)	−0.0108(11)
C17	1.0865(3)	1.33378(13)	0.22653(13)	0.0508(13)	0.0384(13)	0.0450(15)	−0.0112(11)	0.0068(12)	−0.0041(11)
C18	0.9357(3)	1.27684(12)	0.20760(13)	0.0391(12)	0.0435(14)	0.0401(14)	−0.0096(12)	0.0008(10)	−0.0011(10)
C19	1.1749(3)	1.10422(13)	0.30289(12)	0.0423(12)	0.0520(15)	0.0352(14)	−0.0034(12)	−0.0046(11)	−0.0101(11)
C20	1.3557(4)	1.07276(14)	0.33088(13)	0.0405(12)	0.0527(15)	0.0467(15)	−0.0085(12)	−0.0050(11)	−0.0014(11)
C21	1.3586(4)	0.99539(15)	0.36229(15)	0.0619(16)	0.0517(16)	0.0623(18)	−0.0091(14)	−0.0154(14)	0.0012(14)
C22	1.5648(5)	0.96676(18)	0.3955(2)	0.083(2)	0.070(2)	0.130(3)	−0.007(2)	−0.036(2)	0.0279(17)

Table S9. Atomic coordinates and displacement parameters (Å^2) for 2-methoxy-4-((*E*)-3-oxobut-1-en-1-yl)phenyl cinnamate. Compound **7**.

	x	y	z	U11	U22	U33	U23	U13	U12
O1	0.2323(3)	0.5569(2)	−0.17913(18)	0.0728(13)	0.1141(16)	0.0735(13)	−0.0331(11)	−0.0242(10)	0.0197(12)
O2	−0.0736(3)	0.2035(2)	0.50660(15)	0.0700(12)	0.1016(14)	0.0464(10)	−0.0216(9)	−0.0124(8)	0.0164(10)
O3	0.1935(3)	0.05867(18)	0.55160(15)	0.0945(14)	0.0678(11)	0.0549(10)	−0.0132(8)	−0.0341(10)	0.0060(9)
O4	0.3093(3)	0.2342(2)	0.58885(18)	0.1180(18)	0.0912(15)	0.0639(12)	−0.0161(10)	−0.0306(12)	−0.0233(13)
C1	0.4913(4)	0.4753(3)	−0.2915(3)	0.087(2)	0.095(2)	0.0656(17)	−0.0306(15)	−0.0171(15)	0.0176(17)
C2	0.3551(4)	0.4832(3)	−0.1741(2)	0.0703(18)	0.0622(15)	0.0617(15)	−0.0192(12)	−0.0245(13)	−0.0006(13)
C3	0.3750(4)	0.3988(3)	−0.0553(2)	0.0716(18)	0.0733(17)	0.0649(16)	−0.0244(13)	−0.0200(14)	0.0115(13)
C4	0.2467(4)	0.3818(3)	0.0526(2)	0.0630(16)	0.0687(15)	0.0630(15)	−0.0270(12)	−0.0188(12)	0.0023(12)
C5	0.2440(4)	0.2996(3)	0.1813(2)	0.0699(17)	0.0596(14)	0.0511(13)	−0.0182(11)	−0.0162(12)	0.0070(12)
C6	0.0893(4)	0.2927(3)	0.2810(2)	0.0676(16)	0.0638(14)	0.0516(14)	−0.0202(11)	−0.0178(12)	0.0143(12)
C7	0.0741(3)	0.2166(3)	0.4044(2)	0.0640(15)	0.0629(14)	0.0457(13)	−0.0186(11)	−0.0131(11)	0.0060(12)
C8	0.2153(4)	0.1461(3)	0.4282(2)	0.0709(17)	0.0592(14)	0.0484(13)	−0.0154(11)	−0.0220(12)	0.0060(12)
C9	0.3696(4)	0.1521(3)	0.3316(2)	0.0705(17)	0.0731(16)	0.0688(17)	−0.0258(13)	−0.0242(14)	0.0170(13)
C10	0.3849(4)	0.2293(3)	0.2079(2)	0 0.0670(17)	0.0755(17)	0.0571(15)	−0.0255(13)	−0.0083(12)	0.0086(13)
C11	−0.2245(4)	0.2692(4)	0.4842(3)	0.076(2)	0.140(3)	0.0686(18)	−0.0384(19)	−0.0179(15)	0.036(2)
C12	0.2436(3)	0.1150(3)	0.6251(2)	0.0535(15)	0.0755(17)	0.0576(15)	−0.0227(13)	−0.0173(12)	0.0092(13)
C13	0.2046(3)	0.0069(3)	0.7512(2)	0.0600(16)	0.0700(16)	0.0577(15)	−0.0191(12)	−0.0215(12)	0.0104(12)
C14	0.2474(3)	0.0320(3)	0.8396(2)	0.0566(15)	0.0699(16)	0.0588(15)	−0.0196(12)	−0.0183(12)	0.0069(12)
C15	0.2126(3)	−0.0657(3)	0.9686(2)	0.0475(13)	0.0631(14)	0.0552(14)	−0.0198(11)	−0.0150(10)	0.0114(11)
C16	0.1307(4)	−0.2030(3)	1.0122(2)	0.0760(18)	0.0673(16)	0.0644(16)	−0.0274(13)	−0.0273(13)	0.0091(13)
C17	0.0947(4)	−0.2897(3)	1.1360(3)	0.086(2)	0.0576(15)	0.0698(17)	−0.0142(13)	−0.0263(15)	0.0081(13)
C18	0.1407(4)	−0.2406(3)	1.2169(2)	0.0781(19)	0.0759(18)	0.0568(15)	−0.0179(13)	−0.0265(13)	0.0190(14)
C19	0.2228(4)	−0.1056(3)	1.1750(3)	0.084(2)	0.086(2)	0.0639(17)	−0.0300(15)	−0.0348(15)	0.0114(15)
C20	0.2588(4)	−0.0186(3)	1.0522(2)	0.0680(17)	0.0693(16)	0.0659(16)	−0.0244(13)	−0.0253(13)	0.0021(13)

Table S10. Atomic coordinates and displacement parameters (Å^2) for 4-((*E*)-3-oxobut-1-en-1-yl)phenyl cinnamate. Compound **8**.

	x	y	z	U11	U22	U33	U23	U13	U12
O1	0.05200(6)	0.8672(3)	0.09397(5)	0.0483(6)	0.0916(9)	0.0582(7)	0.0147(6)	0.0230(5)	0.0029(6)
O2	0.12666(7)	0.5815(4)	0.18862(7)	0.0635(8)	0.1317(12)	0.0810(8)	0.0430(9)	0.0344(7)	0.0164(7)
C1	0.12022(9)	0.7433(4)	0.13961(9)	0.0529(9)	0.0744(11)	0.0535(9)	0.0016(8)	0.0212(8)	−0.0012(8)
C2	0.18077(9)	0.8307(4)	0.11857(8)	0.0534(9)	0.0679(11)	0.0580(9)	0.0038(8)	0.0231(8)	−0.0024(8)
C3	0.25127(9)	0.7194(4)	0.15217(9)	0.0552(10)	0.0682(10)	0.0578(9)	0.0020(8)	0.0216(8)	−0.0018(8)
C4	0.31704(9)	0.7787(4)	0.13661(8)	0.0515(9)	0.0569(9)	0.0592(9)	0.0075(8)	0.0214(8)	−0.0047(7)
C5	0.38830(9)	0.6579(5)	0.18087(9)	0.0556(10)	0.0825(12)	0.0689(11)	0.0020(9)	0.0228(8)	0.0020(9)
C6	0.45123(10)	0.7052(5)	0.16764(11)	0.0520(10)	0.0909(14)	0.0900(13)	0.0074(11)	0.0256(10)	0.0018(9)
C7	0.44483(11)	0.8719(5)	0.11012(11)	0.0633(12)	0.0863(14)	0.1055(15)	0.0110(12)	0.0484(11)	−0.0095(10)
C8	0.37465(12)	0.9943(5)	0.06524(11)	0.0814(13)	0.0872(14)	0.0880(13)	0.0055(11)	0.0472(11)	−0.0064(11)
C9	0.31144(10)	0.9488(4)	0.07823(9)	0.0562(9)	0.0688(11)	0.0726(11	0.0032(9)	0.0257(8)	−0.0007(8)
C10	0.01369(9)	0.8028(4)	0.10433(8)	0.0510(9)	0.0654(10)	0.0527(9)	0.0095(8)	0.0260(7)	0.0077(7)
C11	0.02211(10)	0.9222(4)	0.16110(9)	0.0701(11)	0.0770(12)	0.0579(10)	0.0024(9)	0.0264(8)	0.0076(9)
C12	0.09008(12)	0.8665(5)	0.16624(10)	0.0882(13)	0.0876(13)	0.0670(11)	0.0154(10)	0.0492(11)	0.0317(11)
C13	0.14920(10)	0.6911(5)	0.11594(10)	0.0605(11)	0.0764(12)	0.0804(12)	0.0245(10)	0.0401(10)	0.0213(9)
C14	0.13847(10)	0.5790(5)	0.05933(9)	0.0520(9)	0.0800(12)	0.0754(11)	0.0035(9)	0.0276(8)	0.0025(8)
C15	0.07130(9)	0.6337(4)	0.05342(8)	0.0540(9)	0.0762(11)	0.0551(9)	0.0004(8)	0.0248(8)	0.0057(8)
C16	0.2277(2)	0.5980(11)	0.1108(2)	0.057(2)	0.074(2)	0.064(2)	0.0050(19)	0.028(2)	0.0077(18)
C17	0.25181(17)	0.6739(8)	0.15888(16)	0.0595(16)	0.094(2)	0.0694(18)	0.0042(15)	0.0322(14)	0.0081(14)
C18	0.3275(2)	0.5985(12)	0.1546(2)	0.0650(18)	0.102(3)	0.076(3)	0.011(2)	0.043(2)	0.0050(17)
C19	0.3852(4)	0.428(3)	0.0955(4)	0.079(3)	0.125(4)	0.127(5)	0.030(4)	0.063(3)	−0.021(3)
O3	0.3440(2)	0.6979(11)	0.20009(18)	0.0830(19)	0.162(4)	0.102(2)	0.0204(19)	0.0562(15)	−0.002(2)
C16A	0.2078(3)	0.6756(19)	0.1415(3)	0.059(3)	0.084(4)	0.059(3)	0.010(3)	0.031(3)	0.013(2)
C17	0.2735(3)	0.5249(14	0.1018(3)	0.065(3)	0.092(3)	0.077(3)	0.006(2)	0.036(2)	0.006(2)
C18A	0.3382(3)	0.4873(19	0.1185(3)	0.064(3)	0.105(4)	0.088(4)	0.008(3)	0.040(3)	0.002(3)
C19A	0.3335(5)	0.572(3)	0.1854(4)	0.087(4)	0.115(6)	0.089(4)	0.008(4)	0.048(3)	0.026(4)
O3A	0.3981(5)	0.366(4)	0.0741(5)	0.098(4)	0.171(7)	0.115(5)	0.020(4)	0.047(3)	0.039(4)

Table S11. Atomic coordinates and displacement parameters (Å^2) for 3-((*E*)-3-oxobut-1-en-1-yl)phenyl cinnamate. Compound **9**.

	x	y	z	U11	U22	U33	U23	U13	U12
O1	0.24020(4)	0.2592(2)	0.12000(18)	0.0432(8)	0.0481(8)	0.0817(10)	−0.0014(7)	0.0044(7)	−0.0022(6)
O2	0.20497(5)	0.5635(3)	0.2063(2)	0.0684(11)	0.0481(10)	0.1150(14)	−0.0111(9)	0.0264(9)	−0.0057(7)
O3	0.44225(5)	−0.0922(3)	0.3741(3)	0.0667(11)	0.0723(12)	0.1323(16)	0.0165(11)	−0.0035(10)	0.0103(9)
C1	0.20817(6)	0.3590(4)	0.1845(3)	0.0454(12)	0.0499(13)	0.0638(13)	−0.0015(10)	−0.0012(9)	0.0012(10)
C2	0.17908(6)	0.1821(3)	0.2234(2)	0.0471(12)	0.0452(11)	0.0658(13)	0.0008(10)	−0.0036(10)	−0.0007(9)
C3	0.14329(6)	0.2458(3)	0.2648(2)	0.0531(13)	0.0460(12)	0.0604(13)	0.0005(9)	0.0023(10)	−0.0001(9)
C4	0.11156(6)	0.0952(3)	0.3183(2)	0.0509(12)	0.0467(11)	0.0489(11)	−0.0031(9)	0.0004(9)	−0.0010(9)
C5	0.07164(6)	0.1659(4)	0.2934(3)	0.0534(13)	0.0578(13)	0.0704(14)	−0.0033(11)	0.0075(10)	0.0045(10)
C6	0.04126(7)	0.0268(4)	0.3432(3)	0.0519(14)	0.0806(18)	0.0847(17)	−0.0098(14)	0.0144(12)	−0.0016(12)
C7	0.05044(8)	−0.1810(5)	0.4210(3)	0.0707(17)	0.0783(18)	0.0712(15)	−0.0070(13)	0.0209(12)	−0.0206(14)
C8	0.08988(7)	−0.2536(4)	0.4473(3)	0.0794(17)	0.0617(14)	0.0587(14)	0.0065(11)	0.0043(12)	−0.0125(12)
C9	0.12017(7)	−0.1168(4)	0.3958(3)	0.0569(13)	0.0556(13)	0.0590(13)	0.0012(10)	−0.0023(10)	−0.0017(10)
C10	0.27320(6)	0.4008(3)	0.0875(2)	0.0465(12)	0.0477(12)	0.0532(12)	−0.0027(9)	0.0042(9)	−0.0025(9)
C11	0.31041(6)	0.3225(3)	0.1538(2)	0.0484(11)	0.0433(11)	0.0510(11)	0.0014(9)	0.0067(9)	0.0037(9)
C12	0.34479(6)	0.4513(3)	0.1275(2)	0.0491(12)	0.0492(12)	0.0464(11)	−0.0026(9)	0.0087(9)	0.0007(9)
C13	0.33992(6)	0.6535(4)	0.0335(2)	0.0565(13)	0.0557(13)	0.0569(12)	0.0005(10)	0.0122(10)	−0.0057(10)
C14	0.30235(7)	0.7247(4)	−0.0343(3)	0.0687(15)	0.0576(13)	0.0587(13)	0.0127(10)	0.0081(11)	0.0028(11)
C15	0.26843(6)	0.5982(4)	−0.0077(2)	0.0541(13)	0.0593(14)	0.0567(13)	0.0076(10)	−0.0019(10)	0.0058(10)
C16	0.38518(6)	0.3818(4)	0.1988(2)	0.0452(11)	0.0589(13)	0.0574(12)	−0.0006(10)	0.0093(9)	−0.0042(10)
C17	0.39593(6)	0.1753(4)	0.2561(3)	0.0479(12)	0.0607(14)	0.0629(13)	0.0006(11)	0.0054(10)	−0.0038(10)
C18	0.43642(6)	0.1074(4)	0.3308(3)	0.0481(13)	0.0693(16)	0.0638(13)	0.0021(11)	0.0055(10)	0.0062(11)
C19	0.46898(7)	0.2843(5)	0.3540(3)	0.0524(14)	0.0879(18)	0.0980(19)	0.0023(15)	−0.0030(12)	−0.00

Table S12. Atomic coordinates and displacement parameters (Å^2) for 2-((*E*)-3-oxobut-1-en-1-yl)phenyl cinnamate. Compound **10**.

	x	y	z	U11	U22	U33	U23	U13	U12
O1	0.0677(6)	0.6038(2)	0.08252(19)	0.078(3)	0.084(3)	0.057(2)	−0.002(2)	0.000(2)	−0.020(2)
O2	0.3081(6)	0.6869(2)	0.15277(19)	0.093(3)	0.095(3)	0.059(2)	−0.018(2)	0.001(2)	−0.018(2)
C1	0.1339(11)	0.6447(4)	0.1456(3)	0.090(5)	0.060(4)	0.064(4)	0.014(4)	0.014(4)	0.013(4)
C2	−0.0532(10)	0.6271(3)	0.2042(3)	0.093(5)	0.080(4)	0.064(4)	0.001(3)	−0.017(4)	−0.013(4)
C3	−0.0420(9)	0.6584(3)	0.2682(3)	0.082(4)	0.060(4)	0.070(4)	−0.003(3)	−0.029(4)	0.005(3)
C4	−0.2119(9)	0.6451(3)	0.3291(3)	0.060(4)	0.063(4)	0.053(4)	0.014(3)	0.011(3)	0.001(3)
C5	−0.4105(10)	0.5931(3)	0.3222(3)	0.074(4)	0.078(4)	0.060(4)	0.002(3)	0.003(3)	0.000(3)
C6	−0.5619(9)	0.5824(3)	0.3811(3)	0.074(4)	0.085(4)	0.074(4)	0.013(4)	0.003(4)	−0.011(4)
C7	−0.5167(9)	0.6247(3)	0.4466(3)	0.071(5)	0.090(5)	0.078(4)	0.013(4)	0.028(4)	0.003(4)
C8	−0.3214(10)	0.6772(3)	0.4536(3)	0.101(5)	0.095(5)	0.062(4)	−0.005(3)	0.019(4)	−0.003(4)
C9	−0.1723(9)	0.6869(3)	0.3952(3)	0.075(4)	0.081(4)	0.072(4)	−0.003(4)	0.007(4)	−0.014(3)
C10	0.2159(9)	0.6119(3)	0.0217(2)	0.065(4)	0.071(4)	0.034(3)	−0.003(3)	−0.001(3)	−0.018(3)
C11	0.4146(10)	0.5594(3)	0.0173(3)	0.067(4)	0.058(4)	0.062(4)	−0.008(3)	−0.014(3)	−0.001(3)
C12	0.5458(9)	0.5686(3)	−0.0466(3)	0.064(4)	0.079(4)	0.099(5)	−0.030(4)	−0.011(4)	0.017(4)
C13	0.4796(10)	0.6256(4)	−0.1024(3)	0.081(5)	0.086(5)	0.066(4)	−0.010(4)	0.011(4)	−0.009(4)
C14	0.2826(10)	0.6751(3)	−0.0952(3)	0.092(5)	0.067(4)	0.056(3)	0.009(3)	−0.013(4)	−0.013(4)
C15	0.1474(8)	0.6690(3)	−0.0323(3)	0.056(4)	0.066(4)	0.056(3)	0.004(3)	0.002(3)	−0.003(3)
C16	0.435(3)	0.4959(10)	0.0832(7)	0.065(6)	0.060(6)	0.052(7)	−0.011(5)	−0.004(5)	0.005(6)
C17	0.633(2)	0.4554(7)	0.0991(6)	0.078(6)	0.065(5)	0.060(5)	−0.006(3)	−0.009(5)	0.012(5)
C18	0.674(3)	0.3944(16)	0.1628(11)	0.093(6)	0.075(5)	0.067(5)	−0.001(4)	−0.013(6)	0.014(6)
C19	0.468(3)	0.3627(16)	0.2081(9)	0.114(6)	0.074(7)	0.057(10)	−0.009(7)	−0.012(7)	−0.006(7)
O3	0.872(2)	0.3685(10)	0.1731(7)	0.102(6)	0.126(6)	0.105(8)	0.042(6)	−0.020(6)	0.024(6)
C16A	0.545(3)	0.4971(12)	0.0640(9)	0.077(7)	0.066(6)	0.048(6)	−0.012(4)	−0.006(6)	0.003(7)
C17A	0.449(3)	0.4577(8)	0.1182(7)	0.078(6)	0.068(6)	0.054(6)	−0.005(4)	−0.005(5)	0.006(6)
C18A	0.596(3)	0.3955(17)	0.1628(12)	0.091(6)	0.075(5)	0.064(5)	−0.003(4)	−0.014(5)	0.010(6)
C19A	0.481(4)	0.3615(19)	0.2329(11)	0.118(8)	0.086(8)	0.056(10)	0.006(8)	−0.024(7)	0.008(7)
O3A	0.794(2)	0.3776(13)	0.1469(9)	0.095(8)	0.130(8)	0.091(8)	0.021(7)	−0.020(6)	0.027(7)
